# Drop the shortcuts: image augmentation improves fairness and decreases AI detection of race and other demographics from medical images

**DOI:** 10.1016/j.ebiom.2024.105047

**Published:** 2024-03-11

**Authors:** Ryan Wang, Po-Chih Kuo, Li-Ching Chen, Kenneth Patrick Seastedt, Judy Wawira Gichoya, Leo Anthony Celi

**Affiliations:** a Department of Computer Science, National Tsing Hua University, Hsinchu, Taiwan; b Department of Surgery, Beth Israel Deaconess Medical Center, Harvard Medical School, Boston, MA, USA; c Department of Thoracic Surgery, Roswell Park Comprehensive Cancer Center, Buffalo, NY, USA; d Department of Radiology, Emory University, Atlanta, GA, USA; e Laboratory for Computational Physiology, Massachusetts Institute of Technology, Cambridge, MA, USA; f Division of Pulmonary Critical Care and Sleep Medicine, Beth Israel Deaconess Medical Center, Boston, MA, USA; g Department of Biostatistics, Harvard T.H. Chan School of Public Health, Boston, MA, USA

**Keywords:** Bias mitigation, Shortcuts, Augmentation, Fairness, Deep learning

## Abstract

**Background:**

It has been shown that AI models can learn race on medical images, leading to algorithmic bias. Our aim in this study was to enhance the fairness of medical image models by eliminating bias related to race, age, and sex. We hypothesise models may be learning demographics via shortcut learning and combat this using image augmentation.

**Methods:**

This study included 44,953 patients who identified as Asian, Black, or White (mean age, 60.68 years ±18.21; 23,499 women) for a total of 194,359 chest X-rays (CXRs) from MIMIC-CXR database. The included CheXpert images comprised 45,095 patients (mean age 63.10 years ±18.14; 20,437 women) for a total of 134,300 CXRs were used for external validation. We also collected 1195 3D brain magnetic resonance imaging (MRI) data from the ADNI database, which included 273 participants with an average age of 76.97 years ±14.22, and 142 females. DL models were trained on either non-augmented or augmented images and assessed using disparity metrics. The features learned by the models were analysed using task transfer experiments and model visualisation techniques.

**Findings:**

In the detection of radiological findings, training a model using augmented CXR images was shown to reduce disparities in error rate among racial groups (−5.45%), age groups (−13.94%), and sex (−22.22%). For AD detection, the model trained with augmented MRI images was shown 53.11% and 31.01% reduction of disparities in error rate among age and sex groups, respectively. Image augmentation led to a reduction in the model's ability to identify demographic attributes and resulted in the model trained for clinical purposes incorporating fewer demographic features.

**Interpretation:**

The model trained using the augmented images was less likely to be influenced by demographic information in detecting image labels. These results demonstrate that the proposed augmentation scheme could enhance the fairness of interpretations by DL models when dealing with data from patients with different demographic backgrounds.

**Funding:**

10.13039/100020595National Science and Technology Council (Taiwan), 10.13039/100000002National Institutes of Health.

**Research in context**

**Evidence before this study**

We used Google Scholar and PubMed search engines to do our review. We used keywords “Fairness”, “Shortcut learning”, “Machine learning in healthcare”, and “Medical image” to query the articles on Google Scholar. We used the following terms “(((disparity OR bias OR fairness OR shortcuts) AND (classification)) AND ((x-ray) OR (MRI))) AND (machine learning [MeSH Terms] OR deep learning [MeSH Terms])” on PubMed. We limited the articles to original research and English articles. We excluded the articles which were written before January 1 2010, and did not focus on medical imaging. Previous works have discussed the bias in medical imaging classification tasks and demonstrated the discrepancy in performance in demographic groups. Gichoya and colleagues have shown that the deep learning (DL) model could recognise the race of the patients by CXR with exceptional accuracy and that could be a potential source of the disparity in performance of healthcare AI. DeGrave and colleagues have shown that the DL models may exploit the token in CXR images as shortcuts in COVID-19 detection. Zhang and colleagues benchmarked several debias methods during the training phase in improving the fairness of the classifier. Jabbour and their colleagues tried to prevent the shortcuts and improve fairness in medical imaging fields by implementing transfer learning approaches. In computer vision applications, Chung and colleagues proposed a data augmentation method to achieve group fairness. Tian and colleagues demonstrated several research methods that implement data augmentation to solve fairness issues. However, to the best of our knowledge, no study has focused on using image augmentation to enhance the fairness of AI in medical imaging.

**Added value of this study**

The augmentation is an unsupervised, model-agnostic, and data-agnostic approach and can be applied in either training or test phases. In this study, we mitigated the effect of demographic attributes contributing to model decision-making for disease prediction. First, we showed that the augmented images weaken the performance of DL models in classifying demographics by learning fewer demographic attributes. The augmentation schemes were implemented and validated on two publicly available CXR datasets and a brain MRI dataset. Second, models trained using augmented images maintained good performance in radiological finding or neurological disorder detection while reducing disparities in several evaluation metrics among demographic groups. We compared our method to several debiasing methods using various evaluation metrics. Finally, our experiment objectively showed that augmenting the images prevents a DL classifier from learning demographic features for pathology detection.

**Implications of all the available evidence**

In our study, we focus on lowering the disparity in AUC, binary cross entropy (BCE), expected calibration error (ECE), error rate, and precision by augmenting the images before the DL model training process. In contrast to current methodologies for mitigating bias, our proposed approach is noteworthy for its model-agnostic and task-agnostic characteristics, coupled with the absence of a dependency on auxiliary demographic labels. This augmentation strategy demonstrates a capacity to diminish disparities while concurrently sustaining model performance. Although our method does not entirely eradicate disparities, it accentuates the imperative for further investigative efforts in this relatively nascent domain, particularly in the context of the escalating application of DL within the medical sector. Human researchers are unable to detect biases from imaging alone, and we must further understand how algorithms are learning biases and perpetuating them to combat this issue. This study indicates some evidence we can combat algorithmic bias through data augmentation and preventing shortcuts; still, work still needs to be done to completely remove bias that could potentiate racial disparities prevalent today.

## Introduction

Computer-aided diagnosis (CAD) and deep learning (DL) have proven highly effective in pathologic diagnosis^1^^,^^2^ (radiological finding detection), anatomical segmentation on chest X-rays (CXR),^3^^,^^4^ detecting Alzheimer's disease (AD),^5^^,^^6^ and segmenting brain regions on magnetic resonance imaging (MRI).^7^^,^^8^ DL models have also demonstrated remarkable performance in augmenting clinical decision making and assisting researchers to better utilise clinical data for tasks such as medical imaging classification,^7^^,^^8^ personalised risk prediction in electronic health records (EHR),^9^^,^^10^ and analysing physiological data.^11^, ^12^, ^13^ Despite these advances, fairness in healthcare DL models is a growing concern.^13^, ^14^, ^15^ Defined here, fairness represents an algorithmic bias present in model predictions, and an example would be an unfair model creating unfavourable predictions based on race, sex, or age from training data. A growing number of researchers are addressing fairness and detecting algorithmic biases in the application of DL models for various healthcare applications.^16^, ^17^, ^18^, ^19^, ^20^ A recent study has benchmarked several debias methods in improving the fairness of the healthcare model.^21^

One potential source of algorithm bias has been uncovered from previous studies that have demonstrated DL models are prone to shortcuts based on the oversimplification of data features.^22^^,^^23^ For example, using an image dataset from a single hospital with a high prevalence of pneumonia to train a model could result in the ubiquitously used metal marker placed by the radiology technician in the corner of the chest radiograph to be prioritised over the more complex shapes and patterns indicative of true pathologic pneumonia. Similar situations might arise when a machine learning model focuses on features that are typical of race, age, or sex, rather than pathological phenomena. For example, one breast histology algorithm reflected ethnicity rather than intrinsic tumour biology based on histologic staining patterns at a particular site with more Black patients than other participating institutions.^24^ Further studies demonstrated that convolution neural networks (CNNs) were shown to generate results that varied as a function of race, age, sex, or socioeconomic status, thereby exposing patients to potentially erroneous predictions.^16^^,^^18^ Importantly, one recent study^25^ reported that DL models are highly effective in differentiating among individuals of different races, based on chest radiographs, cervical spine radiographs, and computed tomography (CT) scans of the chest. In that study, DL models achieved high area under the receiver operating characteristic (ROC) curve (AUC) scores (0.80–0.99) even when trained using images of low quality, segmented regions, or other perturbations. These biases can seriously compromise prediction accuracy in real-world settings as the models are making predictions based on unintended patterns, hindering model generalizability.

The ease with which machine learning models identify race from patient data such as CXR images raises the possibility of using these features as shortcuts in identifying pathological features and thereby affecting the fairness of the models by introducing and perpetuating bias. Researchers remain in the dark when it comes to understanding the means by which machine learning models identify race, thereby making it very challenging to improve fairness and eliminate race-related bias from diagnostic results.^25^ One hypothesis is that algorithms are taking shortcuts (shortcut learning) and is a problem of inadequate generalizability.^26^ Data augmentation, widely used in machine learning for a range of data types,^27^, ^28^, ^29^, ^30^, ^31^ can reduce the effects of overfitting,^27^^,^^31^ underperformance,^28^ and generalizability.^31^, ^32^, ^33^ It attempts to extract more information from the original training data set by artificially expanding the training set through warping images or oversampling.^29^^,^^33^ Multiple studies have demonstrated that data augmentation can effectively eliminate learned shortcuts from the original dataset.^34^, ^35^, ^36^ This is further evidenced by a recent study employing an adversarial U-Net architecture to alter natural images, thereby removing shortcut features.^36^ If shortcut learning potentiates bias in healthcare DL algorithms, data augmentation may assist in improving model fairness by counteracting shortcut learning.

In the current study, we sought to improve model fairness by preventing a DL model from learning shortcuts using data augmentation. Our objective was to eliminate disparities in detection performance in medical images among demographic groups (e.g., Black vs. White, male vs. female, or young vs. old).

## Methods

### Dataset

This study was based on 2D images in two CXR datasets and 3D images in a brain imaging dataset. We collected de-identified CXR images and clinical data in the MIMIC-CXR v2.0.0 database,^37^, ^38^, ^39^ a retrospective CXR database containing over 220,000 CXR images from patients admitted to the emergency department between 2011 and 2016. The MIMIC-IV^40^, ^41^, ^42^ database, a retrospective EHR database containing data from over 40,000 patients admitted to the intensive care unit at Beth Israel Deaconess Medical Center from 2008 to 2019, was used to extract the corresponding demographic attributes of patients in MIMIC-CXR. The institutional review boards of the Massachusetts Institute of Technology (No. 0403000206) and Beth Israel Deaconess Medical Center (2001-P-001699/14) both approved the assembly of the database for research. The CheXpert^43^ database is a large public CXR database containing 224,316 chest radiographs labelled with 14 radiological findings (labels) from 65,240 patients. Frontal CXR images retrospectively retrieved from MIMIC-CXR (2011–2016) were used for whole experiments, whereas radiographs from CheXpert (2002–2017) were used for external validation. The radiological findings of each CXR image was extracted from the free-text radiology report using rule-based natural language processing models (NegBio^44^ and CheXpert^43^). Radiological findings for CXR images were: ‘Atelectasis’, ‘Cardiomegaly’, ‘Consolidation’, ‘Edema’, ‘Enlarged Cardiomediastinum’, ‘Fracture’, ‘Lung Lesion’, ‘Lung Opacity’, ‘No Finding’, ‘Pleural Effusion’, ‘Pleural Other’, ‘Pneumonia’, ‘Pneumothorax’, and ‘Support Devices’. Three types of demographic attributes were extracted: self-identified race, age, and self-reported sex. As shown in Table 1, this study included MIMIC-CXR images from 44,953 patients who identified themselves as Asian, Black, or White (mean age, 60.68 years ±18.21; 23,499 (52.3%) women) for a total of 194,359 radiographs. This study also included CheXpert images of 45,095 patients (mean age 63.10 years ±18.14; 20,437 (45.3%) women) for a total 134,300 radiographs. We used the CheXpert as an external validation cohort due to the different distribution of races. We considered Asian, Black, and White because of the larger populations in both MIMIC-CXR and CheXpert databases. We excluded three radiological findings (‘Fracture’, ‘Lung Lesion’, and ‘Pleural Other’) because of the data scarcity and excluded ‘Support Devices’ because of its low clinical relevance.

**Table 1:** Datasets used in the current study.

**Attributes**	MIMIC-CXR	CheXpert	**Attributes**	ADNI
**Type**	CXR	CXR	**Type**	Brain MRI
**# Images**	194,359	134,300	**# Images**	1195
**# Patients**	44,953	45,095	**# Patients**	272
**Race**			**Race**	
Asian	1941 (4.3%)	7422 (16.5%)	Asian	1 (0.4%)
Black	8945 (19.9%)	3016 (6.7%)	Black	21 (7.7%)
White	34,067 (75.8%)	34,657 (76.9%)	White	248 (91.2%)
Others	N/A	N/A	Others	2 (0.7%)
**Sex**			**Sex**	
Female	23,499 (52.3%)	20,437 (45.3%)	Female	142 (52.2%)
Male	21,454 (47.7%)	24,657 (54.7%)	Male	130 (47.8%)
**Age**			**Age**	
0–40	6390 (14.2%)	5644 (12.5%)	0–75	110 (40.4%)
40–60	13,680 (30.4%)	13,316 (29.5%)		
60–80	17,095 (38.0%)	17,599 (39.0%)	75+	162 (59.6%)
80+	7788 (17.3%)	8536 (18.9%)		

The 3D brain MRI data used in the preparation of this article were obtained from the Alzheimer's Disease Neuroimaging Initiative (ADNI)^45^ database (adni.loni.usc.edu). The ADNI was launched in 2003 as a public-private partnership, led by Principal Investigator Michael W. Weiner, MD. We extracted the preprocessed scans with NIFTI format, which had undergone image preprocessing steps including multiplanar reconstruction (MPR), GradWarp, and B1 non-uniformity correction. We collected a total of 272 patients which were labelled as either Alzheimer's Disease (AD) or Cognitively Normal (CN). As shown in Table 1, the cohort included 272 patients (mean age 76.97 years ±14.22; 142 women) for a total 1195 brain MRI images. Because the race distribution was imbalanced (91.2% are White), we only separated groups by age and sex in our following experiments. The augmented image dataset was created by distorting all images via random rotation, shear transformation, scaling transformation, and fisheye distortion.

In MIMIC-CXR, the dataset was split into subsets for training (116,405 radiographs, 60%), validation (119,339 radiographs, 10%), and testing (58,618 radiographs, 30%). All images underwent histogram equalisation and resizing to (224, 224) before being written to TFrecords to ensure data consistency. In ADNI, the dataset was split into training (765 images, 64%), validation (187 images, 15.6%), and testing (243 images, 20.3%) sets. All MRI images were segmentented using SPM 12 (https://www.fil.ion.ucl.ac.uk/spm/) and only the gray matter, white matter and cerebrospinal fluid were preserved. The segmented images were centre cropped according to the brain area and resized to (96, 96, 96). The random seed was set to 2021 for all analyses to ensure reproducibility. We partitioned the data into training, validation, and testing sets according to subjects, thereby ensuring that no data leakage occurred. The detailed data information regarding the train, validation, and test splits are shown in Supplementary Tables H1 and H2.

### Experiment overview

**Fig. 1:**
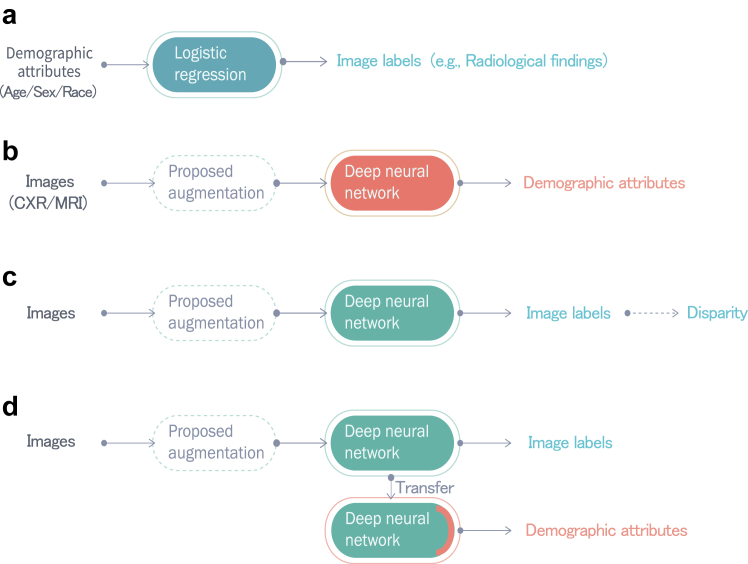
Experiments performed in the current study. Experiment A: Radiological/AD label detection based on demographic attributes via logistic regression. Experiment B: Demographic attribute prediction from CXR and brain MRI images via the CNN-based model. Detection performance was used to indicate the presence of demographic features in CXR or brain MRI images, which could potentially be used as shortcuts. Experiment C: Testing for disparities in radiological/AD label detection results among demographic groups when applying a Densenet121-based model to CXR images and a ResNet 18-based model to brain MRI images. Experiment D (Task transfer test): The trained model in Experiment C would be frozen and the last prediction layer would be replaced to classify demographic attributes. The model's performance was used to indicate whether the model had incorporated demographic features as shortcuts in the radiological/AD label detection task. The proposed augmentation method was then applied to Experiment B–D and compared with the results obtained without augmentation.

Fig. 1 illustrates the four experiments conducted in the current study. (A) We assessed the correlation between image labels (radiological finding or disease) and demographics. (B) We assessed the performance of a DL model in differentiating demographics with and without augmented images. The performance was an indication of the presence of the learned demographic features in images, which may be exploited as shortcuts by the DL model. (C) We computed disparities across racial, age, and sex subgroups in detecting image labels (i.e., AD or radiological findings) to assess the extent to which the predictions of the DL model exhibited bias. (D) We conducted a task transfer experiment in which feature representations embedded in the trained model in (C) were used to predict demographic attributes. The performance was used to indicate whether the model had incorporated demographic features as shortcuts in the image label detection task.^46^ We then evaluated for improvement of fairness in Experiments B, C, and D, the results of which were compared with those obtained without augmentation.

### Proposed augmentation

As shown in the dashed boxes in Fig. 1, which illustrates Experiments B, C, and D, the proposed augmentation scheme involved distorting images via random rotation, shear transformation, scaling transformation, and fisheye distortion. Image rotation was implemented using random angles between −90° and 90° for CXR and between −10° and 10° for brain MRI. The shear transformation was implemented using random radians between −***π***/4 and ***π***/4 for CXR and between −***π***/6 and ***π***/6 for brain MRI. Image scaling was implemented using randomly selected sizes of between 0.4 and 1 for CXR and between 0.8 and 1 for brain MRI. Fisheye distortion^47^ was implemented with the coefficient set to 0.4 with a randomly selected central point for CXR and brain MRI. Details pertaining to the four augmentations are listed in Supplementary Table A1. Table 2 presents the example of applying a single augmentation to an CXR image. Supplementary Table A2 shows the example of an augmented brain MRI image.

**Table 2:** Examples of image distortion methods used in this study (one distortion per image).

Methods	Examples		
Rotating transformation	Angle: −20°	Angle: 45°	Angle: 90°
		
Shear transformation	Radian: ***π***/6	Radian: −***π***/5	Radian: −***π***/4
		
Scaling transformation	Size: 0.8	Size: 0.6	Size: 0.4
		
Fisheye distortion	Central point: (133, 164)	Central point: (176, 105)	Central point: (110, 92)
		

### Experiment A: relationship between demographic attributes and image labels

In Experiment A, the mutual independence of demographic attributes (race, age, and sex) and image labels (radiological findings for CXR, AD for brain MRI) was evaluated using the chi-square test and permutation test.

### Experiment B: demographic attribute identification from images

Experiment B involved implementing trained models within the DenseNet121^48^ initialised with ImageNet pre-trained weights and 3D ResNet 18^49^^,^^50^ without pre-trained weights for CXR and brain MRI, respectively. For the classification of race/age/sex from CXR, we added a Softmax classification layer with three outputs for race (Asian, Black, and White), four outputs for age (0–40, 40–60, 60–80, and 80-), and two outputs for sex (male and female), and the Adam optimizer was used to optimise categorical cross entropy loss. For the classification of age/sex from brain MRI, we added a single node sigmoid prediction layer for age (0–75 and 75-) and sex (male and female), and the binary cross entropy (BCE) loss was used. The number of epochs depended on the specifics of the training process. Training for the CXR data was discontinued if the validation loss did not show improvement over 4 consecutive epochs within a span of 15 epochs. Similarly, for the brain MRI data, training ceased when there was no improvement in validation loss across 10 consecutive epochs within a total of 80 epochs. The batch size was set to 128 and 16 for CXR and brain MRI, respectively. The learning rate decayed by 5% per 2 epochs with an initial learning rate of 0.001.

### Experiment C: disparities of radiological findings and AD detection in images

Experiment C involved the detection of radiological findings or disease in images. For CXR, we added a sigmoid classification layer with ten nodes corresponding to 10 radiological findings. Because the ten labels were independent, we performed a multi-label classification task by optimising BCE loss. For brain MRI, we performed a binary classification task for classifying AD and CN. The batch size, learning rate, and number of epochs were the same as the model used for the classification of demographic attributes. Binary classification thresholds were selected for each radiological finding (or AD label) to maximise the weighted F1-score for the validation set. Test-time augmentation is a technique for obtaining ensemble effects and enhancing the performance by averaging the predictions over multiple augmented data.^51^ In this study, we employed our proposed augmentation scheme on the test set to simulate real-world scenarios where re-training a model is impractical. For each original image, we generated three augmented images using each augmentation method, resulting in a total of twelve augmented images. The final prediction was obtained by averaging the prediction scores for each of the augmented images.

### Experiment D: task transfer from image label detection to demographic attribute identification

To indicate whether the model had incorporated demographic features as shortcuts in the image label detection task, Experiment D involved predicting demographic attributes by using the hidden state from the penultimate layer of the radiological finding or AD detection model. Comparisons were performed on models trained with and without augmentation.

### Model interpretation

To gain insight into how the models perform image-based evaluations, we first used Gradient-weighted Class Activation Mapping (GradCAM)^52^ and integrated gradient^53^ to generate heatmaps for individual examples showing the regions on which the model focused. We further used mean saliency maps generated by integrated gradients, to show the regions on which the model focused for a set of images. We selected the images where the original model accurately identified the demographic attributes but the proposed model did not, while both models correctly recognised radiological findings.

### Comparison with existing debias methods

We implemented existing debiasing methods to compare the improvement of fairness with our proposed method. A brief comparison of the existing methods and the proposed method is summarised in Table 3. The existing methods included those training on the balanced dataset or stratified dataset,^21^ using adversarial learning,^21^^,^^54^^,^^55^ penalising with the distribution distance, and using FairALM algorithms. We also applied our proposed methods to the existing methods to see if our proposed method could further improve the fairness.

**Table 3:** Summary of the existing debias methods and the proposed method.

Method	Implementation	Does it require demographic information?	Does it require re-training the model?	Difficulty
Baseline	Do not consider the demographic group differences.			
Balanced^21^	Reduce the sample size of the majority group to achieve a balanced population for each group.	Yes	Yes	The amount of data decreases.
Stratified^21^	Train separate models for each group.	Yes	Yes	Minority groups may have insufficient data, resulting in a poorly trained model.
Adversarial^54^^,^^56^	Use an adversary to an adversary to decrease the model's capacity to identify demographic groups.	Yes	Yes	Model-specific; determining the appropriate level of the adversary can be challenging.
DistMatch MMD^57^	Add a penalty to reduce the maximum mean discrepancy^58^ distance between groups	Yes	Yes	The data imbalance between demographic groups, different data splits, and distance metrics during training may lead to instability in calculating the distance.
DistMatch Mean^57^	Add a penalty to reduce the mean of the distribution between groups.			
FairALM^59^	Apply an augmented Lagrangian method to penalise the distribution discrepancy.	Yes	Yes	Different assumptions regarding distribution can yield varying results.
Proposed augmentation	Use image augmentation to prevent the model from learning shortcut based on demographic information	No	No^a^	Time-consuming when augmenting images

a It is not necessary to re-train the model as the image augmentation can be applied during the test phase.

### Evaluation metrics

We used the model performance and disparities in AUC, BCE, expected calibration error (ECE), error rate, and precision to evaluate the efficacy of our proposed model. AUC, BCE, and ECE are threshold-free metrics.^21^^,^^60^^,^^61^ Error rate and precision are threshold-required metrics used in group fairness criteria and are also known as equalised odds and equal opportunity. Details of evaluation metrics can be found in Supplementary Table I1.

### Statistics

In Experiment A, we employed the chi-square test to assess whether the distribution of image labels significantly differed across demographic groups. In the permutation test, we initially utilised a logistic regression (LR) model to predict the image labels based on demographic attributes. Subsequently, we compared the AUC of the LR model against that of randomly permuted image labels, achieved by shuffling the radiological labels 100,000 times. We established a significance level of 0.001 for testing. In Experiment B, the AUC with 95% confidence interval (CI) was calculated over 1000 bootstrap iterations. Throughout this process, we repeatedly sampled data from the entire testing dataset and tested the model to obtain the results. To assess the presence of a statistically significant difference, the Student's t-test was utilised. In Experiment C, we quantified the disparities across demographic groups by averaging values over 1000 bootstrap iterations. Comparisons were performed on models trained with and without augmentation. In Experiment D, the AUC with 95% CI was calculated using the bootstrap method.

### Ethics

The Institutional Review Board exempted this retrospective study from the written informed consent requirement, as the Act on medical research involving human subjects did not apply.

### Role of the funding source

The funding source had no role in the study design, data collection, data analyses, interpretation, or writing of the report.

## Results

### Experiment A: relationship between demographic attributes and image labels

For CXR, the results of the chi-square test revealed dependencies between all radiologic labels and demographic attributes (p < 0.01, chi-square test) except sex and two labels (“Cardiomegaly” and “Edema”). In the case of brain MRI analysis, the chi-square test indicated no significant association with AD, yielding p-values of 0.06 for age and 0.42 for sex (chi-square test). Through permutation testing, LR achieved significantly higher AUC for all but two of the ten radiological features (“Cardiomegaly” and “Edema”) with p < 0.01 (permutation test). However, in the case of brain MRI analysis, LR did not yield a significantly higher AUC compared to random permutation. Details of the statistical results are shown in Supplementary Tables B1–B3.

### Experiment B: demographic attribute identification from images

The models trained and tested using the original CXR images achieved high AUC values in the classification of images according to race, age, and sex (first row of Table 4). The AUC values obtained using the model trained and tested using the augmented data were 17% lower than those obtained using the original data in the detection of race, 16.4% lower in the detection of age, and 0.6% lower in the detection of sex. The t-test results indicate that the predictions have the statistically significant difference (p-value <0.001, t-test) for race, age, and sex. Table 5 shows the results of predicting age and sex using original and augmented MRI images. The proposed augmentation model was effective in preventing the model from recognizing the age (18.3% lower) and sex (35.2% lower) using brain MRI images. The t-test results indicate that the predictions have the statistically significant difference (p-value <0.001, t-test) for age, and sex. These results indicate that using augmented (distorted) images hindered the retrieval of demographic information. Disparities using only a single augmentation method can be found in Supplementary Tables A3–A8.

**Table 4:** AUCs of models with and without proposed augmentation in detecting demographic attributes in CXR.

Aug	Race			Age				Sex
	Asian	Black	White	0–40	40–60	60–80	80-	
w/o	0.937 [0.931–0.943]	0.954 [0.951–0.956]	0.950 [0.947–0.952]	0.965 [0.963–0.967]	0.834 [0.830–0.838]	0.795 [0.791–0.799]	0.899 [0.896–0.903]	0.992 [0.992–0.993]
w/	**0.712 [0.700–0.724]**	**0.826 [0.820–0.831]**	**0.821 [0.816–0.825]**	**0.843 [0.837–0.850]**	**0.678 [0.673–0.683]**	**0.581 [0.576–0.587]**	**0.818 [0.813–0.823]**	**0.986 [0.985–0.987]**

Lower values indicate a weaker ability to recognise race, age, or sex based on CXR images. The model trained using augmented images is hard to recognise demographic attributes from CXR. Aug.: Augmentation. The minimum values are highlighted in bold.

**Table 5:** AUCs of models with and without proposed augmentation in detecting demographic attributes in brain MRI.

Augmentation	Age (0–75 vs. 75+)	Sex (Female vs. Male)
w/o	0.655 [0.575–0.735]	0.856 [0.800–0.912]
w/	**0.535 [0.448**–**0.622]**	**0.555 [0.468**–**0.641]**

Lower values indicate a weaker ability to recognise age or sex based on brain MRI images. The model trained using augmented images is hard to recognise demographic attributes from brain MRI. The minimum values are highlighted in bold.

### Experiment C: performance and disparities in the detection of image labels

**Fig. 2:**
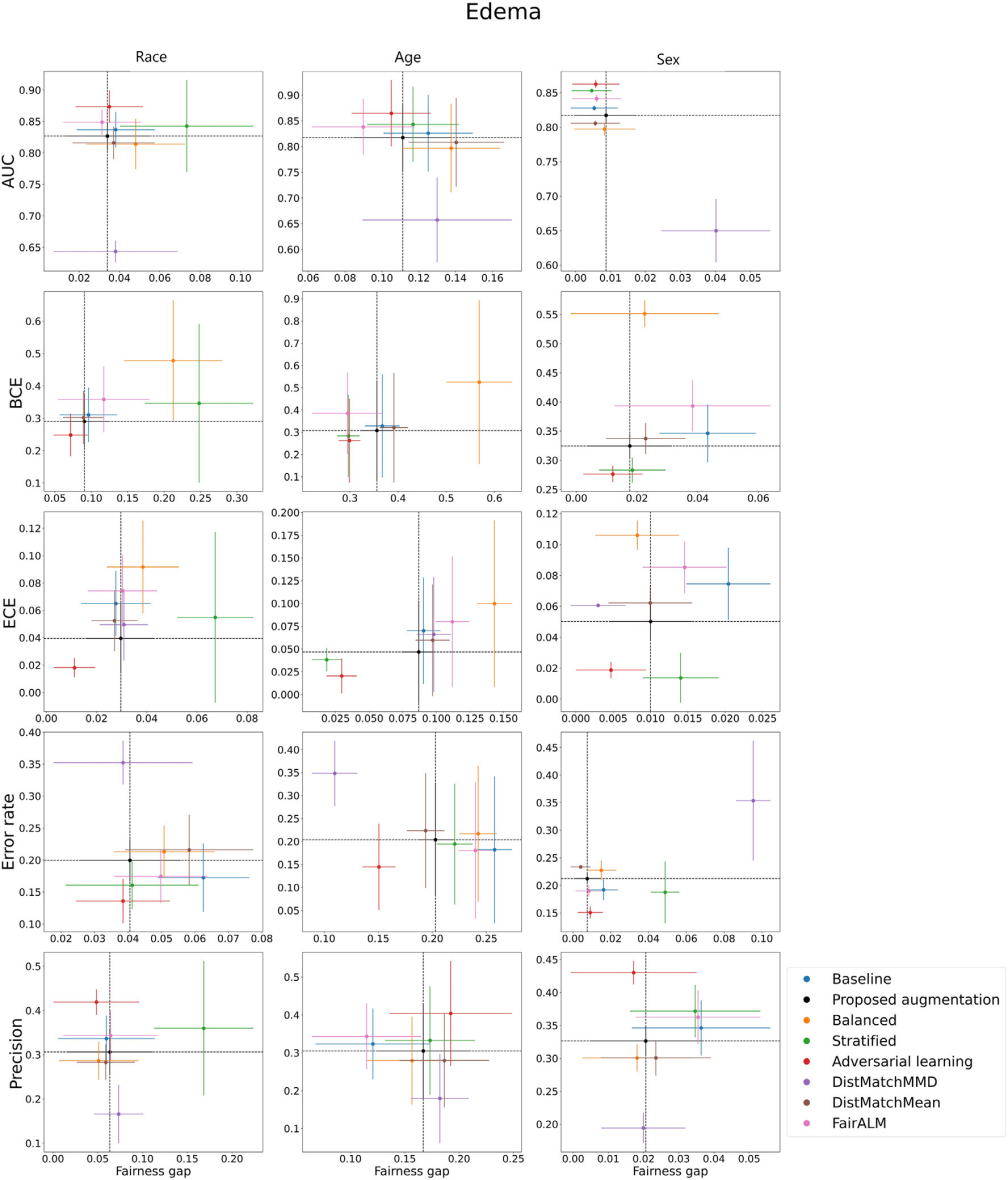
The model performance and fairness gap for identifying Edema from CXR images in different race, age, and sex groups. Each row represents the performance (y-axis) and fairness gap (x-axis) of each evaluation metric. The 95% confidence intervals are calculated from 1000 bootstrap iterations. Each plot represents a different de-bias technique including the baseline model, the proposed augmentation, balanced, stratified, adversarial learning, DistMatchMMD, DistMatchMean, and FairALM. AUC: Area Under the ROC Curve; BCE: Binary Cross Entropy; ECE: Expected Calibration Error.

**Fig. 3:**
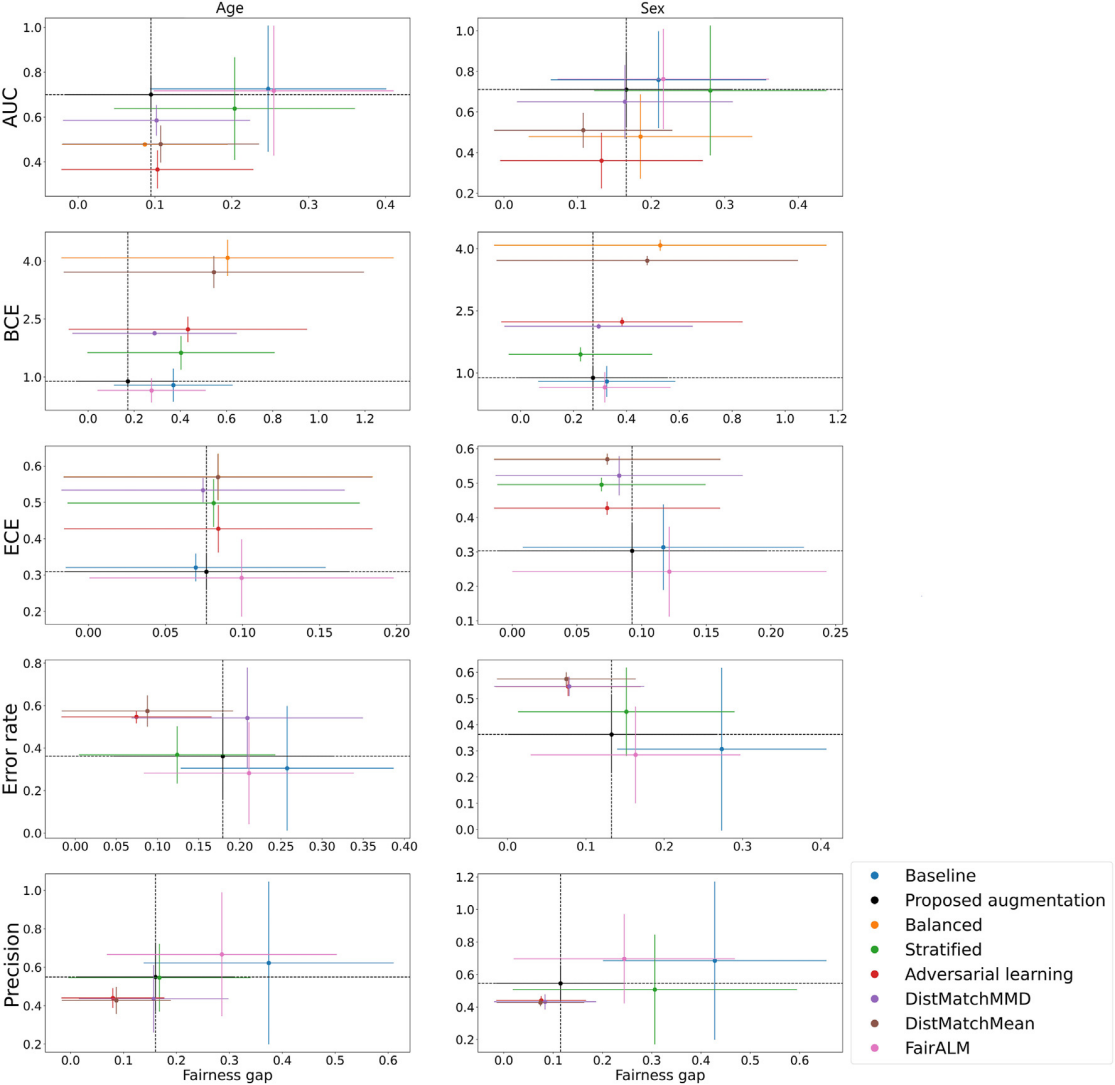
The model performance and fairness gap for identifying AD from brain MRI in different age and sex groups. Each row represents the performance (y-axis) and fairness gap (x-axis) of each evaluation metric. The 95% confidence intervals are calculated from 1000 bootstrap iterations. Each plot represents a different de-bias technique including the baseline model, the proposed augmentation, balanced, stratified, adversarial learning, DistMatchMMD, DistMatchMean, and FairALM. AUC: Area Under the ROC Curve; BCE: Binary Cross Entropy; ECE: Expected Calibration Error.

Figs. 2 and 3 illustrate the performance and fairness gaps of each method implemented. The fairness gap, as indicated on the x-axis, is presumed to measure the discrepancy in performance metrics across demographic groups, while the y-axis denotes the metric values.^62^ The dashed black lines represent the performance outcomes of the proposed model. For metrics such as AUC and precision, higher y-values signify better performance, whereas for BCE, ECE, and error rate, lower values are preferable. A smaller fairness gap denotes a more equitable model. Our proposed model demonstrates comparable performance and fairness relative to other debiasing methods and exhibits a reduced fairness gap compared to the baseline model in most scenarios. Specifically, Fig. 2 reveals that the proposed model's performance in Edema identification is on par with other debiasing methods across five evaluation metrics and shows a smaller fairness gap than the baseline in most demographic categories. The results for the other nine radiological labels are shown in Supplementary Figures E1–E9. Fig. 3 highlights that the proposed model has a lower fairness gap than the baseline in all categories, except for sex when assessed with ECE. In terms of overall model performance, the proposed model also matches other debiasing methods. It is important to note that no single method consistently outperforms the others across all metrics and demographic groups, reflecting the inherent challenges in mitigating bias within DL models.

**Fig. 4:**
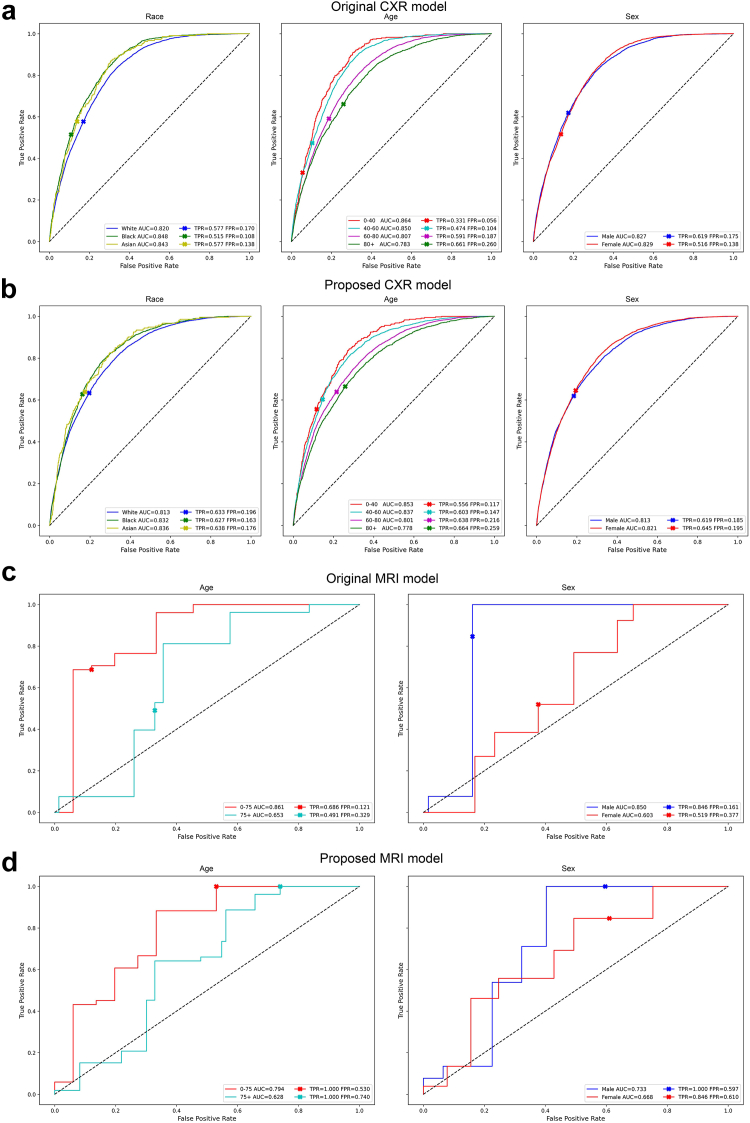
ROC curve of the detection results using the baseline model and the model trained on augmented images. (a) and (b) The detection of Edema from CXR in different race, age, and sex groups. (c) and (d) The detection of Alzheimer's disease from brain MRI in different age and sex groups. The TPR and FPR at the cutoff points are shown in each plot.

As shown in Fig. 4, the AUC values of the model trained with proposed augmented images do not decrease substantially in edema and AD detections, and the disparities for TPR and FPR are smaller than those of the original model. The results of all ten radiological findings for CXR images are shown in Supplementary Table C1.

As shown in Tables 6 and 7, our proposed augmentation scheme could also apply to testing data (test-time augmentation) without re-training the model (Second row). By incorporating the augmentation scheme in either the training or testing phases, the model could achieve the lowest disparities in AUC, ECE, and error rate across different age groups and in all metrics across different race or sex groups. Similarly, the model trained or tested using the proposed augmented MRI achieved the lowest disparities in all metrics across different age groups and in metrics except ECE in the sex groups. Furthermore, when adding the augmentation scheme to the existing debias methods for CXR or MRI, the disparity decreased (Supplementary Tables D1–D5). The results of using ResNet 50 architecture, using CheXpert dataset, and without ImageNet pretrained weights are presented in Supplementary Tables D6–D8, respectively.

**Table 6:** Results of macro average disparities among race, age, and sex in each evaluation metric for 10 radiological finding detection using CXR.

Train-time aug.	Test-time aug.	Race disparity				
		AUC	BCE	ECE	Error rate	Precision
w/o	w/o	0.040 [−0.020 to 0.099]	0.063 [−0.018 to 0.144]	0.015 [−0.012 to 0.042]	0.055 [0.010–0.100]	0.044 [−0.033 to 0.120]
w/o	w/	0.037 [−0.009 to 0.084]	0.057 [−0.013 to 0.128]	**0.013 [−0.007 to 0.032]**	**0.047 [−0.002 to 0.096]**	0.060 [−0.020 to 0.140]
w/	w/o	**0.035 [**−**0.016 to 0.086]**	0.058 [−0.019 to 0.134]	0.018 [−0.003 to 0.039]	0.052 [0.007–0.097]	**0.040 [**−**0.018 to 0.099]**
w/	w/	0.037 [−0.016 to 0.090]	**0.056 [**−**0.014 to 0.126]**	0.014 [−0.001 to 0.028]	0.052 [0.003–0.101]	0.045 [0.004–0.087]
		**Age disparity**				
w/o	w/o	0.114 [0.012–0.215]	**0.183 [**−**0.104 to 0.470]**	0.031 [−0.019 to 0.081]	0.208 [−0.060 to 0.476]	**0.093 [**−**0.060 to 0.246]**
w/o	w/	**0.106 [0.013–0.200**]	0.189 [−0.098 to 0.476]	0.036 [−0.021 to 0.093]	0.181 [−0.112 to 0.473]	0.120 [−0.062 to 0.302]
w/	w/o	0.125 [0.041–0.209]	0.197 [−0.108 to 0.502]	**0.023 [**−**0.032 to 0.078]**	0.179 [−0.040 to 0.397]	0.099 [−0.091 to 0.289]
w/	w/	0.112 [0.012–0.212]	0.186 [−0.086 to 0.457]	0.026 [−0.026 to 0.077]	**0.163 [**−**0.110 to 0.437]**	0.104 [−0.066 to 0.274]
		**Sex disparity**				
w/o	w/o	0.010 [−0.009 to 0.030]	0.025 [−0.019 to 0.069]	0.013 [−0.014 to 0.039]	0.027 [−0.009 to 0.063]	0.020 [−0.017 to 0.057]
w/o	w/	**0.010 [−0.008 to 0.029]**	**0.020 [−0.014 to 0.054]**	0.009 [−0.006 to 0.023]	0.023 [−0.015 to 0.061]	0.035 [−0.041 to 0.110]
w/	w/o	0.014 [−0.014 to 0.042]	0.021 [−0.009 to 0.051]	**0.007 [−0.005 to 0.019]**	0.021 [−0.014 to 0.055]	**0.016 [**−**0.012 to 0.045]**
w/	w/	0.013 [−0.011 to 0.037]	0.021 [−0.012 to 0.054]	0.007 [−0.007 to 0.021]	**0.015 [−0.014 to 0.044]**	0.026 [−0.020 to 0.072]

The high disparities of the model indicate inequitable predictions. The minimum values are highlighted in bold.

**Table 7:** Results of disparities among age and sex in each evaluation metric for AD detection using brain MRI.

Train-time aug.	Test-time aug.	Age disparity				
		AUC	BCE	ECE	Error rate	Precision
w/o	w/o	0.209	0.322	0.109	0.273	0.428
w/o	w/	0.251	0.109	0.024	0.173	0.226
w/	w/o	**0.163**	0.253	0.070	**0.128**	**0.095**
w/	w/	0.170	**0.038**	**0.012**	0.151	0.107
		**Sex disparity**				
w/o	w/o	0.247	0.369	**0.033**	0.258	0.373
w/o	w/	0.138	**0.038**	0.062	**0.124**	0.182
w/	w/o	0.065	0.076	0.042	0.178	0.156
w/	w/	**0.064**	0.055	0.100	0.168	**0.144**

The high disparities of the model indicate inequitable predictions. The minimum values are highlighted in bold.

### Experiment D: task transfer from image label detection to demographic attribute identification

**Fig. 5:**
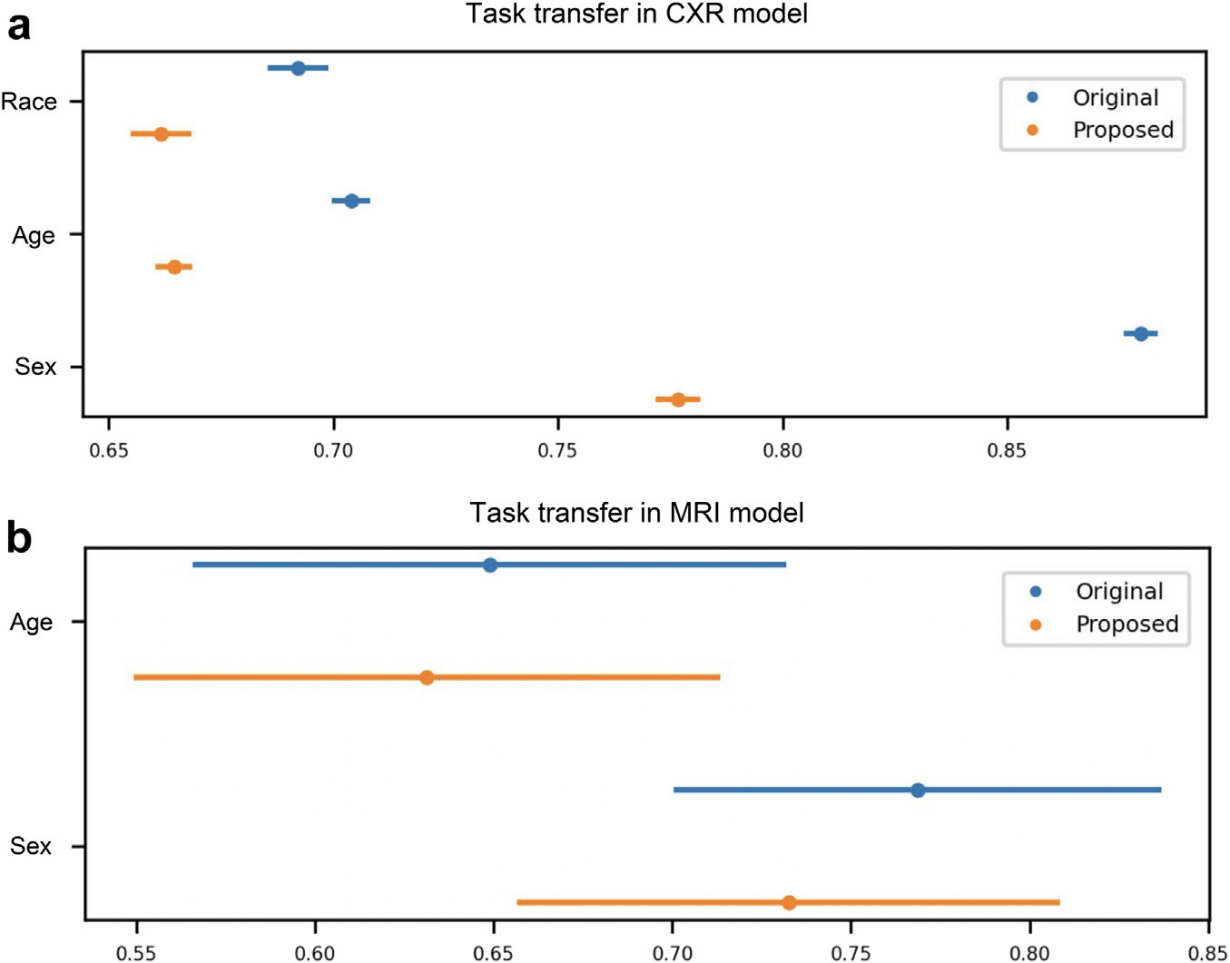
Results of task transfer from radiological/AD label detection to race/age/sex detection. (a) The results of the CXR task. (b) The results of the brain MRI task. The confidence interval for both figures is calculated from 1000 bootstrap iterations.

Figs. 5a and 4b present the result obtained in detecting demographic attributes using image features embedded in models trained for the detection of radiological findings and AD, respectively. The lower AUCs of the model trained with augmented images indicate that the model embedded less demographic information. Supplementary Table F1 shows the additional results in the task transfer experiment using ResNet50 architecture and the CheXpert dataset, where similar results were obtained.

### Model interpretation

**Fig. 6:**
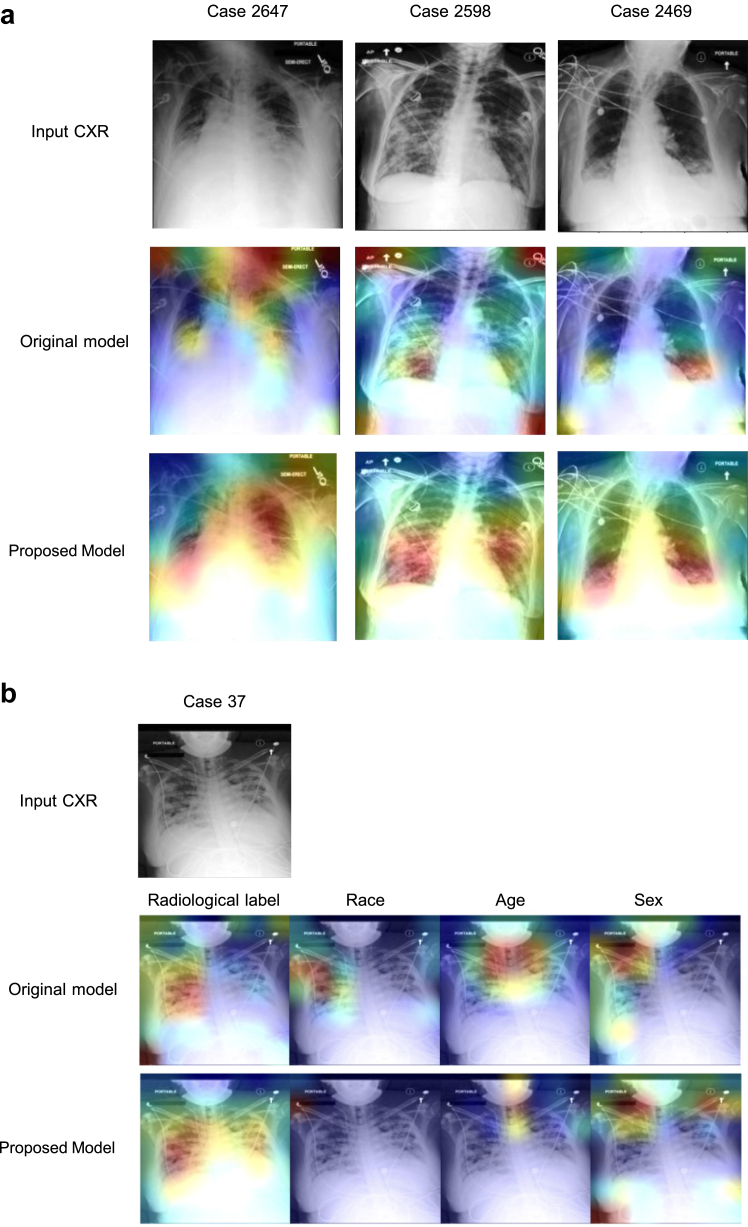
Model visualisation based on single cases. (a) Heatmaps of the original model and the model trained using the augmented data. The original model includes cues outside the lungs or even no cues and the model trained using the augmented data shifted the focus to cues within the lungs where the findings are supposed to be. The CXRs are the example of ‘Consolidation’ patients. (b) The heatmaps of a representative case generated by the original model and proposed model for predicting radiological label, race, age, and sex. The original model shows similar distributions across different tasks.

**Fig. 7:**
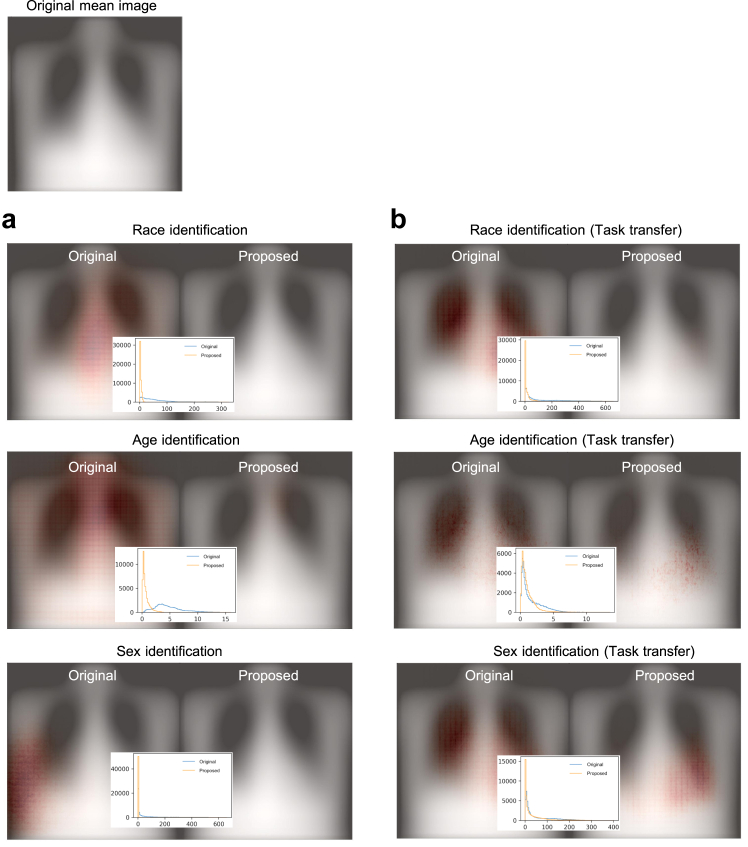
Saliency maps and the gradient distribution of the original model and the model trained using the augmented data. (a) The maps and distribution for race, age, or sex identification. (b) The maps and distribution in the task transfer experiment, where the model trained for radiological label detections was transferred for race, age, or sex identification.

Fig. 6a presents an example of heatmaps generated from the original model and the model trained using the augmented data using GradCAM. The results using other interpretation methods are shown in Supplementary Figure G11. In these examples, the original model was unable to locate cues related to ‘Consolidation’, whereas the model trained using the augmented data was able to locate the abnormality. Fig. 6b displays the heatmaps of a representative case generated by the original model for predicting radiological label, race, age, and sex, which exhibit similar distributions. In contrast, the proposed model yields different distributions. Fig. 7a and b depict the mean saliency maps obtained from the original model and the model trained using augmented data, accompanied by the corresponding distributions of gradients in Experiment B and Experiment D, respectively. The saliency maps illustrate that the original model relied heavily on certain regions in the CXRs to make decisions, while these regions were no longer prioritised in the proposed model. This suggests that the model could be previously using specific demographic features as shortcuts, and the augmentation process helped the model overcome these biases. Supplementary Figures G1–G10 show the saliency maps of models used for detecting radiological findings.

## Discussion

Shortcut learning refers to a phenomenon in which a DL model memorises specific features or patterns (i.e., simple solutions) in the training data instead of learning the underlying relationships between the input and the target.^63^ Our objective in this study was to identify if DL models were embedding demographic shortcuts in detecting of image labels and then evaluate if augmenting the training data could combat these shortcuts, thereby improving fairness. As demonstrated by the high AUCs of the original model in Experiment B, it is far easier for a DL model to detect demographic attributes than to detect image labels (e.g., Race: 0.948 vs. Radiological findings: 0.744). Thus, including these demographic shortcuts undermines the ability of the model to perform the classification task appropriately, which it was designed for, and can seriously skew detection results for radiological findings or diseases.^25^^,^^64^ This study indicates that the proposed dataset augmentation scheme is effective in mitigating the impact of demographic features in medical images. Specifically, the use of augmented images leads to reduced performance disparities between demographic groups while maintaining the original detection performance. Compared to existing debiasing methods, our proposed augmentation scheme shows comparable or even superior effectiveness in reducing disparities between demographic groups, as shown in Fig. 2. In addition, our proposed augmentation scheme offers several advantages over other debiasing techniques. Firstly, it is model- and data-agnostic, meaning that it can be applied to various models and datasets. Secondly, our method does not require demographic labels during the training process. Thirdly, it does not affect the amount of data, as we do not require the generation of synthetic data or the removal of existing data to create a balanced dataset. Lastly, our method can be applied only during the testing time, making it particularly useful in situations where re-training the model is not feasible. To the best of our knowledge, this is the first study to introduce a dataset augmentation scheme to mediate the influence of demographic-related features in medical images.

Data augmentation is a technique that expands the size and diversity of a training dataset by creating new examples from the original data through various transformations, such as rotating or scaling. One possible reason why the augmentation process can alleviate shortcut learning is that it exposes the model to a more comprehensive range of features, patterns, and contexts by adding new examples with different variations. As a result, the model is less likely to recognise demographic information that depends on specific patterns. When recognizing demographic information becomes as challenging as identifying radiological findings or diseases (e.g., Race: 0.776 vs. Radiological findings: 0.724), the model is less likely to take demographic information as shortcuts. Our demonstration of lower gradients in the mean saliency maps indicates that the proposed augmented data made it more challenging for models to capture demographic-related features. This may explain why the augmentation scheme is effective in reducing disparities across different groups.

The above-mentioned concept was supported by our four experiments. The results of the chi-square test and permutation test from Experiment A revealed that the radiological findings were strongly related to demographics. In Experiment B, we utilised the proposed augmentation scheme for retraining the models, which led to poor performance in detecting demographic attributes (Tables 4 and 5). In instances where demographic characteristics are not readily discernible by DL models, our assumption is that the DL model designated for pathology detection cannot utilise these shortcuts. Consequently, this could lead to a reduction in the disparity of performance between different demographic groups, as demonstrated in Experiment C. We also demonstrate that the proposed model maintains a reasonable level of performance in detecting radiological findings or AD. The results of the task transfer test from Experiment D revealed that the distorted images embedded less demographic information from images, which means that these images could be used as training data to prevent the model from taking demographic shortcuts in the detection of radiological findings.

Ensuring fairness when using DL models for diagnosis and prognostic analysis requires that practitioners understand the means by which DL models formulate their decisions.^65^

However, interpreting the model's operations presents a significant challenge. The complexity arises from the intricate algorithms and the model's non-transparent decision-making process.^66^ Some DL applications (e.g., classifying handwritten digits) can be elucidated from a purely visual perspective^52^; however, saliency maps are notoriously unreliable due to a lack of reproducibility and sensitivity in modelling parameters and difficult data distributions.^67^, ^68^, ^69^ The extreme complexity of radiographic images renders many of the explanations provided by machines opaque to human comprehension. This could make eliminating shortcuts a serious ongoing challenge since we could not understand how models exploit demographic features by visualised saliency maps. It is challenging for the current model explanation methods to fully uncover how the deep learning model operates. The saliency maps and Grad-CAM heatmaps suffered from limitations such as inconsistency and hard to interpret.^70^ The transfer task experiment is a method for investigating the extent to which features extracted by DL models are dependent on the tasks being performed, such as radiological diagnoses and race detection. However, when assessing the degree to which demographic information is utilised for making predictions, it is important to perform additional tests such as test set resampling, as indicated in a prior study.^46^

We compared our augmentation scheme with existing approaches as shown in Table 3. Most of the existing approaches are supervised learning procedures that require demographic labels; however, that kind of information is not always available. Another study used a transfer learning approach to prevent the model from exploiting demographic-related features as shortcuts.^71^ However, additional tasks were required in the transfer-based training and it relies on an assumption that the features learned in one diagnostic task are related to the other diagnostic task.^71^ Zhang and colleagues previously observed that the majority of debiasing methods operate during the training phase.^23^ To overcome this limitation, we opted to implement dataset augmentation as an unsupervised approach. This method offers greater generalizability and is particularly useful in cases where model retraining is not feasible, as it can be directly applied to test data. However, similar to many debiasing methods, efforts to bridge the fairness gap frequently result in diminished model performance.^72^ The observed higher performance, which may be biased, could be attributed to shortcuts prevalent in the privileged group. Balancing the maintenance of high performance while enhancing fairness remains a significant challenge.

Although our data augmentation reduced the algorithm's ability to predict demographic attributes from CXR and brain MRI images, it did not abolish it. Alarmingly, our model was better at predicting race than detecting the radiologic pathology it was trained for, both before and after image augmentation. This demonstrates the need for further research into limiting the ability of algorithms to learn demographic data that might be used to make decisions instead of clinical features. Without deliberately making sure sensitive attributes such as race, age, or other demographic information are not used for prediction, classification, or optimization, the data science community risks the perpetuation of health disparities from implicit bias in clinical decision-making that currently exists. Moreso, given the black box nature of DL models, it is virtually impossible to determine whether a prediction or classification is based on proxies of race or the relevant clinical features. Making sure a DL model does not learn demographic information that should not be used as an input feature (e.g., race-ethnicity of a prisoner by an algorithm that informs the decision by a judicial court to grant parole) is one strategy to prevent algorithms from having the same implicit biases as humans. Another strategy is explicitly using demographic information to reweight features to provide an output that corrects the implicit human bias. This is an area of research that has not been fully explored.

### Limitation

This study used MIMIC-IV to obtain self-reported labels of race, which may have been influenced by criteria used in the assignment of racial characteristics.^61^ The process of CXR or MRI labelling relied on manual diagnosis by radiologists or neurologists, which may have been affected by the sex of the patients or variations in the healthcare system.^14^ When supervised training is employed, the patterns learned by the model can be affected by device specifications, the use of tokens, or biassed annotation, resulting in inequitable predictions.^61^ In other words, the data collection and cleaning process can irreversibly bias the data.

Furthermore, the MIMIC-CXR dataset displayed substantial imbalances in sample sizes across racial groups (e.g., 75.8% White compared to 4.3% Asian), potentially skewing the ECE metric. Such an imbalance may lead to observed disparities in ECE that are more indicative of the metric's inherent biases rather than actual calibration inaccuracies within the models.^73^^,^^74^

### Conclusion

To conclude, our study demonstrated that DL models can exploit demographic features in medical images as shortcuts in the detection of image labels. We also demonstrated that the inclusion of such features could result in performance disparities among demographic groups. We developed an image augmentation scheme for training and testing in order to disguise the demographic information in CXRs and brain MRIs to improve the detection of radiological findings and disease. Ensuring accurate predictions made on the desired pathology while limiting algorithm bias and improving fairness has wide implications for generalizability and the eventual use of DL in healthcare applications. We strongly encourage the future development of tools to mitigate AI model demographic learning to prevent perpetuating existing racial disparities in medicine that would be otherwise unseen by the humans receiving the predictions.

## Contributors

All authors conceived and planned the study. R.W. implemented the models and did the results analysis. R.W., P.C.K., L.C.C., and K.P.S. drafted the manuscript. P.C.K. was responsible for the study design, results analysis, and manuscript writing. All the authors revised, reviewed, and approved the manuscript. All authors have directly accessed and verified the data. All authors were responsible for the decision to submit the manuscript.

## Data sharing statement

The CXR images used in this study are publicly available on MIMIC-CXR (https://physionet.org/content/mimic-cxr/2.0.0/) and CheXpert (https://stanfordmlgroup.github.io/competitions/chexpert/).

The brain MRI data used in this study are available on ADNI (https://adni.loni.usc.edu/data-samples/access-data/). The codes are available at https://github.com/Ryan-RE-Wang/Image-augmentation-improves-fairness-of-deep-neural-network-for-medical-image-interpretation.

## Declaration of interests

L.A.C. reports consulting fees from Philips, payment or honoraria for lectures, presentations, speakers bureaus from Stanford University, University of California San Francisco, University of Toronto (visiting professor), and Taipei Medical University. L.A.C. reports support for attending meetings from Australia New Zealand College of Intensive Care Medicine, University of Bergen, University Medical Center Amsterdam, Académie Nationale de Médecine, Doris Duke Foundation. L.A.C. reports leadership or fiduciary role in other boards, society, committee or advocacy group, paid or unpaid from PLOS Digital Health and Lancet Digital Health. L.A.C. reports cloud compute credits from Oracle. R.W., P.C.K, L.C.C., and K.P.S, have nothing to declare.
