# Supplementary material for: Drop the shortcuts: image augmentation improves fairness and decreases AI detection of race and other demographics from medical images
Source: eBioMedicine. 2024 Mar 11;102:105047. doi: 10.1016/j.ebiom.2024.105047 (PMC10945176; doi:10.1016/j.ebiom.2024.105047)
Supplement: Supplementary Tables [file mmc1.pdf]

# Supplementary

## A. Determination of transformation

We randomly applied the four augmentations to our images to train the model trained with augmentation, and the procedures of the augmentations were shown in Table A1.

Table A1. Augmentation example.

| Methods | Procedures |
|---|---|
| Rotating transformation (light) | Rotate images by a random amount ranging from -20° to 20°, -45° to 45°, or -90° to 90°. We then zoom out the images so that no information is lost. The rotating transform function is provided by scikit-image library, and the scaling function is provided by OpenCV library [1,2]. |
| (medium) | |
| (heavy) | |
| Shear transformation (light) | Apply shear transformation to images with a random radian ranging from -$\pi$/6 to $\pi$/6, from -$\pi$/5 to $\pi$/5, and from -$\pi$/4 to $\pi$/4. We then zoom out the images so that no information is lost. The shear transform function is provided by scikit-image library and the scaling function is provided by OpenCV library [1,2]. |
| (medium) | |
| (heavy) | |
| Scaling transformation (light) | Scale the side length of images to a randomly selected size ranging from 0·8 to 1, 0·6 to 1, or 0·4 to 1. The scaling function is provided by OpenCV library [2]. |
| (medium) | |
| (heavy) | |
| Fisheye distortion (light) | Apply fisheye effect with distortion coefficient set to 0·2, 0·3, or 0·4. We reference the fisheye transform function at this site (https://github.com/Gil-Mor/iFish). |
| (medium) | |
| (heavy) | |

Table A2 shows Examples of the four image distortion methods used in this study (one distortion per image) for brain MRI.

| Methods | Example |
|---|---|
| Rotating transformation (Angle: -10°) | 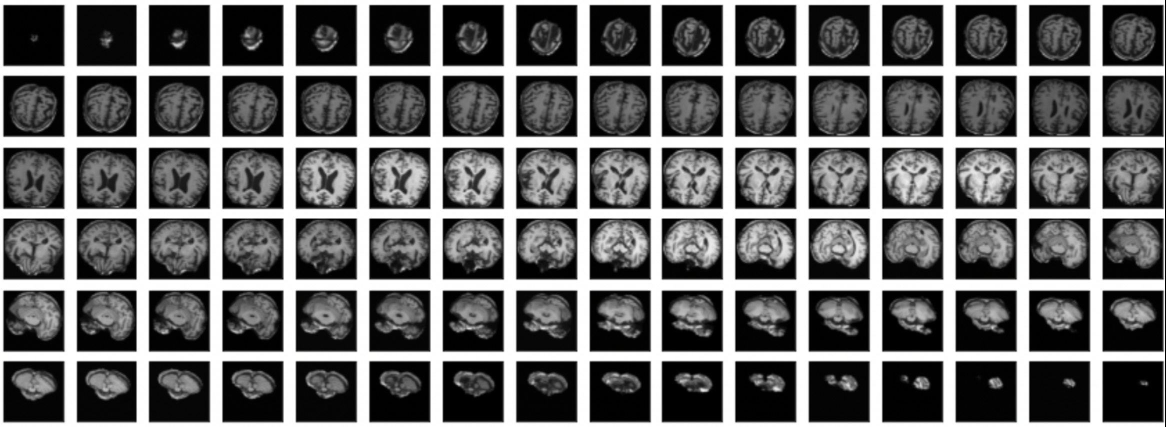 |
| Shear transformation (Radian: $\pi/8$) | 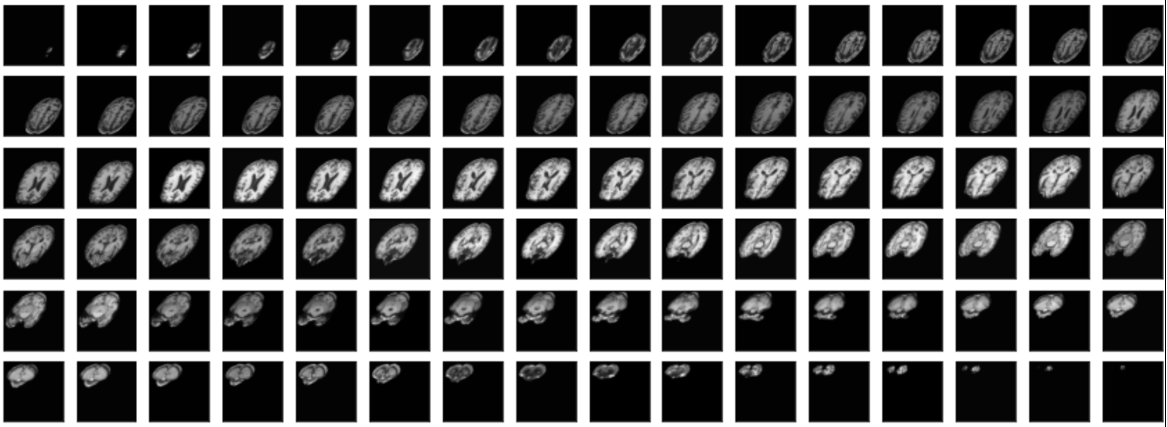 |
| Scaling transformation (Size: 0.95) | 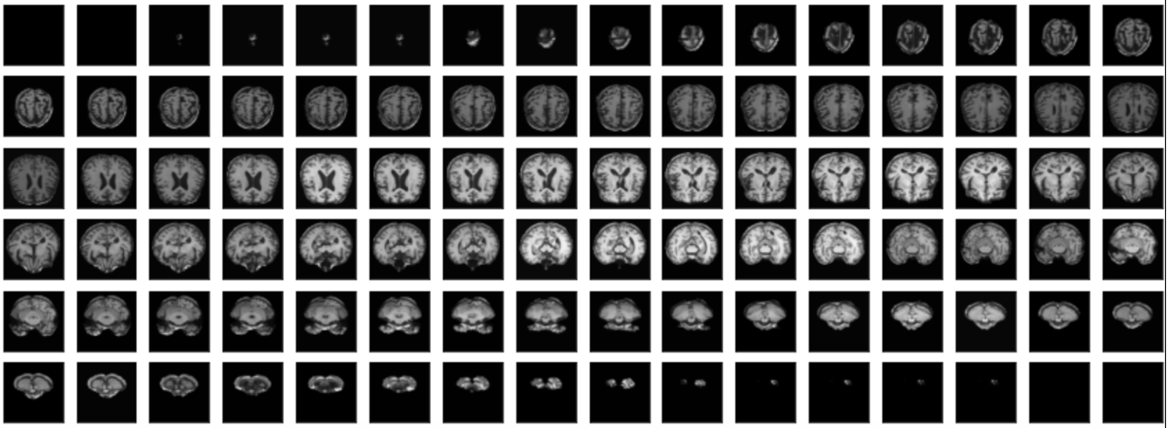 |
| Fisheye distortion | |

| | |
|---|---|
| (Central point: (94, 70, 170)) | 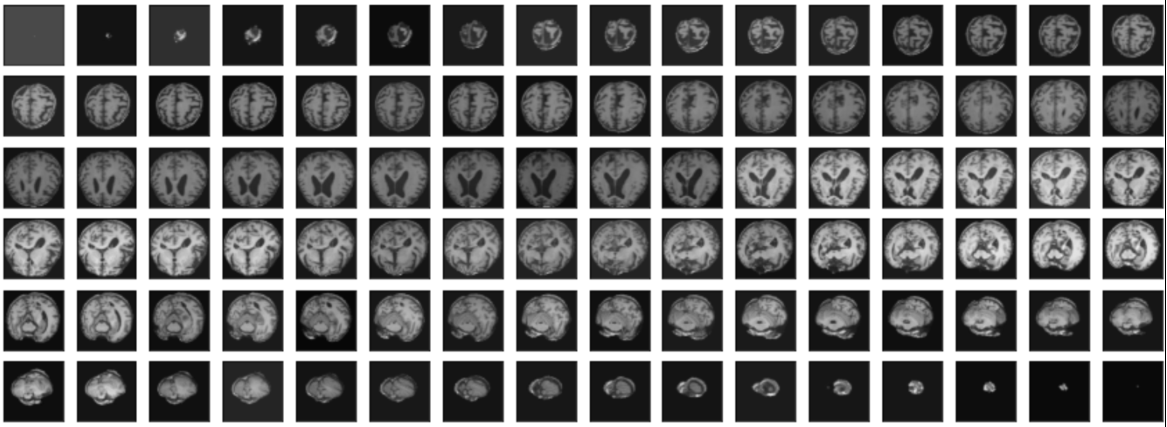 |

Table A3, Table A4, and Table A5 list the results for race, age, and sex prediction, respectively. Note that the rate of declination on the two tasks are calculated in the last two columns as $Declination\, rate = \frac{(\widehat{AUC} - AUC)}{AUC}$, where $\widehat{AUC}$ is the average AUC score of the proposed methods and $AUC$ is the average AUC score of the original model. All methods performed well in the detection of radiological features. The use of heavily rotated images for training had the most pronounced effect on mitigating the effects of race-related features, and the narrow CI for the detection of radiological features demonstrated that the training process was stable. The heavy extent for four augmentations could lower the performance of demographic attributes classification the most while maintaining the performance of radiological label detection. Hence, we randomly apply all four augmentations to a heavy extent to train and test our model trained with augmentation.

Table A3. Model performance in the race prediction.

| Method | AUC [±CI] | | | Declination rate (%) |
|---|---|---|---|---|
| | Asian | White | Black | |
| **w/o augmentation** | 0·943 [0·930-0·956] | 0·946 [0·936-0·956] | 0·954 [0·946-0·962] | N/A |
| **Rotating transformation** | | | | |
| -light | 0·915 [0·892-0·938] | 0·932 [0·924-0·940] | 0·940 [0·934-0·946] | -2·0 |
| -medium | 0·823 [0·710-0·936] | 0·844 [0·714-0·974] | 0·856 [0·728-0·985] | -11·2 |
| -heavy | 0·781 [0·682-0·881] | 0·816 [0·708-0·924] | 0·825 [0·714-0·935] | -14·8 |
| **Shear transformation** | | | | |
| -light | 0·860 [0·736-0·983] | 0·871 [0·738-1·004] | 0·881 [0·744-1·018] | -8·1 |
| -medium | 0·841 [0·704-0·978] | 0·862 [0·708-1·016] | 0·869 [0·715-1·024] | -9·5 |
| -heavy | 0·730 [0·556-0·904] | 0·771 [0·592-0·949] | 0·783 [0·601-0·966] | -19·7 |
| **Scaling transformation** | | | | |

| | | | | |
|---|---|---|---|---|
| -light | 0·934 [0·911-0·956] | 0·938 [0·923-0·954] | 0·948 [0·934-0·961] | -0·8 |
| -medium | 0·865 [0·717-1·014] | 0·879 [0·752-1·006] | 0·915 [0·854-0·975] | -6·5 |
| -heavy | 0·770 [0·555-0·984] | 0·806 [0·595-1·016] | 0·817 [0·606-1·029] | -15·8 |
| **Fisheye distortion** | | | | |
| -light | 0·919 [0·904-0·934] | 0·926 [0·918-0·935] | 0·939 [0·934-0·044] | -2·1 |
| -medium | 0·909 [0·902-0·916] | 0·916 [0·907-0·926] | 0·926 [0·916-0·936] | -3·2 |
| -heavy | 0·890 [0·859-0·920] | 0·907 [0·895-0·919] | 0·919 [0·911-0·928] | -4·5 |
| **Proposed augmentation** | **0·761** [0·624-0·898] | **0·779** [0·631-0·927] | **0·789** [0·635-0·943] | **-18·1** |

Table A4. Model performance in the age prediction.

| Method | AUC [±CI] | | | | Declination rate (%) |
|---|---|---|---|---|---|
| | 0-40 | 40-60 | 60-80 | 80- | |
| **w/o augmentation** | 0·964 [0·957-0·971] | 0·800 [0·723-0·877] | 0·753 [0·704-0·802] | 0·906 [0·900-0·912] | N/A |
| **Rotating transformation** | | | | | |
| -light | 0·954 [0·941-0·967] | 0·807 [0·770-844] | 0·764 [0·749-0·779] | 0·883 [0·859-0·907] | -0·4% |
| -medium | 0·949 [0·940-0·958] | 0·773 [0·693-0·853] | 0·751 [0·717-0·785] | 0·880 [0·870-0·890] | -2% |
| -heavy | 0·888 [0·738-1·038] | 0·767 [0·707-0·827] | 0·721 [0·682-0·760] | 0·821 [0·687-0·995] | -6·6% |
| **Shear transformation** | | | | | |
| -light | 0·954 [0·941-0·967] | 0·794 [0·759-0·829] | 0·742 [0·729-0·755] | 0·888 [0·872-0·904] | -1·3% |
| -medium | 0·941 [0·908-0·974] | 0·743 [0·638-0·848] | 0·735 [0·692-0·777] | 0·880 [0·858-0·901] | -3·6% |
| -heavy | 0·819 [0·489-1·149] | 0·765 [0·704-0·826] | 0·731 [0·691-0·772] | 0·859 [0·823-0·896] | -7·3% |
| **Scaling transformation** | | | | | |
| -light | 0·961 [0·956-0·966] | 0·814 [0·785-0·842] | 0·765 [0·731-0·798] | 0·900 [0·894-0·906] | +0·4% |
| -medium | 0·943 [0·914-0·972] | 0·776 [0·690-0·863] | 0·725 [0·646-0·804] | 0·880 [0·859-0·901] | -2·9% |

| | | | | | |
|---|---|---|---|---|---|
| -heavy | 0·855 [0·620-0·904] | 0·737 [0·561-0·913] | 0·690 [0·564-0·817] | 0·802 [0·603-1·001] | -9·7% |
| **Fisheye distortion** | | | | | |
| -light | 0·962 [0·959-0·965] | 0·774 [0·663-0·886] | 0·749 [0·708-0·789] | 0·871 [0·787-0·955] | -2% |
| -medium | 0·961 [0·959-0·963] | 0·813 [0·780- 0·846] | 0·756 [0·730-0·781] | 0·895 [0·892-0·899] | +0·1% |
| -heavy | 0·951 [0·937-0·965] | 0·737 [0·606-0·867] | 0·739 [0·703-0·775] | 0·883 [0·862-0·904] | -3·3% |
| **Proposed augmentation** | **0·884** [0·790-0·978] | **0·725** [0·617-0·833] | **0·693** [0·616-0·770] | **0·815** [0·716-0·914] | -8·9% |

Table A5. Model performance in the sex prediction.

| Method | AUC [±CI] | | Declination rate (%) |
|---|---|---|---|
| | Female | Male | |
| **w/o augmentation** | 0·995 [0·993-0·997] | 0·995 [0·993-0·997] | N/A |
| **Rotating transformation** | | | |
| -light | 0·992 [0·990-0·994] | 0·992 [0·990-0·994] | -0·3% |
| -medium | 0·976 [0·970-0·982] | 0·976 [0·970-0·982] | -1·9% |
| -heavy | 0·956 [0·918-0·994] | 0·956 [0·918-0·994] | -3·9% |
| **Shear transformation** | | | |
| -light | 0·993 [0·990-0·996] | 0·993 [0·990-0·996] | -0·2% |
| -medium | 0·988 [0·980-0·996] | 0·988 [0·980-0·996] | -0·7% |
| -heavy | 0·959 [0·883-1·035] | 0·959 [0·883-1·035] | -3·6% |
| **Scaling transformation** | | | |
| -light | 0·994 [0·990-0-·998] | 0·994 [0·990-0-·998] | -0·1% |
| -medium | 0·986 [0·980-0·992] | 0·986 [0·980-0·992] | -0·9% |

| | | | |
|---|---|---|---|
| -heavy | 0·884 [0·642-1·110] | 0·884 [0·642-1·110] | -11·6% |
| **Fisheye distortion** | | | |
| -light | 0·994 [0·992-0·996] | 0·994 [0·992-0·996] | -0·1% |
| -medium | 0·991 [0·986-0·996] | 0·991 [0·986-0·996] | -0·4% |
| -heavy | 0·989 [0·987-0·991] | 0·989 [0·986-0·992] | -0·6% |
| **Proposed augmentation** | **0·960** [0·931-0·989] | **0·960** [0·931-0·989] | **-2·8%** |

Tables A6, A7, and A8 showed the disparities in race, age, and sex using only rotation, shear, scaling, and fisheye as augmentation, respectively.

Table A6. Disparities in race using only a single augmentation method.

| Method | **AUC** | **BCE** | **ECE** | **Error rate** | **Precision** |
|---|---|---|---|---|---|
| Rotation | 0.033 [-0.017 - 0.084] | 0.067 [-0.015 - 0.148] | 0.017 [-0.005 - 0.040] | 0.045 [0.006 - 0.084] | 0.043 [-0.022 - 0.109] |
| Shear | 0.032 [-0.003 - 0.068] | 0.045 [0.006 - 0.084] | 0.022 [-0.014 - 0.059] | 0.034 [0.006 - 0.062] | 0.059 [-0.024 - 0.141] |
| Scaling | 0.033 [-0.009 - 0.074] | 0.058 [-0.012 - 0.128] | 0.016 [0.002 - 0.030] | 0.057 [0.017 - 0.097] | 0.043 [-0.018 - 0.104] |
| Fisheye | 0.034 [-0.003 - 0.071] | 0.058 [0.000 - 0.116] | 0.015 [0.000 - 0.031] | 0.049 [0.009 - 0.090] | 0.048 [-0.022 - 0.118] |

Table A7. Disparities in age using only single augmentation method.

.

| Method | **AUC** | **BCE** | **ECE** | **Error rate** | **Precision** |
|---|---|---|---|---|---|
| Rotation | 0.098 [0.010 - 0.185] | 0.172 [-0.119 - 0.464] | 0.034 [-0.023 - 0.091] | 0.168 [-0.011 - 0.347] | 0.098 [-0.095 - 0.292] |
| Shear | 0.130 [0.035 - 0.226] | 0.168 [-0.103 - 0.440] | 0.032 [-0.041 - 0.105] | 0.180 [-0.062 - 0.422] | 0.109 [-0.084 - 0.302] |
| Scaling | 0.121 [0.037 - 0.205] | 0.190 [-0.091 - 0.472] | 0.030 [-0.035 - 0.095] | 0.192 [-0.020 - 0.403] | 0.094 [-0.087 - 0.275] |
| Fisheye | 0.132 [0.032 - 0.233] | 0.157 [-0.080 - 0.394] | 0.042 [-0.030 - 0.113] | 0.189 [-0.013 - 0.390] | 0.102 [-0.128 - 0.332] |

Table A8. Disparities in sex using only a single augmentation method.

| Method | AUC | BCE | ECE | Error rate | Precision |
|---|---|---|---|---|---|
| Rotation | 0.015 [-0.011 - 0.042] | 0.028 [-0.015 - 0.070] | 0.008 [-0.005 - 0.022] | 0.018 [-0.010 - 0.047] | 0.015 [-0.019 - 0.049] |
| Shear | 0.008 [-0.005 - 0.021] | 0.016 [-0.019 - 0.051] | 0.011 [-0.016 - 0.038] | 0.020 [0.002 - 0.038] | 0.024 [-0.023 - 0.071] |
| Scaling | 0.010 [-0.008 - 0.028] | 0.021 [-0.010 - 0.052] | 0.006 [-0.005 - 0.018] | 0.022 [-0.006 - 0.049] | 0.016 [-0.021 - 0.054] |
| Fisheye | 0.014 [-0.017 - 0.045] | 0.022 [-0.012 - 0.055] | 0.005 [-0.005 - 0.016] | 0.027 [-0.017 - 0.070] | 0.017 [-0.022 - 0.056] |

## B. Chi-Square test results

Tables B1, B2, and B3 show the Chi-square test results of the demographic attributes and the image labels for MIMIC-CXR, CheXpert, and ADNI datasets, respectively.

Table B1. MIMIC-CXR dataset.

| | Race | Age | sex |
|---|---|---|---|
| **Atelectasis** | 2·22e-147 | 2·56e-263 | 8e-45 |
| **Cardiomegaly** | 1·92e-11 | 0 | 0·10 |
| **Consolidation** | 2·18e-17 | 2·95e-10 | 1·07e-12 |
| **Edema** | 4·06e-17 | 0 | 0·10 |
| **Enlarged Cardiomediastinum** | 3·47e-17 | 4·33e-15 | 4e-17 |
| **Lung Opacity** | 4·26e-46 | 6·35e-212 | 1·53e-25 |
| **No Finding** | 6·27e-273 | 0 | 2·53e-74 |
| **Pleural Effusion** | 0 | 0 | 3·24e-10 |
| **Pneumonia** | 7·56e-5 | 4·67e-15 | 1·84e-9 |
| **Pneumothorax** | 7·36e-93 | 7·64e-23 | 1·09e-35 |

Table B2. CheXpert dataset.

| | Race | Age | sex |
|---|---|---|---|
| **Atelectasis** | 1·42e-14 | 6·62e-43 | 4·83e-6 |
| **Cardiomegaly** | 3·13e-117 | 2·51e-225 | 3·59e-19 |

| | | | |
|---|---|---|---|
| **Consolidation** | 8·43e-5 | 5·71e-5 | 0·89 |
| **Edema** | 8·33e-18 | 1·01e-308 | 0·006 |
| **Enlarged Cardiomediastinum** | 0·54 | 0·28 | 5·59e-10 |
| **Lung Opacity** | 1·09e-7 | 1·13e-196 | 0·65 |
| **No Finding** | 2·48e-18 | 0 | 0·003 |
| **Pleural Effusion** | 8·94e-51 | 1·82e-294 | 0·20 |
| **Pneumonia** | 0·32 | 1·39e-13 | 1 |
| **Pneumothorax** | 2·05e-29 | 3·37e-197 | 0·012 |

Table B3. ADNI dataset.

| | **Age** | **sex** |
|---|---|---|
| **AD** | 0.417 | 0.062 |

## C. ROC curve for each radiological labels

Tables C1 and C2 show the ROC curves for each demographic group in all image labels for CXR and brain MRI images, respectively.

Table C1. ROC curve for CXR model.

<table>
<tr><td rowspan="2">Train-time aug.</td><td rowspan="2">Test-time aug.</td><td>Radiological label</td></tr>
<tr><td>Atelectasis</td></tr>
<tr><td>w/o</td><td>w/o</td><td>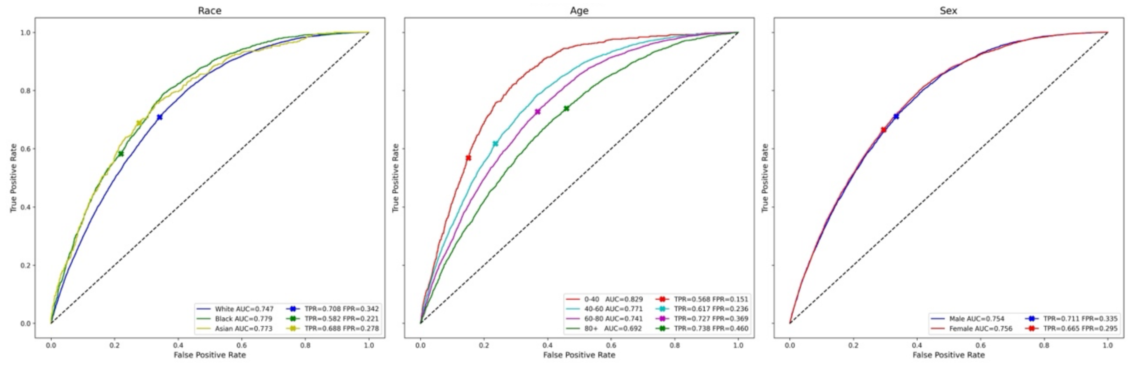
</td></tr>
</table>

<table>
<tr><td>w/o</td><td>w/</td><td>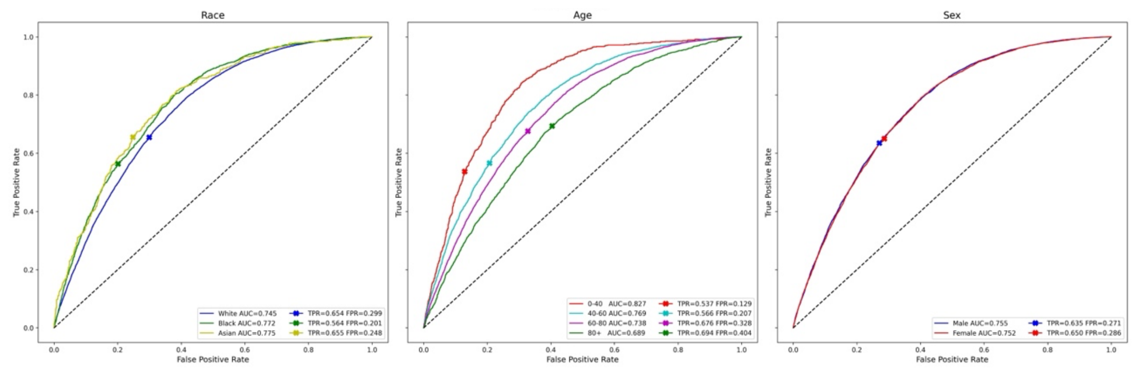
</td></tr>
<tr><td>w/</td><td>w/o</td><td>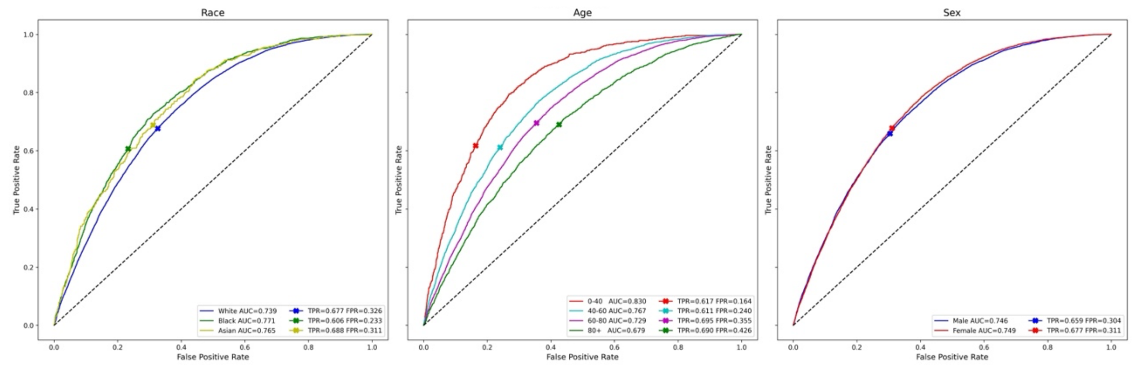
</td></tr>
<tr><td>w/</td><td>w/</td><td>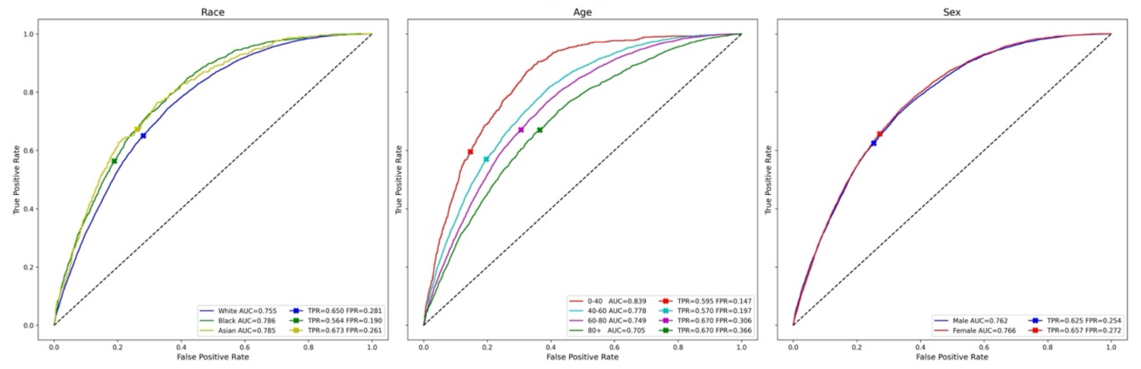
</td></tr>
<tr><td colspan="2"></td><td>Cardiomegaly</td></tr>
<tr><td>w/o</td><td>w/o</td><td>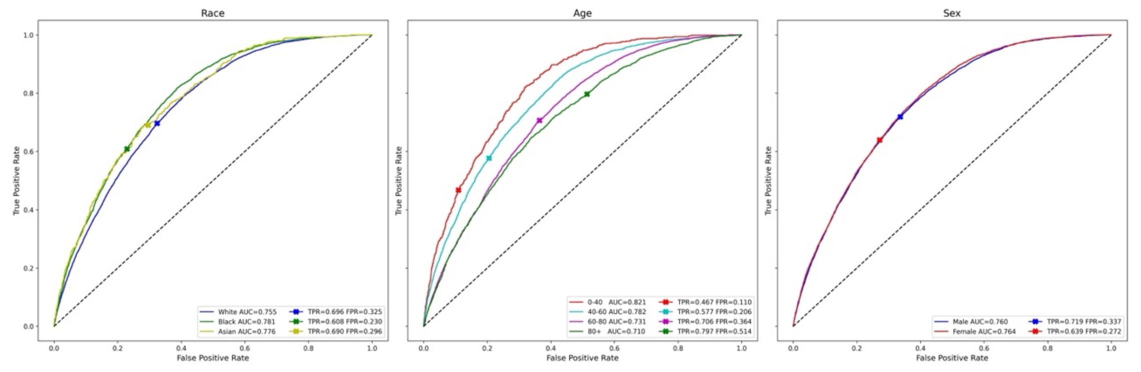
</td></tr>
</table>

| | | |
|---|---|---|
| w/o | w/ | 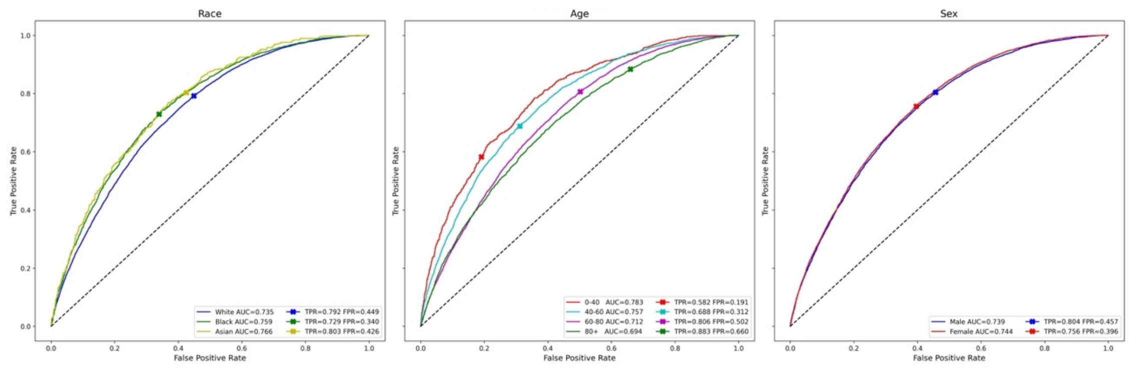 |
| w/ | w/o | 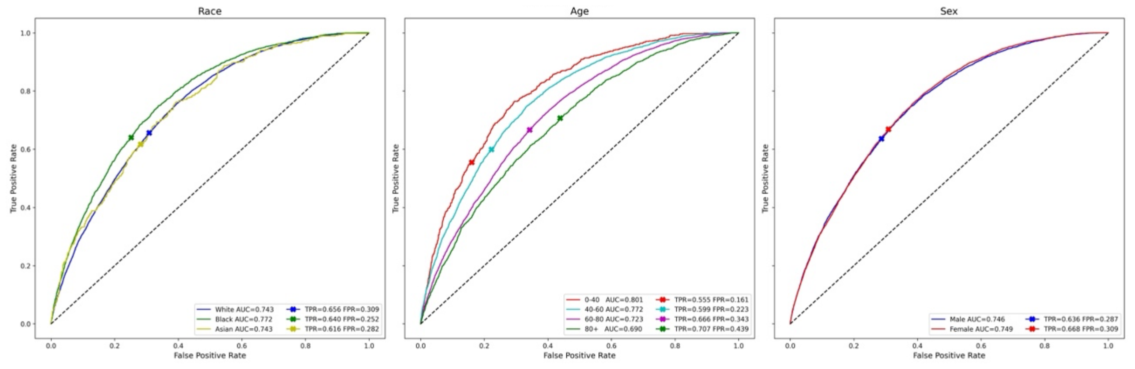 |
| w/ | w/ | 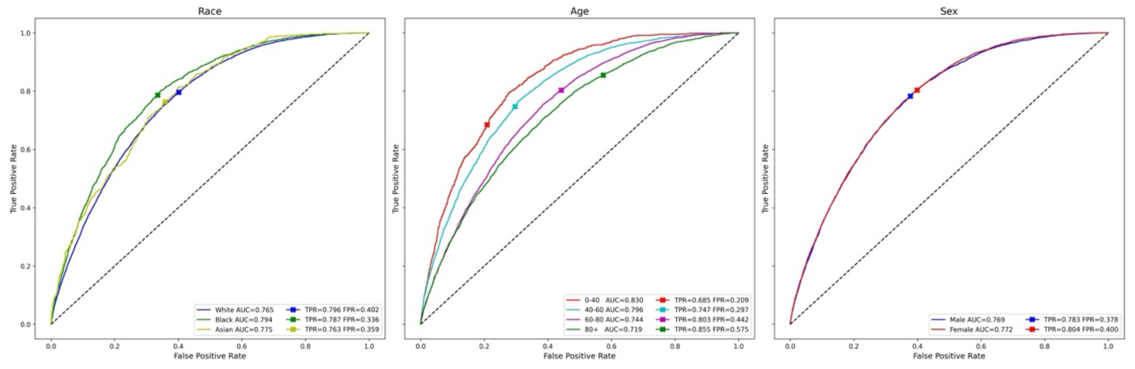 |
| | | Consolidation |
| w/o | w/o | 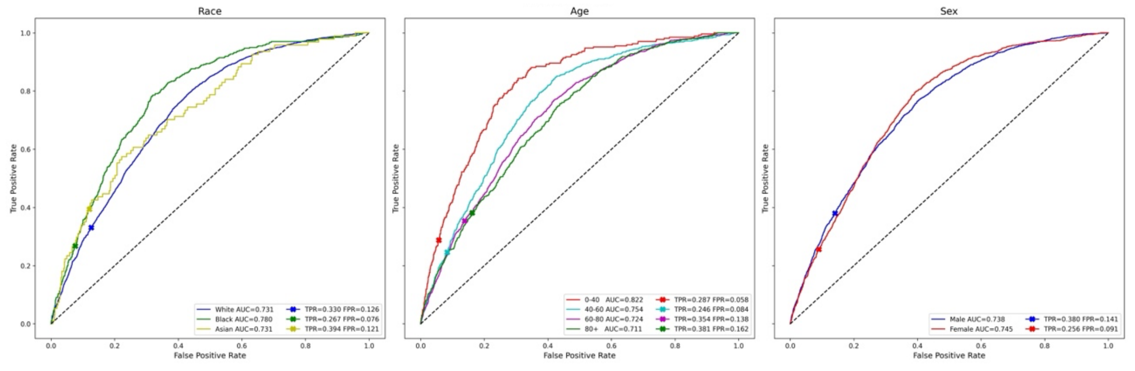 |

<table>
<tr><td>w/o</td><td>w/</td><td>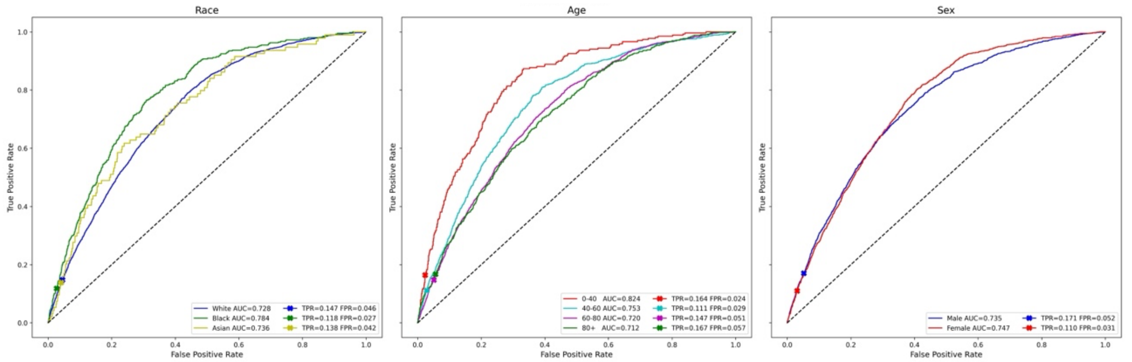
</td></tr>
<tr><td>w/</td><td>w/o</td><td>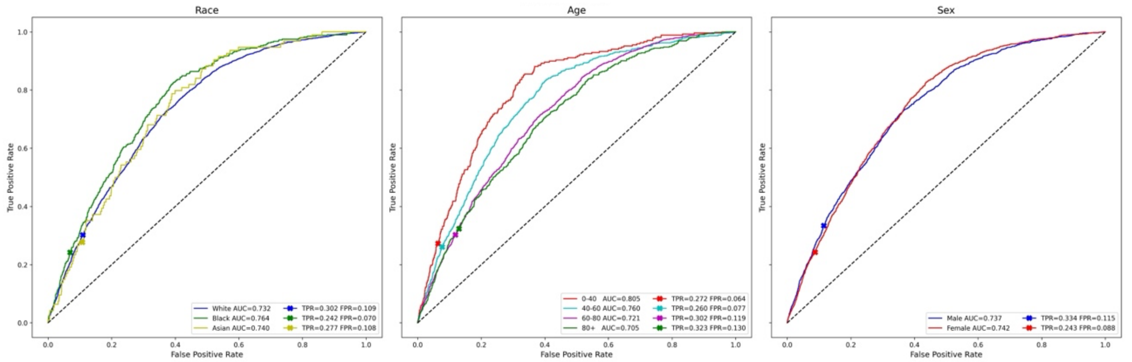
</td></tr>
<tr><td>w/</td><td>w/</td><td>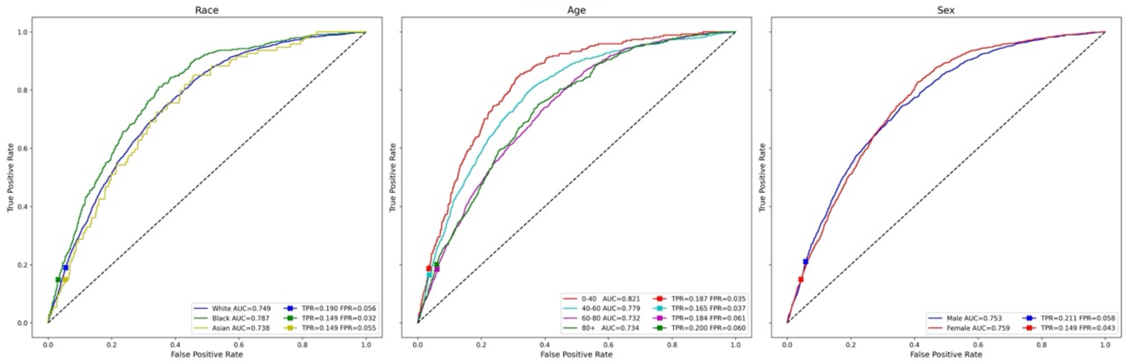
</td></tr>
<tr><td colspan="2"></td><td>Edema</td></tr>
<tr><td>w/o</td><td>w/o</td><td>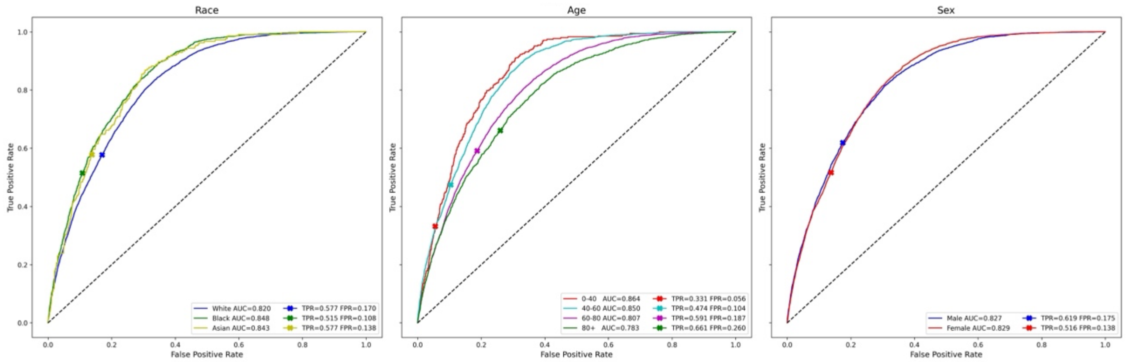
</td></tr>
</table>

| | | |
|---|---|---|
| w/o | w/ | 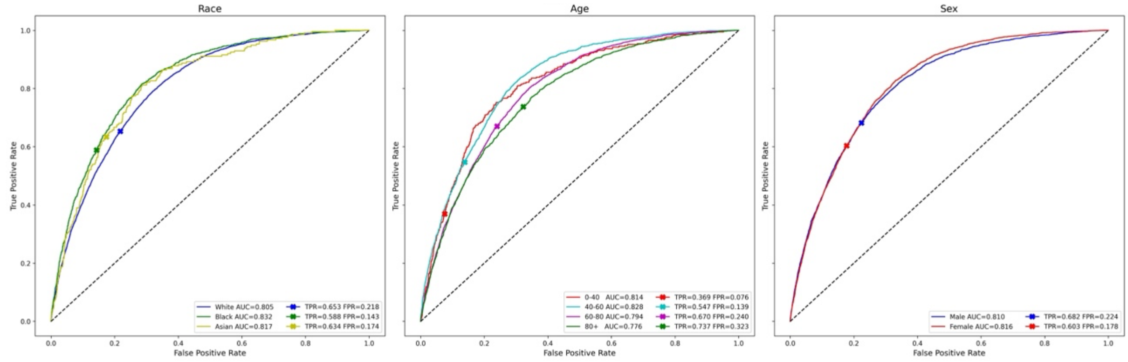 |
| w/ | w/o | 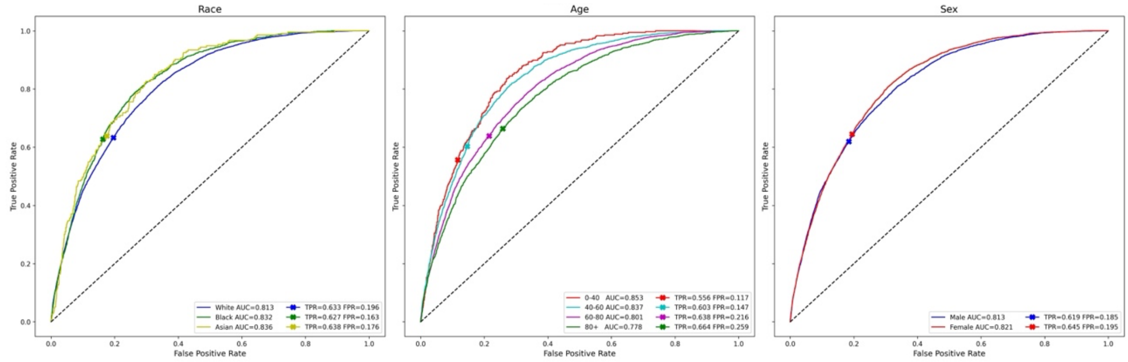 |
| w/ | w/ | 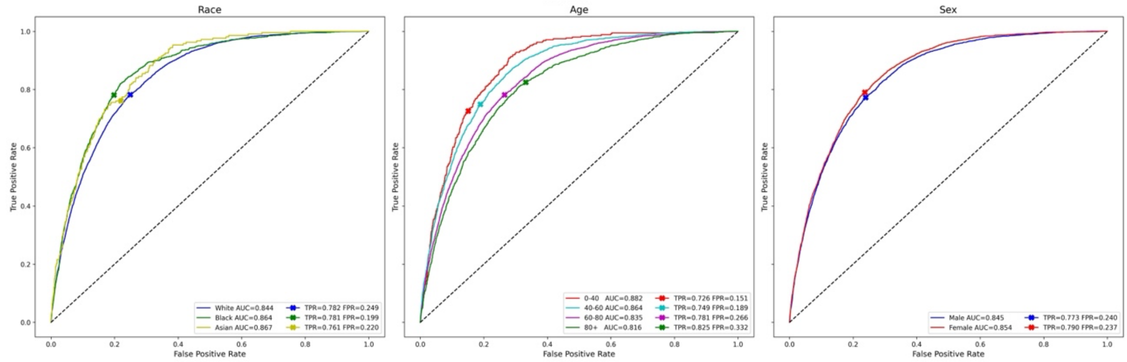 |
| | | Enlarged Cardiomediastinum |
| w/o | w/o | 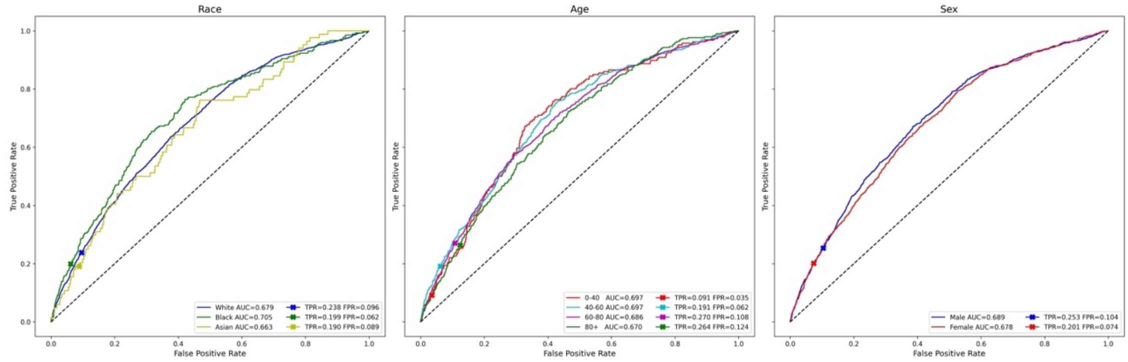 |

<table>
<tr><td>w/o</td><td>w/</td><td>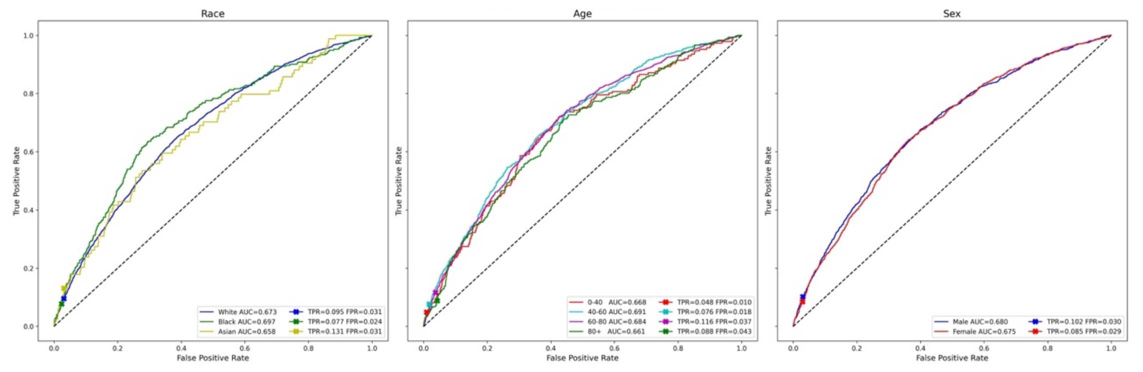
</td></tr>
<tr><td>w/</td><td>w/o</td><td>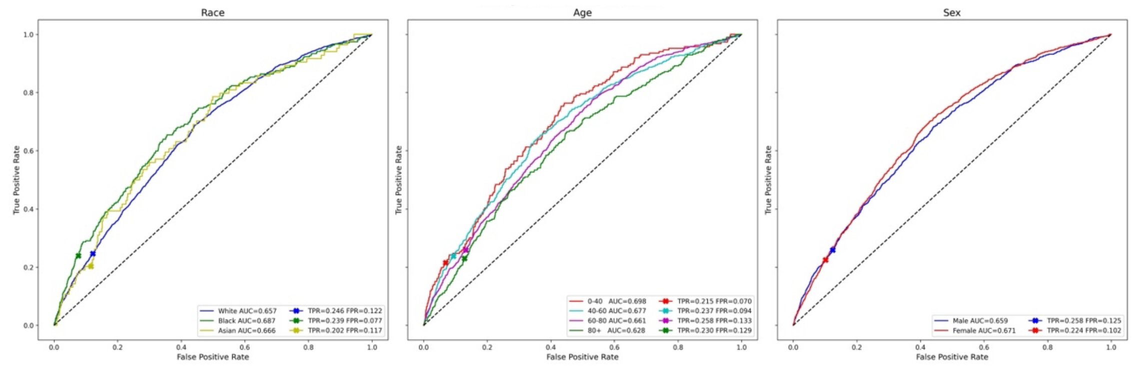
</td></tr>
<tr><td>w/</td><td>w/</td><td>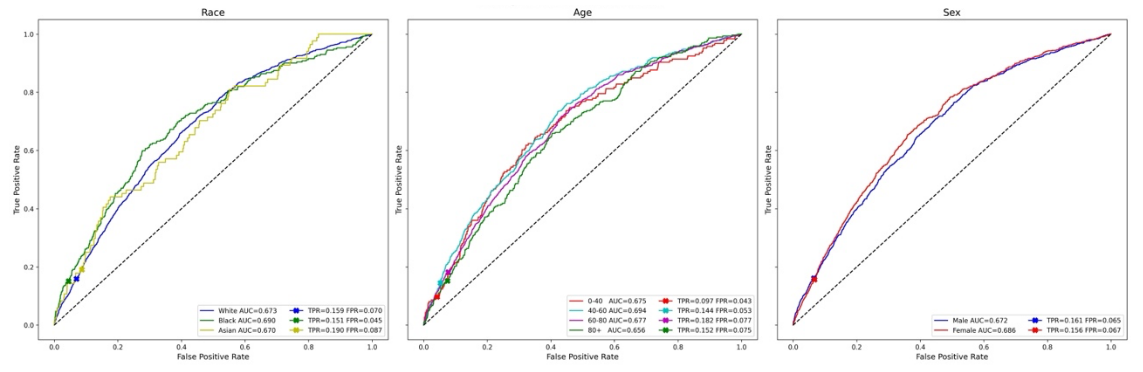
</td></tr>
<tr><td colspan="2"></td><td>Lung Opacity</td></tr>
<tr><td>w/o</td><td>w/o</td><td>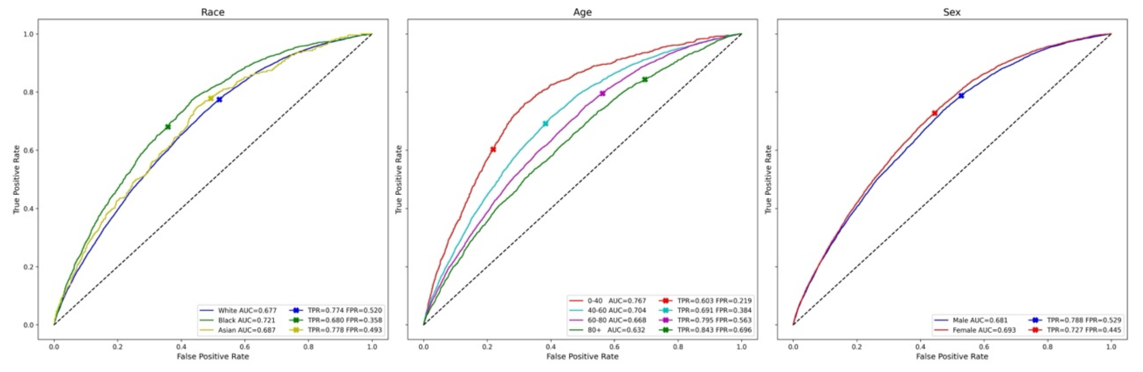
</td></tr>
</table>

<table>
<tr><td>w/o</td><td>w/</td><td>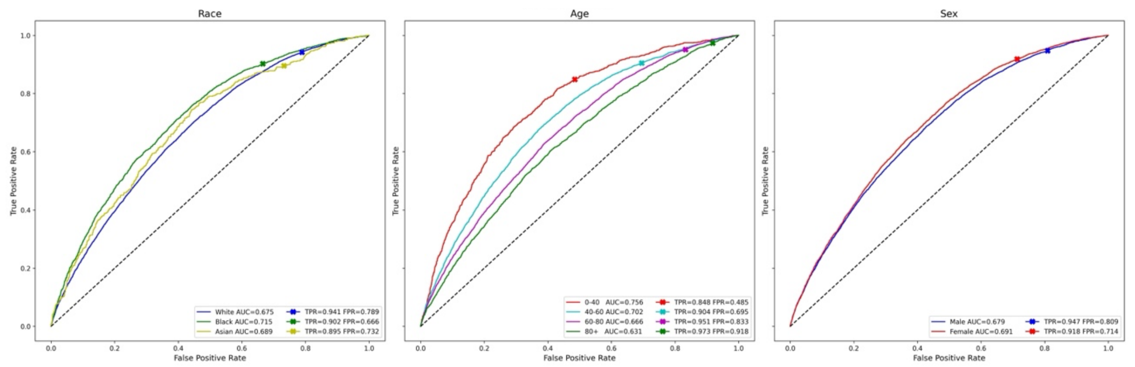
</td></tr>
<tr><td>w/</td><td>w/o</td><td>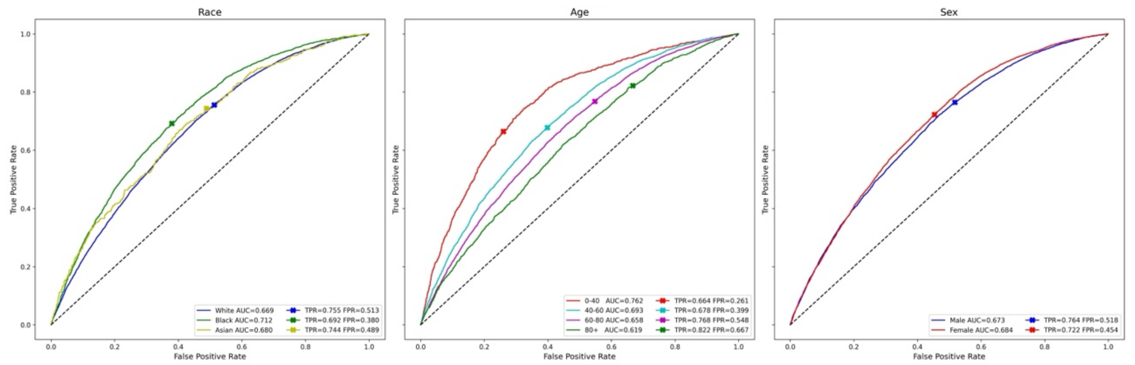
</td></tr>
<tr><td>w/</td><td>w/</td><td>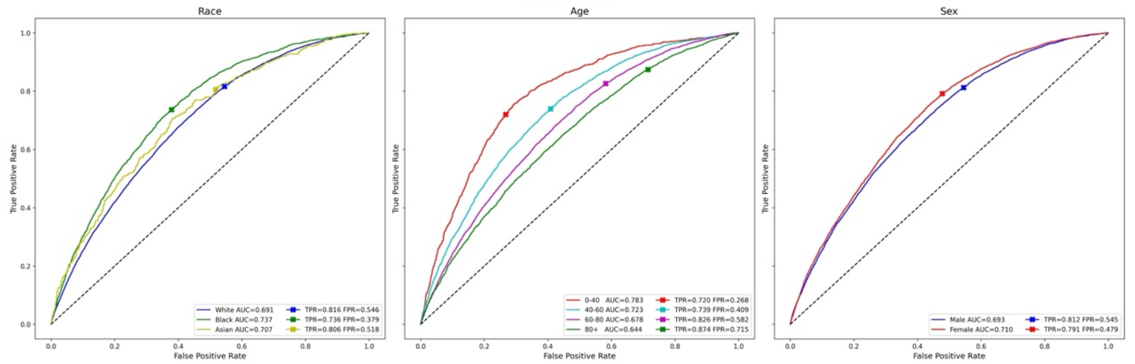
</td></tr>
<tr><td colspan="2"></td><td>No Finding</td></tr>
<tr><td>w/o</td><td>w/o</td><td>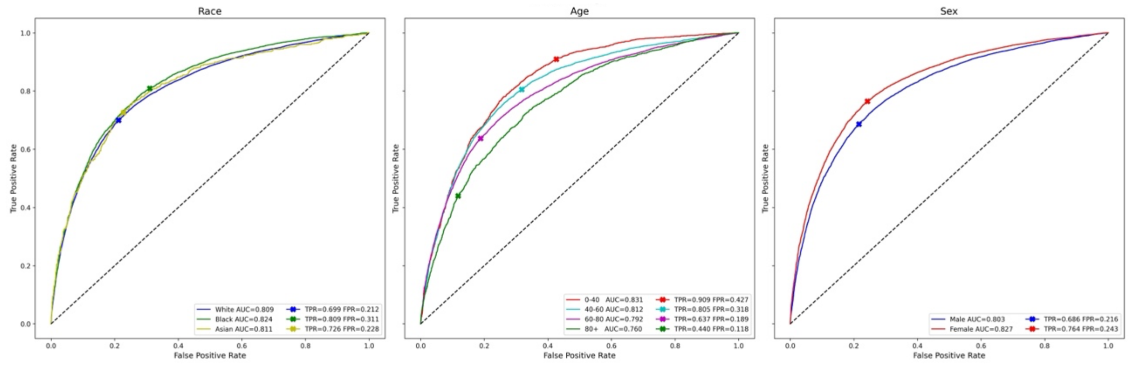
</td></tr>
</table>

| | | |
|---|---|---|
| w/o | w/ | 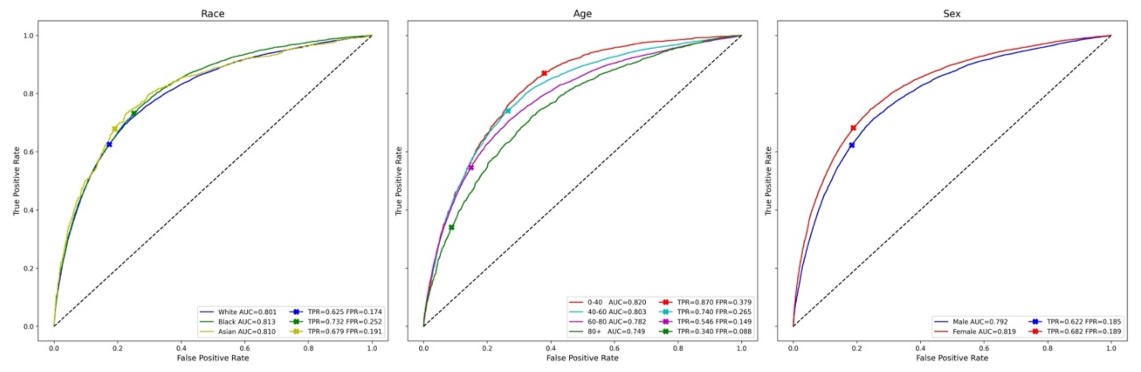 |
| w/ | w/o | 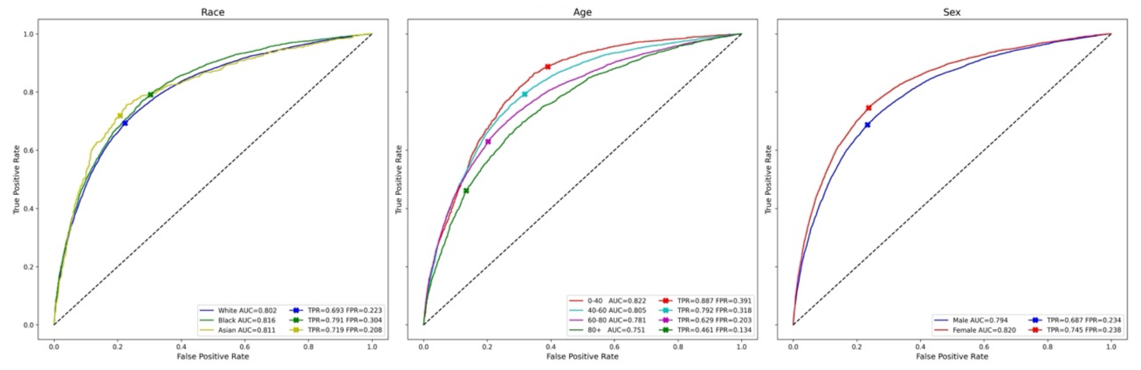 |
| w/ | w/ | 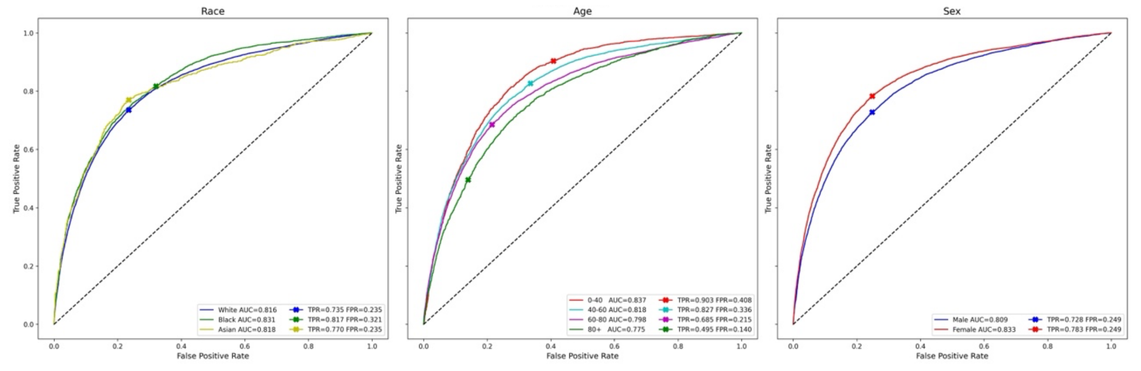 |
| | | Pleural Effusion |
| w/o | w/o | 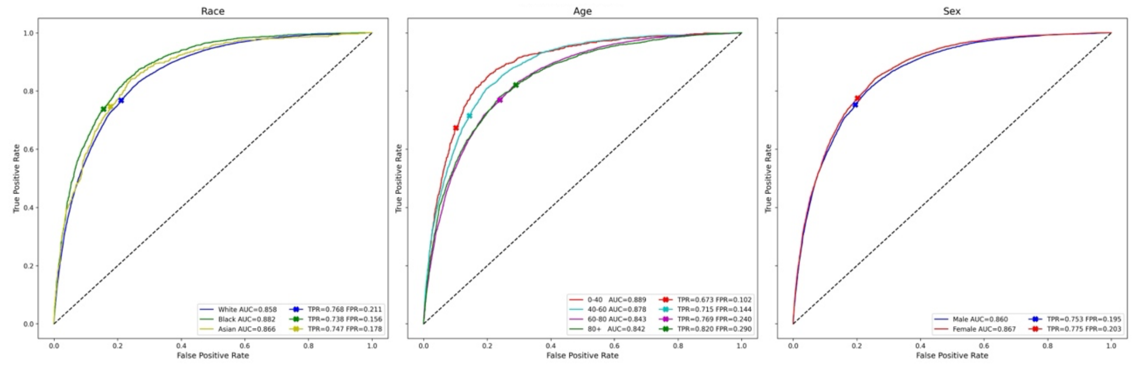 |

| | | |
|---|---|---|
| w/o | w/ | 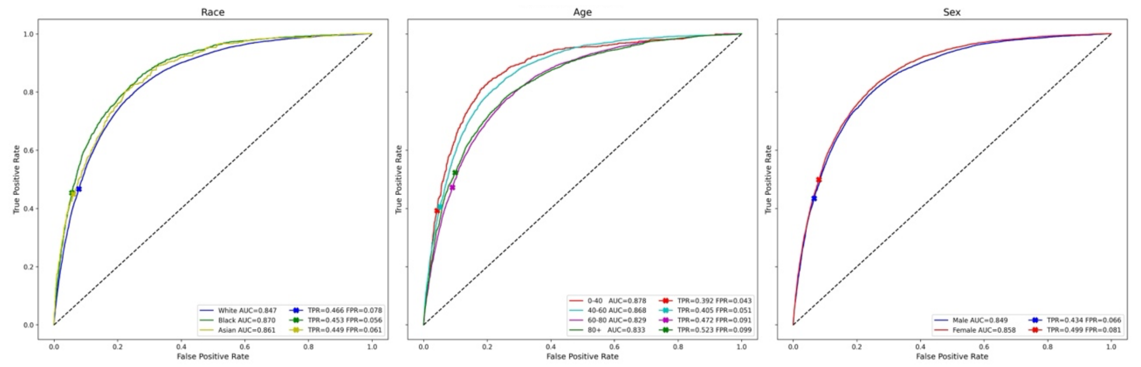 |
| w/ | w/o | 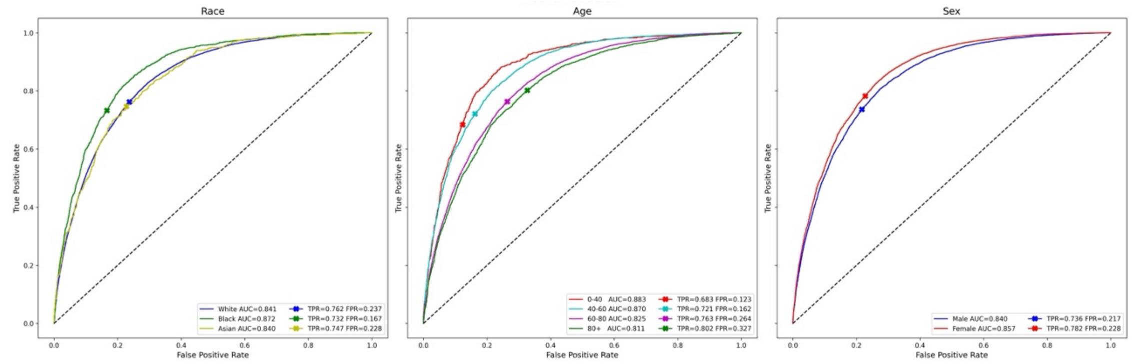 |
| w/ | w/ | 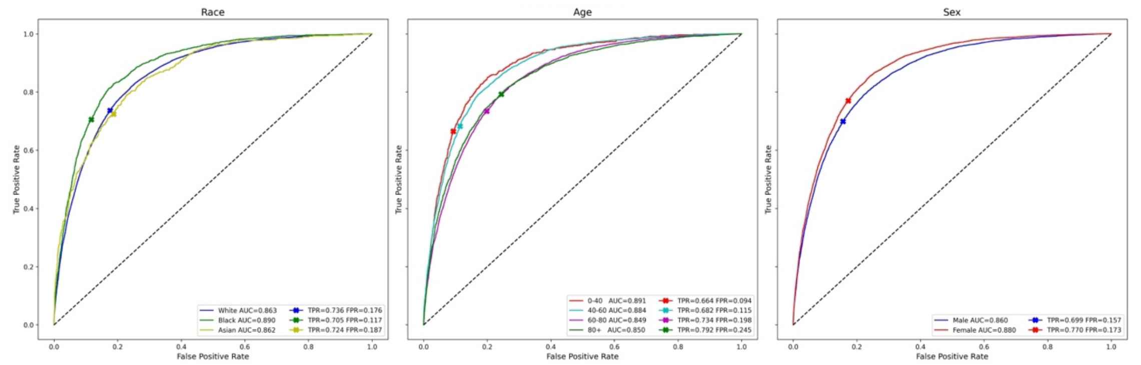 |
| | | Pneumonia |
| w/o | w/o | 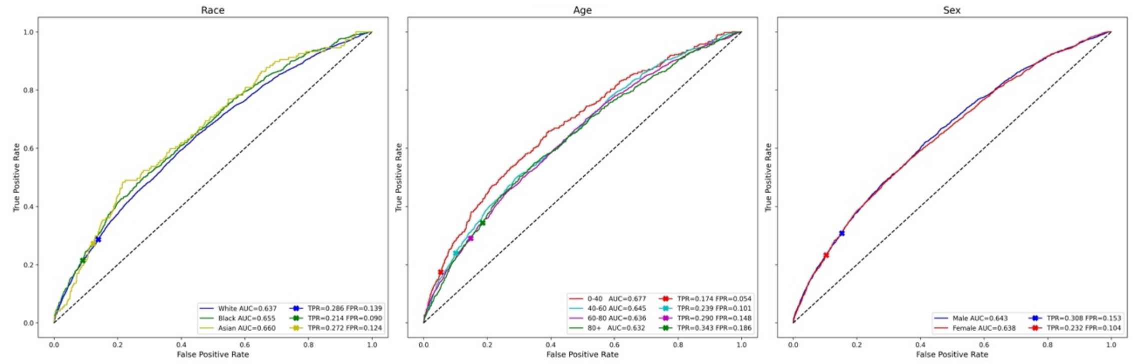 |

| | | |
|---|---|---|
| w/o | w/ | 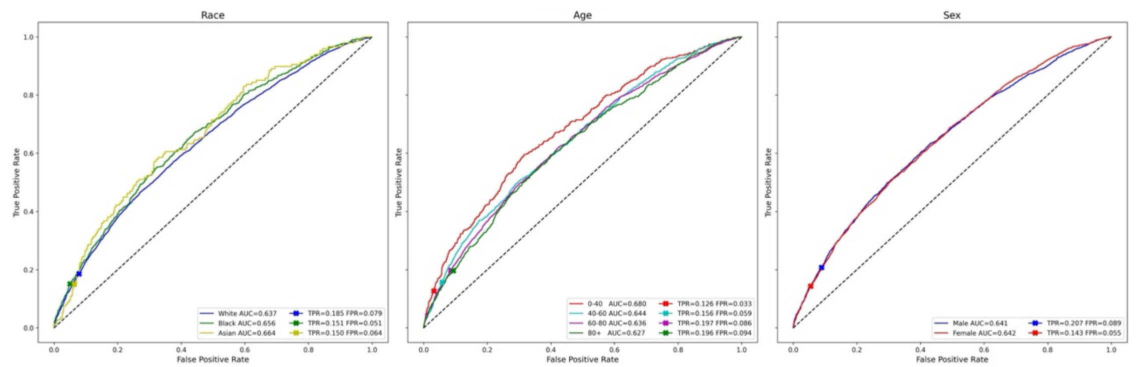 |
| w/ | w/o | 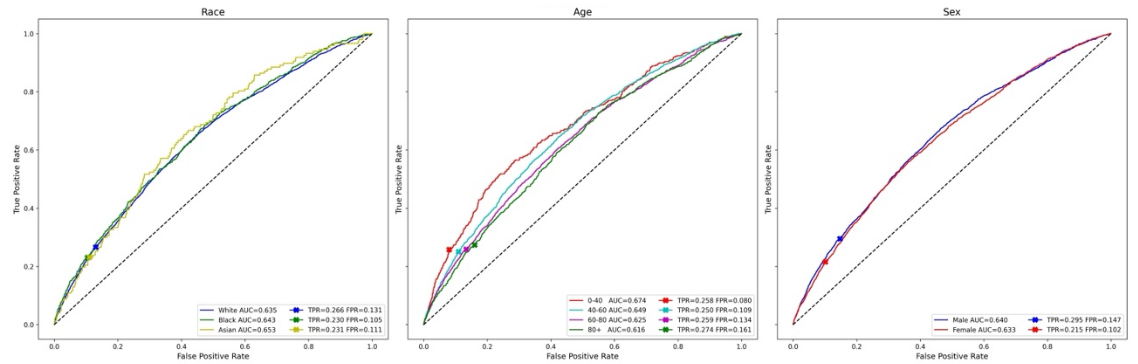 |
| w/ | w/ | 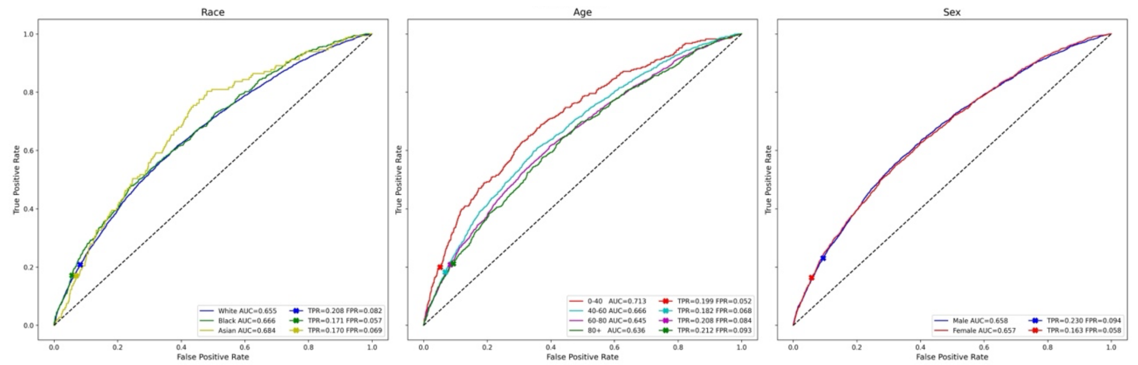 |
| | | Pneumothorax |
| w/o | w/o | 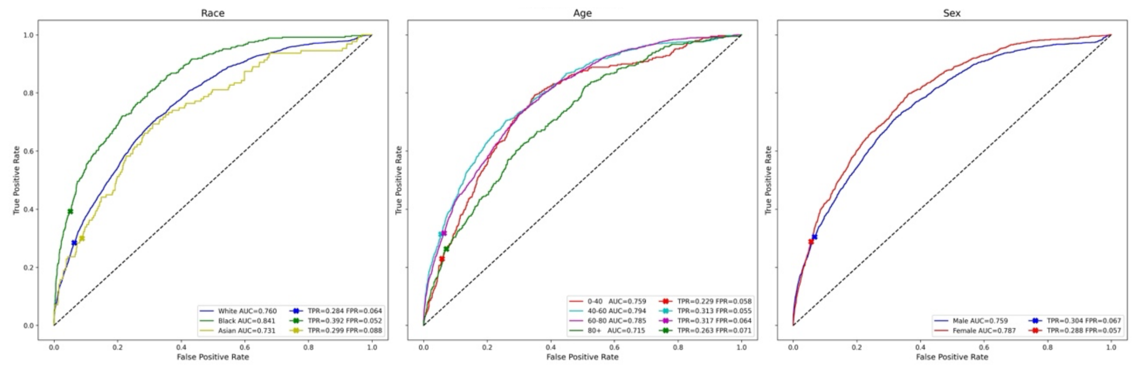 |

<table>
<tr><td>w/o</td><td>w/</td><td>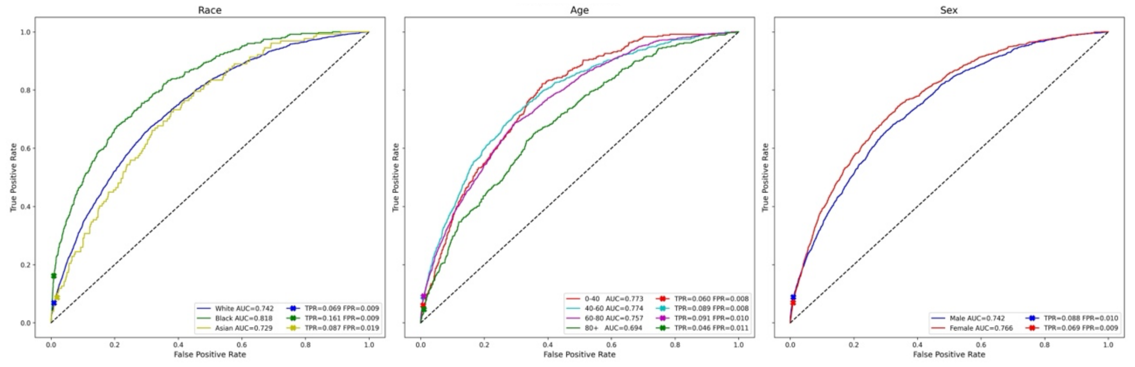
</td></tr>
<tr><td>w/</td><td>w/o</td><td>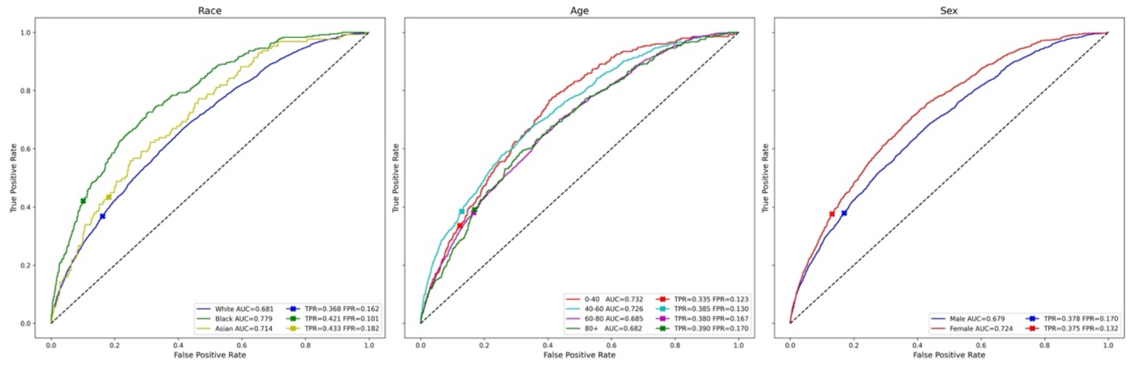
</td></tr>
<tr><td>w/</td><td>w/</td><td>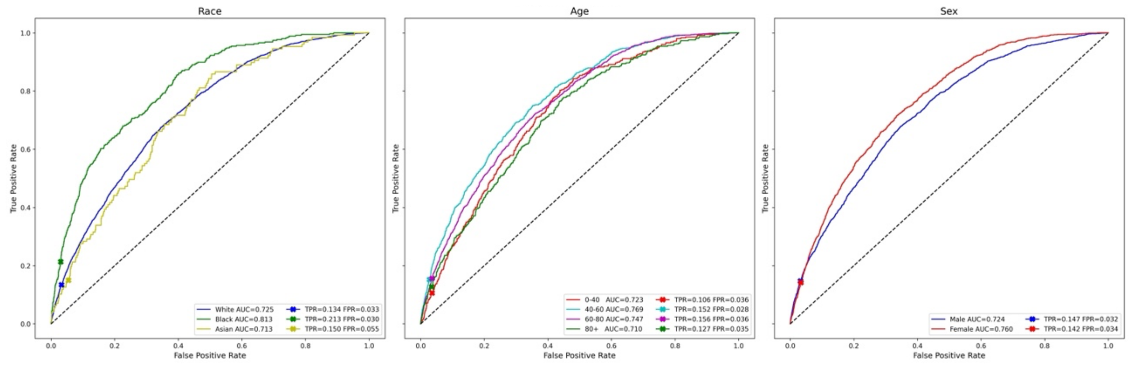
</td></tr>
</table>

Table C2. ROC curve for MRI model

| **Train-time aug.** | **Test-time aug.** | AD |
|---|---|---|
| w/o | w/o | 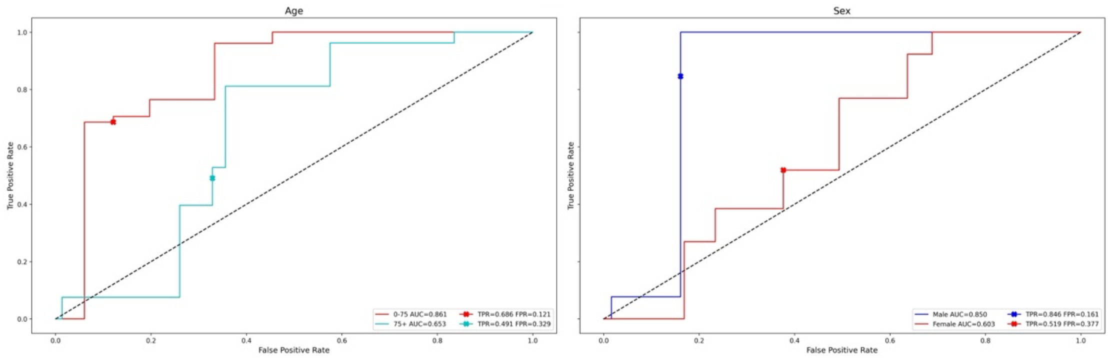 |
| w/o | w/ | 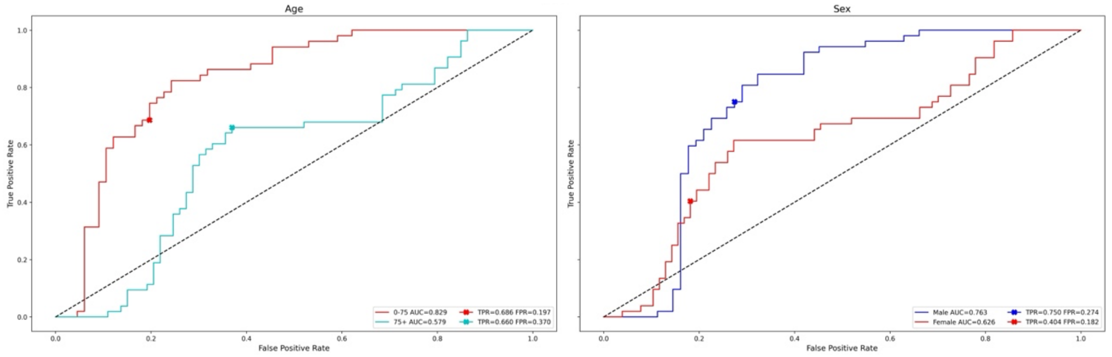 |
| w/ | w/o | 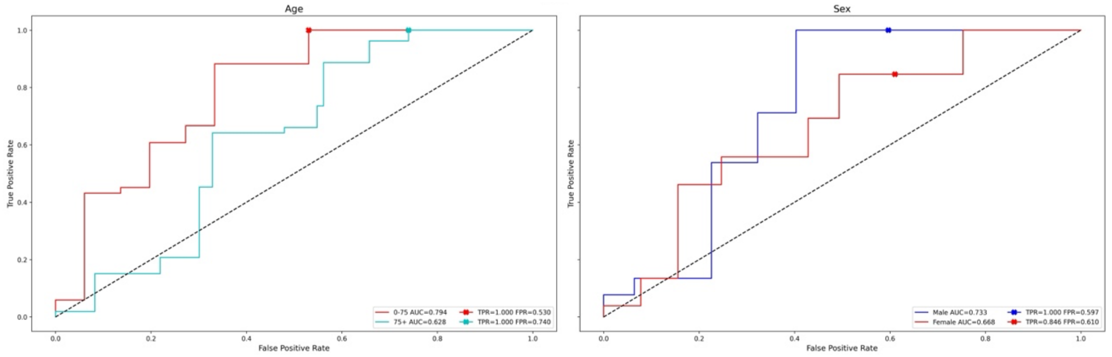 |
| w/ | w/ | 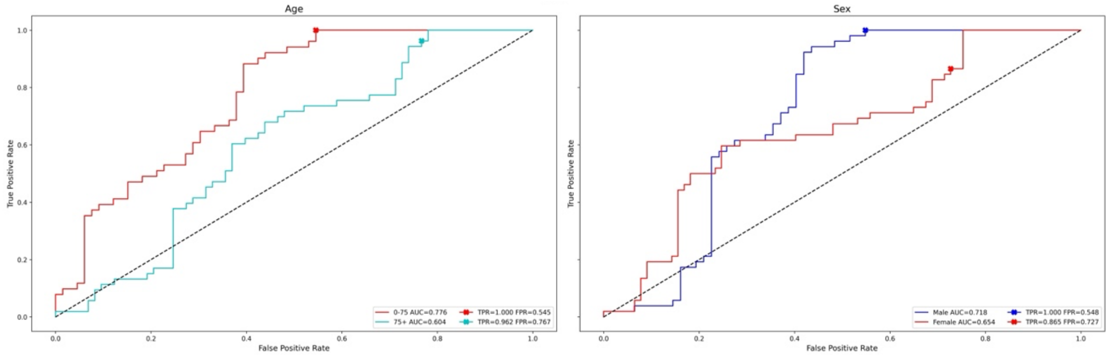 |

# D. Comparison of disparities using different methods and data

We benchmarked several existing debiasing methods using our proposed augmented images to evaluate if our method could further improve the efficacy of the existing methods. We applied our proposed augmentation to training and testing separately and compared them to using only original data. Table D1, D2, and D3 showed the disparities

results in race, age, and sex groups using MIMIC-CXR and DenseNet121 architecture. Table D4 and D5 showed the disparities results in age and sex groups using ADNI brain MRI dataset and ResNet18 architecture.

We quantified the gap of the performance between demographic groups by using the calculation in the prior work.[3] The disparity metrics considers the difference between favour and unfavored groups. For the binary demographic attribute (sex), the disparity for i-th image label was calculated by the difference of performances between male and female:

$disparity_{i,sex} = ABS(performance_{i,female} - performance_{i,male})$. (Equation 1)

For the non-binary demographic attribute (race or age), we calculated the difference between the performance of a certain demographic group and the median of the performance of all demographic groups:

$disparity_{i,race\ or\ age} = \sum_{j\ in\ subgroup} \quad ABS(performance_{i,j} - Median(performance_{i,all}))$. (Equation 2)

We then averaged the disparities across all image labels.

Table D1. Disparities in race of DenseNet121 using MIMIC-CXR.

| Method | Train-time aug. | Test-time aug. | AUC | BCE | ECE | Error rate | Precision |
|---|---|---|---|---|---|---|---|
| Baseline (No previous debias methods applied) | w/o | w/o | 0.040 [-0.020 - 0.099] | 0.063 [-0.018 - 0.144] | 0.015 [-0.012 - 0.042] | 0.055 [0.010 - 0.100] | 0.044 [-0.033 - 0.120] |
| | w/o | w/ | **0.037 [-0.009 - 0.084]** | 0.057 [-0.013 - 0.128] | **0.013 [-0.007 - 0.032]** | **0.047 [-0.002 - 0.096]** | 0.060 [-0.020 - 0.140] |
| | w/ | w/o | 0.035 [-0.016 - 0.086] | 0.058 [-0.019 - 0.134] | 0.018 [-0.003 - 0.039] | 0.052 [0.007 - 0.097] | **0.040 [-0.018 - 0.099]** |
| | w/ | w/ | 0.037 [-0.016 - 0.090] | **0.056 [-0.014 - 0.126]** | 0.014 [-0.001 - 0.028] | 0.052 [0.003 - 0.101] | 0.045 [0.004 - 0.087] |
| Balanced | w/o | w/o | 0.051 [-0.001 - 0.103] | 0.091 [-0.036 - 0.218] | 0.028 [-0.018 - 0.073] | 0.059 [0.002 - 0.117] | 0.051 [-0.031 - 0.133] |
| | w/o | w/ | 0.046 [-0.013 - 0.105] | 0.079 [-0.027 - 0.185] | 0.033 [-0.023 - 0.089] | **0.037 [-0.009 - 0.082]** | 0.081 [-0.127 - 0.289] |
| | w/ | w/o | **0.031 [-0.018 - 0.080]** | 0.086 [-0.112 - 0.285] | **0.025 [-0.036 - 0.085]** | 0.046 [-0.012 - 0.104] | **0.046 [-0.012 - 0.104]** |
| | w/ | w/ | 0.034 [-0.006 - 0.073] | **0.075 [-0.081 - 0.231]** | 0.029 [-0.035 - 0.093] | 0.041 [-0.037 - 0.118] | 0.056 [-0.026 - 0.138] |
| Stratified | w/o | w/ | 0.083 [0.024 - 0.141] | 0.167 [-0.106 - 0.440] | 0.085 [-0.105 - 0.275] | **0.119 [-0.044 - 0.281]** | 0.105 [0.014 - 0.196] |
| | w/o | w/ | 0.112 [0.021 - 0.203] | 0.098 [-0.018 - 0.215] | 0.043 [-0.033 - 0.118] | 0.367 [-0.002 - 0.736] | 0.195 [-0.038 - 0.428] |
| | w/ | w/o | **0.059 [0.006 - 0.113]** | 0.134 [-0.050 - 0.318] | 0.063 [-0.013 - 0.139] | 0.141 [-0.124 - 0.406] | **0.070 [-0.017 - 0.157]** |
| | w/ | w/ | 0.059 [-0.008 | **0.076 [-0.040** | **0.041 [-0.014** | 0.209 [-0.292 | 0.076 [-0.030 |

| | | | | | | | |
|---|---|---|---|---|---|---|---|
| | | | - 0.126] | **- 0.192]** | **- 0.096]** | - 0.710] | - 0.182] |
| Adversarial learning | w/o | w/o | 0.037 [-0.028 - 0.102] | 0.060 [-0.027 - 0.147] | 0.021 [-0.014 - 0.056] | 0.049 [0.006 - 0.091] | 0.047 [-0.005 - 0.098] |
| | w/o | w/ | 0.036 [-0.021 - 0.093] | 0.065 [-0.030 - 0.160] | 0.020 [-0.019 - 0.060] | **0.039 [-0.003 - 0.081]** | 0.059 [-0.001 - 0.119] |
| | w/ | w/o | 0.036 [-0.002 - 0.074] | 0.053 [-0.003 - 0.108] | **0.016 [0.000 - 0.032]** | 0.052 [0.005 - 0.100] | 0.042 [-0.011 - 0.096] |
| | w/ | w/ | **0.035 [0.001 - 0.068]** | **0.050 [-0.003 - 0.102]** | 0.017 [-0.006 - 0.040] | 0.052 [-0.002 - 0.107] | **0.036 [-0.008 - 0.081]** |
| DistMatchMMD | w/o | w/o | 0.042 [-0.050 - 0.134] | 0.093 [-0.068 - 0.255] | 0.038 [-0.042 - 0.118] | 0.042 [-0.027 - 0.110] | 0.045 [-0.047 - 0.136] |
| | w/o | w/ | **0.018 [-0.009 - 0.046]** | 0.091 [-0.055 - 0.236] | 0.039 [-0.044 - 0.122] | **0.026 [-0.019 - 0.072]** | **0.040 [-0.046 - 0.126]** |
| | w/ | w/o | 0.020 [-0.007 - 0.047] | **0.084 [-0.039 - 0.207]** | 0.038 [-0.039 - 0.116] | 0.042 [-0.040 - 0.124] | 0.042 [-0.040 - 0.124] |
| | w/ | w/ | 0.024 [-0.002 - 0.049] | **0.084 [-0.039 - 0.207]** | **0.036 [-0.038 - 0.109]** | 0.042 [-0.039 - 0.123] | 0.042 [-0.040 - 0.124] |
| DistMatchMean | w/o | w/o | 0.034 [-0.007 - 0.075] | **0.060 [-0.040 - 0.160]** | **0.018 [-0.023 - 0.060]** | 0.050 [0.009 - 0.091] | **0.041 [-0.040 - 0.122]** |
| | w/o | w/ | 0.031 [0.006 - 0.056] | 0.065 [-0.060 - 0.190] | 0.023 [-0.025 - 0.072] | 0.036 [-0.002 - 0.073] | 0.046 [-0.026 - 0.118] |
| | w/ | w/o | **0.029 [-0.002 - 0.060]** | 0.064 [-0.059 - 0.188] | 0.026 [-0.017 - 0.069] | 0.039 [-0.001 - 0.078] | 0.046 [-0.039 - 0.131] |
| | w/ | w/ | 0.039 [-0.010 - 0.089] | 0.064 [-0.065 - 0.193] | 0.026 [-0.017 - 0.069] | **0.030 [-0.010 - 0.071]** | 0.053 [-0.028 - 0.134] |
| FairALM | w/o | w/o | **0.027 [-0.006 - 0.060]** | 0.102 [-0.040 - 0.245] | **0.026 [-0.018 - 0.070]** | 0.057 [0.003 - 0.112] | 0.044 [-0.017 - 0.104] |
| | w/o | w/ | 0.033 [-0.017 - 0.082] | 0.080 [-0.037 - 0.197] | 0.028 [-0.024 - 0.079] | **0.044 [-0.004 - 0.092]** | 0.049 [-0.011 - 0.108] |
| | w/ | w/o | 0.037 [-0.022 - 0.097] | 0.083 [-0.035 - 0.202] | 0.032 [-0.029 - 0.093] | 0.052 [0.020 - 0.084] | 0.043 [-0.030 - 0.117] |
| | w/ | w/ | 0.036 [-0.017 - 0.089] | **0.079 [-0.034 - 0.192]** | 0.028 [-0.030 - 0.086] | 0.048 [0.001 - 0.095] | **0.041 [-0.038 - 0.121]** |

Table D2. Disparities in age of DenseNet121 using MIMIC-CXR.

| **Method** | **Train-time aug.** | **Test-time aug.** | **AUC** | **BCE** | **ECE** | **Error rate** | **Precision** |
|---|---|---|---|---|---|---|---|
| Baseline (No previous | w/o | w/o | 0.114 [0.012 - 0.215] | **0.183 [-0.104 - 0.470]** | 0.031 [-0.019 - 0.081] | 0.208 [-0.060 - 0.476] | **0.093 [-0.060 - 0.246]** |

| | | | | | | | |
|---|---|---|---|---|---|---|---|
| debias methods applied) | w/o | w/ | **0.106 [0.013 - 0.200]** | 0.189 [-0.098 - 0.476] | 0.036 [-0.021 - 0.093] | 0.181 [-0.112 - 0.473] | 0.120 [-0.062 - 0.302] |
| | w/ | w/o | 0.125 [0.041 - 0.209] | 0.197 [-0.108 - 0.502] | **0.023 [-0.032 - 0.078]** | 0.179 [-0.040 - 0.397] | 0.099 [-0.091 - 0.289] |
| | w/ | w/ | 0.112 [0.012 - 0.212] | 0.186 [-0.086 - 0.457] | 0.026 [-0.026 - 0.077] | **0.163 [-0.110 - 0.437]** | 0.104 [-0.066 - 0.274] |
| Balanced | w/o | w/o | 0.133 [0.028 - 0.237] | 0.267 [-0.193 - 0.728] | 0.076 [-0.112 - 0.263] | 0.220 [-0.088 - 0.528] | 0.128 [-0.160 - 0.416] |
| | w/o | w/ | **0.112 [0.002 - 0.223]** | 0.242 [-0.142 - 0.626] | 0.078 [-0.132 - 0.288] | 0.172 [-0.065 - 0.409] | 0.172 [-0.131 - 0.475] |
| | w/ | w/o | 0.121 [0.017 - 0.226] | 0.265 [-0.292 - 0.823] | 0.067 [-0.117 - 0.251] | 0.157 [-0.093 - 0.407] | **0.127 [-0.112 - 0.366]** |
| | w/ | w/ | 0.121 [0.033 - 0.210] | **0.233 [-0.210 - 0.675]** | **0.066 [-0.132 - 0.264]** | **0.140 [-0.153 - 0.433]** | 0.134 [-0.114 - 0.382] |
| Stratified | w/o | w/o | 0.137 [0.021 - 0.252] | 0.191 [-0.064 - 0.446] | **0.073 [-0.103 - 0.249]** | **0.277 [-0.001 - 0.555]** | 0.143 [-0.100 - 0.386] |
| | w/o | w/ | **0.129 [0.007 - 0.251]** | **0.178 [-0.049 - 0.404]** | 0.084 [-0.049 - 0.218] | 0.376 [-0.162 - 0.914] | 0.186 [-0.075 - 0.446] |
| | w/ | w/o | 0.137 [0.037 - 0.238] | 0.187 [-0.116 - 0.490] | 0.075 [-0.091 - 0.240] | 0.342 [-0.084 - 0.769] | 0.135 [-0.169 - 0.438] |
| | w/ | w/ | 0.142 [0.044 - 0.241] | 0.230 [-0.160 - 0.619] | 0.126 [-0.155 - 0.407] | 0.550 [-0.143 - 1.243] | **0.134 [-0.093 - 0.361]** |
| Adversarial learning | w/o | w/o | 0.113 [0.026 - 0.200] | 0.197 [-0.087 - 0.481] | 0.050 [-0.043 - 0.143] | 0.143 [-0.052 - 0.337] | 0.114 [-0.071 - 0.300] |
| | w/o | w/ | **0.107 [0.011 - 0.202]** | 0.214 [-0.114 - 0.542] | 0.062 [-0.087 - 0.211] | **0.131 [-0.082 - 0.345]** | 0.127 [-0.098 - 0.351] |
| | w/ | w/o | 0.125 [0.052 - 0.199] | 0.186 [-0.085 - 0.457] | **0.030 [-0.021 - 0.081]** | 0.174 [-0.057 - 0.405] | 0.093 [-0.096 - 0.282] |
| | w/ | w/ | 0.116 [0.026 - 0.206] | **0.180 [-0.073 - 0.433]** | 0.032 [-0.026 - 0.090] | 0.181 [-0.079 - 0.441] | **0.089 [-0.072 - 0.250]** |
| DistMatchMMD | w/o | w/o | 0.089 [-0.016 - 0.194] | 0.300 [-0.259 - 0.858] | **0.130 [-0.156 - 0.415]** | 0.098 [-0.020 - 0.217] | **0.142 [-0.157 - 0.441]** |
| | w/o | w/ | 0.023 [-0.007 - 0.052] | 0.301 [-0.216 - 0.818] | 0.135 [-0.160 - 0.431] | **0.065 [-0.071 - 0.202]** | 0.151 [-0.144 - 0.446] |
| | w/ | w/o | **0.023 [-0.003 - 0.049]** | **0.285 [-0.194 - 0.764]** | 0.136 [-0.153 - 0.426] | 0.150 [-0.149 - 0.448] | 0.149 [-0.151 - 0.450] |
| | w/ | w/ | 0.054 [0.010 - 0.098] | **0.285 [-0.194 - 0.764]** | 0.133 [-0.152 - 0.418] | 0.150 [-0.150 - 0.450] | 0.149 [-0.152 - 0.451] |
| DistMatchMean | w/o | w/o | 0.109 [-0.011 - 0.230] | **0.199 [-0.141 - 0.538]** | **0.050 [-0.070 - 0.171]** | 0.146 [-0.057 - 0.349] | **0.113 [-0.101 - 0.327]** |

| | | | | | | | |
|---|---|---|---|---|---|---|---|
| | w/o | w/ | **0.098 [-0.012 - 0.209]** | 0.208 [-0.181 - 0.597] | 0.070 [-0.081 - 0.220] | **0.123 [-0.068 - 0.315]** | 0.132 [-0.102 - 0.367] |
| | w/ | w/o | 0.122 [0.028 - 0.215] | 0.210 [-0.186 - 0.605] | 0.072 [-0.068 - 0.212] | 0.168 [-0.009 - 0.345] | 0.122 [-0.148 - 0.393] |
| | w/ | w/ | 0.110 [0.000 - 0.220] | 0.203 [-0.195 - 0.601] | 0.072 [-0.086 - 0.230] | 0.141 [-0.083 - 0.366] | 0.127 [-0.154 - 0.408] |
| FairALM | w/o | w/o | **0.098 [0.016 - 0.179]** | **0.240 [-0.213 - 0.693]** | **0.061 [-0.063 - 0.186]** | 0.180 [-0.088 - 0.449] | **0.097 [-0.109 - 0.304]** |
| | w/o | w/ | 0.108 [0.037 - 0.179] | 0.254 [-0.134 - 0.642] | 0.084 [-0.101 - 0.269] | **0.170 [-0.139 - 0.480]** | 0.120 [-0.154 - 0.395] |
| | w/ | w/o | 0.124 [0.036 - 0.211] | 0.269 [-0.160 - 0.699] | 0.097 [-0.126 - 0.320] | 0.197 [-0.029 - 0.423] | 0.110 [-0.118 - 0.337] |
| | w/ | w/ | 0.124 [0.031 - 0.217] | 0.267 [-0.168 - 0.703] | 0.089 [-0.130 - 0.308] | 0.230 [-0.022 - 0.482] | 0.101 [-0.084 - 0.286] |

Table D3. Disparities in sex of DenseNet121 using MIMIC-CXR.

| Method | **Train-time aug.** | **Test-time aug.** | **AUC** | **BCE** | **ECE** | **Error rate** | **Precision** |
|---|---|---|---|---|---|---|---|
| Baseline (No previous debias methods applied) | w/o | w/o | 0.010 [-0.009 - 0.030] | 0.025 [-0.019 - 0.069] | 0.013 [-0.014 - 0.039] | 0.027 [-0.009 - 0.063] | 0.020 [-0.017 - 0.057] |
| | w/o | w/ | **0.010 [-0.008 - 0.029]** | **0.020 [-0.014 - 0.054]** | 0.009 [-0.006 - 0.023] | 0.023 [-0.015 - 0.061] | 0.035 [-0.041 - 0.110] |
| | w/ | w/o | 0.014 [-0.014 - 0.042] | 0.021 [-0.009 - 0.051] | **0.007 [-0.005 - 0.019]** | 0.021 [-0.014 - 0.055] | **0.016 [-0.012 - 0.045]** |
| | w/ | w/ | 0.013 [-0.011 - 0.037] | 0.021 [-0.012 - 0.054] | 0.007 [-0.007 - 0.021] | **0.015 [-0.014 - 0.044]** | 0.026 [-0.020 - 0.072] |
| Balanced | w/o | w/o | 0.015 [-0.009 - 0.039] | 0.038 [-0.024 - 0.100] | 0.013 [-0.015 - 0.040] | 0.031 [-0.032 - 0.094] | **0.015 [-0.011 - 0.040]** |
| | w/o | w/ | **0.015 [-0.005 - 0.035]** | 0.033 [-0.019 - 0.084] | 0.013 [-0.013 - 0.040] | 0.024 [-0.017 - 0.064] | 0.017 [-0.019 - 0.054] |
| | w/ | w/o | 0.018 [-0.019 - 0.055] | 0.032 [-0.006 - 0.071] | 0.011 [-0.011 - 0.033] | 0.021 [-0.014 - 0.055] | 0.020 [-0.009 - 0.049] |
| | w/ | w/ | 0.015 [-0.016 - 0.046] | **0.025 [-0.008 - 0.057]** | **0.007 [-0.004 - 0.017]** | **0.010 [-0.010 - 0.030]** | 0.018 [-0.008 - 0.043] |
| Stratified | w/o | w/o | **0.011 [-0.005 - 0.027]** | **0.022 [-0.037 - 0.081]** | 0.020 [-0.001 - 0.040] | **0.028 [-0.018 - 0.074]** | 0.033 [-0.009 - 0.076] |
| | w/o | w/ | 0.014 [-0.005 - 0.032] | 0.024 [-0.039 - 0.087] | 0.018 [-0.014 - 0.050] | 0.064 [-0.035 - 0.164] | 0.052 [-0.027 - 0.132] |
| | w/ | w/o | 0.029 [-0.015 | 0.032 [-0.021 | **0.013 [-0.016** | 0.033 [-0.029 | **0.026 [-0.019** |

| | | | | | | | |
|---|---|---|---|---|---|---|---|
| | | | - 0.072] | - 0.084] | **- 0.043]** | - 0.095] | **- 0.071]** |
| | w/ | w/ | 0.026 [0.002 - 0.049] | 0.029 [-0.011 - 0.070] | 0.016 [-0.022 - 0.054] | 0.057 [-0.001 - 0.115] | 0.035 [-0.059 - 0.129] |
| Adversarial learning | w/o | w/o | **0.011 [-0.004 - 0.026]** | 0.020 [-0.014 - 0.054] | 0.008 [-0.014 - 0.030] | **0.015 [-0.006 - 0.036]** | **0.020 [-0.026 - 0.066]** |
| | w/o | w/ | 0.014 [-0.004 - 0.033] | 0.023 [-0.011 - 0.058] | **0.008 [-0.010 - 0.026]** | **0.015 [-0.006 - 0.036]** | 0.021 [-0.031 - 0.073] |
| | w/ | w/o | 0.013 [-0.009 - 0.034] | 0.020 [-0.010 - 0.049] | 0.012 [-0.015 - 0.039] | 0.031 [-0.028 - 0.089] | 0.022 [-0.007 - 0.051] |
| | w/ | w/ | 0.011 [-0.004 - 0.027] | **0.018 [-0.006 - 0.042]** | 0.014 [-0.019 - 0.048] | 0.037 [-0.018 - 0.091] | 0.024 [-0.020 - 0.068] |
| DistMatchMMD | w/o | w/o | 0.020 [-0.004 - 0.043] | 0.030 [-0.012 - 0.072] | **0.011 [-0.008 - 0.030]** | 0.033 [-0.037 - 0.103] | 0.015 [-0.006 - 0.035] |
| | w/o | w/ | **0.004 [-0.004 - 0.012]** | 0.029 [-0.008 - 0.067] | 0.012 [-0.006 - 0.030] | 0.015 [-0.011 - 0.041] | 0.014 [-0.003 - 0.031] |
| | w/ | w/o | 0.008 [-0.001 - 0.017] | **0.028 [-0.006 - 0.063]** | 0.012 [-0.005 - 0.029] | 0.013 [-0.006 - 0.032] | **0.012 [-0.005 - 0.030]** |
| | w/ | w/ | 0.016 [-0.013 - 0.045] | **0.028 [-0.006 - 0.063]** | 0.012 [-0.005 - 0.029] | **0.012 [-0.006 - 0.030]** | 0.013 [-0.005 - 0.030] |
| DistMatchMean | w/o | w/o | **0.011 [-0.004 - 0.026]** | **0.022 [-0.005 - 0.049]** | **0.008 [-0.004 - 0.020]** | **0.018 [-0.016 - 0.052]** | 0.020 [-0.007 - 0.047] |
| | w/o | w/ | 0.012 [-0.002 - 0.026] | 0.022 [-0.009 - 0.053] | 0.008 [-0.005 - 0.021] | 0.020 [-0.004 - 0.044] | 0.023 [-0.015 - 0.061] |
| | w/ | w/o | 0.014 [-0.012 - 0.039] | 0.026 [-0.013 - 0.066] | 0.010 [-0.009 - 0.029] | 0.032 [-0.012 - 0.076] | **0.019 [-0.027 - 0.065]** |
| | w/ | w/ | 0.015 [-0.006 - 0.035] | 0.026 [-0.014 - 0.066] | 0.010 [-0.013 - 0.032] | 0.043 [-0.027 - 0.113] | 0.022 [-0.030 - 0.074] |
| FairALM | w/o | w/o | 0.014 [-0.021 - 0.049] | 0.035 [-0.069 - 0.139] | **0.008 [-0.005 - 0.021]** | 0.016 [-0.016 - 0.047] | 0.020 [-0.013 - 0.054] |
| | w/o | w/ | 0.011 [-0.010 - 0.032] | 0.028 [-0.011 - 0.067] | 0.010 [-0.006 - 0.027] | **0.014 [0.001 - 0.027]** | 0.026 [-0.035 - 0.087] |
| | w/ | w/o | **0.009 [-0.009 - 0.028]** | **0.028 [-0.005 - 0.061]** | 0.010 [-0.009 - 0.029] | 0.022 [-0.019 - 0.063] | **0.016 [-0.021 - 0.052]** |
| | w/ | w/ | 0.010 [-0.011 - 0.032] | 0.028 [-0.008 - 0.063] | 0.011 [-0.013 - 0.034] | 0.033 [-0.008 - 0.074] | 0.022 [-0.036 - 0.080] |

Table D4. Disparities in age of ResNet18 using ADNI brain MRI.

| Method | **Train-time aug.** | **Test-time aug.** | **AUC** | **BCE** | **ECE** | **Error rate** | **Precision** |
|---|---|---|---|---|---|---|---|

| | | | | | | | |
|---|---|---|---|---|---|---|---|
| Baseline (No previous debias methods applied) | w/o | w/o | 0·209 | 0·322 | 0·109 | 0·273 | 0·428 |
| | w/o | w/ | 0·251 | **0·109** | **0·024** | 0·173 | 0·226 |
| | w/ | w/o | **0·163** | 0·253 | 0·070 | **0·128** | **0·095** |
| | w/ | w/ | **0.170** | 0.038 | 0.012 | **0.151** | **0.107** |
| Balanced | w/o | w/o | 0·182 | 0·110 | **0·013** | 0·021 | 0·018 |
| | w/o | w/ | **0·127** | **0·101** | **0·013** | 0·144 | 0·124 |
| | w/ | w/o | 0·297 | 0·124 | **0·013** | **0·013** | **0·013** |
| | w/ | w/ | 0.215 | 0.103 | **0.013** | **0.013** | **0.013** |
| Stratified | w/o | w/o | 0·280 | 0·143 | **0·017** | 0·148 | 0·296 |
| | w/o | w/ | 0·209 | **0·073** | 0·025 | **0·002** | 0·337 |
| | w/ | w/o | 0·170 | 1·048 | 0·018 | 0·013 | **0·013** |
| | w/ | w/ | **0.136** | 0.974 | 0.019 | 0.013 | **0.013** |
| Adversarial learning | w/o | w/o | 0·120 | 0·084 | 0·016 | 0·031 | 0·022 |
| | w/o | w/ | **0·110** | **0·072** | 0·016 | **0·013** | **0·013** |
| | w/ | w/o | 0·175 | 0·087 | 0·015 | **0·013** | **0·013** |
| | w/ | w/ | 0.149 | 0.085 | **0.014** | **0.013** | **0.013** |
| DistMatchMMD | w/o | w/o | 0·159 | **0·042** | 0·049 | **0·032** | **0·039** |
| | w/o | w/ | **0·055** | 0·056 | 0·018 | 0·097 | 0·092 |
| | w/ | w/o | 0·090 | 0·200 | **0·002** | 0·122 | 0·117 |
| | w/ | w/ | 0.132 | 0.263 | 0.004 | 0.072 | 0.099 |
| DistMatchMean | w/o | w/o | 0·074 | 0·090 | **0·013** | **0·021** | **0·018** |
| | w/o | w/ | **0·054** | **0·080** | **0·013** | 0·045 | 0·047 |
| | w/ | w/o | 0·221 | 0·095 | **0·013** | 0·078 | 0·050 |
| | w/ | w/ | 0.073 | 0.088 | **0.013** | 0.039 | 0.025 |
| FairALM | w/o | w/o | 0·216 | 0·316 | 0·114 | 0·162 | 0·241 |
| | w/o | w/ | 0·190 | 0·160 | **0·001** | **0·030** | 0·139 |
| | w/ | w/o | **0·086** | **0·004** | 0·004 | 0·102 | 0·067 |
| | w/ | w/ | 0.191 | 0.048 | 0.007 | 0.056 | **0.045** |

Table D5. Disparities in sex of ResNet18 using ADNI brain MRI.

| Method | **Train-time** | **Test-time** | **AUC** | **BCE** | **ECE** | **Error rate** | **Precision** |
|---|---|---|---|---|---|---|---|

| | aug. | aug. | | | | | |
|---|---|---|---|---|---|---|---|
| Baseline (No previous debias methods applied) | w/o | w/o | 0.247 | 0.369 | **0.033** | 0.258 | 0.373 |
| | w/o | w/ | 0.138 | **0.038** | 0.062 | **0.124** | 0.182 |
| | w/ | w/o | 0.065 | 0.076 | 0.042 | 0.178 | 0.156 |
| | w/ | w/ | **0.064** | 0.055 | 0.1 | 0.168 | **0.144** |
| Balanced | w/o | w/o | 0.007 | 0.404 | **0.056** | 0.064 | 0.061 |
| | w/o | w/ | 0.149 | 0.378 | **0.056** | 0.135 | 0.057 |
| | w/ | w/o | **0** | 0.361 | **0.056** | **0.056** | **0.056** |
| | w/ | w/ | 0.069 | **0.353** | **0.056** | **0.056** | **0.056** |
| Stratified | w/o | w/o | 0.202 | 0.376 | 0.057 | 0.117 | 0.154 |
| | w/o | w/ | 0.172 | **0.166** | **0.022** | **0.051** | 0.154 |
| | w/ | w/o | 0.026 | 0.395 | 0.055 | 0.056 | **0.056** |
| | w/ | w/ | **0.017** | 0.495 | 0.058 | 0.056 | **0.056** |
| Adversarial learning | w/o | w/o | 0.074 | 0.281 | 0.057 | **0.025** | **0.043** |
| | w/o | w/ | **0.04** | **0.279** | **0.056** | 0.056 | 0.056 |
| | w/ | w/o | 0.057 | 0.296 | 0.057 | 0.056 | 0.056 |
| | w/ | w/ | 0.059 | 0.301 | **0.056** | 0.056 | 0.056 |
| DistMatchMMD | w/o | w/o | **0.059** | **0.034** | 0.029 | 0.209 | 0.153 |
| | w/o | w/ | 0.067 | 0.165 | **0.024** | **0.059** | **0.1** |
| | w/ | w/o | 0.099 | 0.313 | 0.044 | 0.138 | 0.152 |
| | w/ | w/ | 0.076 | 0.365 | 0.047 | 0.086 | 0.138 |
| DistMatchMean | w/o | w/o | 0.072 | 0.358 | **0.056** | 0.064 | 0.061 |
| | w/o | w/ | 0.07 | **0.351** | **0.056** | **0.001** | **0.038** |
| | w/ | w/o | 0.216 | 0.376 | **0.056** | 0.104 | 0.085 |
| | w/ | w/ | **0.027** | 0.366 | **0.056** | 0.066 | 0.061 |
| FairALM | w/o | w/o | 0.254 | 0.269 | 0.093 | 0.211 | 0.283 |
| | w/o | w/ | 0.13 | 0.098 | **0.007** | **0.005** | 0.114 |
| | w/ | w/o | **0.002** | **0.087** | 0.041 | 0.011 | **0.04** |
| | w/ | w/ | 0.035 | 0.112 | 0.049 | 0.051 | 0.069 |

Table D6 showed the disparities results of using ResNet50 architecture on MIMIC-CXR, and Table D7 showed the disparities results of using DenseNet121 architecture on CheXpert dataset. Table D8 showed the

disparities results of the model without ImageNet pretrained weight. The disparities were similar between the model with pre-trained weights and the model without pre-trained weights. The comparison between the original models with and without pre-trained weights suggest that the pre-trained weights did not introduce bias.

Table D6. Disparities of ResNet50 using MIMIC-CXR.

| Group | **Train-time aug.** | **Test-time aug.** | **AUC** | **BCE** | **ECE** | **Error rate** | **Precision** |
|---|---|---|---|---|---|---|---|
| Race | w/o | w/o | 0.037 [-0.011 - 0.085] | 0.071 [-0.049 - 0.191] | 0.024 [-0.024 - 0.073] | 0.060 [0.017 - 0.103] | 0.042 [-0.019 - 0.104] |
| | w/o | w/ | 0.035 [-0.023 - 0.092] | 0.064 [-0.048 - 0.175] | **0.018 [-0.018 - 0.053]** | **0.040 [-0.004 - 0.085]** | **0.040 [-0.039 - 0.119]** |
| | w/ | w/o | **0.030 [0.007 - 0.053]** | 0.075 [-0.048 - 0.198] | 0.026 [-0.020 - 0.073] | 0.049 [0.008 - 0.090] | 0.044 [-0.039 - 0.126] |
| | w/ | w/ | 0.037 [-0.006 - 0.079] | **0.061 [-0.026 - 0.148]** | 0.019 [-0.009 - 0.048] | 0.048 [0.002 - 0.093] | 0.041 [-0.046 - 0.127] |
| Age | w/o | w/o | 0.116 [0.026 - 0.206] | 0.220 [-0.167 - 0.608] | 0.059 [-0.079 - 0.198] | 0.224 [0.017 - 0.431] | **0.095 [-0.087 - 0.278]** |
| | w/o | w/ | **0.093 [-0.009 - 0.195]** | 0.201 [-0.152 - 0.554] | **0.052 [-0.072 - 0.176]** | **0.199 [-0.024 - 0.422]** | 0.103 [-0.069 - 0.274] |
| | w/ | w/o | 0.117 [0.015 - 0.220] | 0.217 [-0.115 - 0.550] | 0.063 [-0.072 - 0.199] | 0.226 [0.015 - 0.437] | 0.115 [-0.152 - 0.383] |
| | w/ | w/ | 0.106 [0.000 - 0.211] | **0.197 [-0.107 - 0.501]** | 0.053 [-0.050 - 0.156] | 0.260 [-0.017 - 0.537] | 0.110 [-0.130 - 0.350] |
| sex | w/o | w/o | **0.011 [-0.016 - 0.037]** | 0.026 [-0.003 - 0.056] | 0.011 [-0.003 - 0.024] | **0.016 [-0.012 - 0.044]** | 0.019 [-0.013 - 0.051] |
| | w/o | w/ | 0.014 [-0.020 - 0.048] | **0.023 [-0.011 - 0.056]** | **0.006 [-0.002 - 0.015]** | 0.018 [-0.006 - 0.042] | 0.022 [-0.013 - 0.056] |
| | w/ | w/o | 0.016 [-0.033 - 0.064] | 0.029 [-0.043 - 0.101] | 0.008 [-0.006 - 0.021] | 0.024 [-0.019 - 0.068] | **0.011 [-0.023 - 0.045]** |
| | w/ | w/ | 0.016 [-0.029 - 0.061] | 0.023 [-0.015 - 0.062] | 0.008 [-0.009 - 0.025] | 0.025 [-0.010 - 0.059] | 0.013 [-0.022 - 0.048] |

Table D7. Disparities of DenseNet121 using CheXpert CXR.

| Group | **Train-time aug.** | **Test-time aug.** | **AUC** | **BCE** | **ECE** | **Error rate** | **Precision** |
|---|---|---|---|---|---|---|---|

| | | | | | | | |
|---|---|---|---|---|---|---|---|
| Race | w/o | w/o | 0.037 [-0.033 - 0.107] | 0.050 [-0.037 - 0.136] | 0.016 [-0.015 - 0.048] | **0.025 [-0.010 - 0.060]** | 0.055 [-0.005 - 0.115] |
| | w/o | w/ | **0.027 [-0.006 - 0.061]** | 0.062 [-0.083 - 0.208] | 0.021 [-0.027 - 0.070] | 0.030 [-0.022 - 0.082] | 0.086 [-0.094 - 0.266] |
| | w/ | w/o | 0.031 [-0.013 - 0.074] | **0.049 [-0.031 - 0.128]** | **0.015 [-0.007 - 0.038]** | 0.032 [-0.012 - 0.076] | **0.045 [-0.054 - 0.143]** |
| | w/ | w/ | 0.033 [-0.037 - 0.103] | 0.052 [-0.033 - 0.136] | 0.017 [-0.014 - 0.048] | 0.032 [-0.012 - 0.076] | 0.049 [-0.058 - 0.156] |
| Age | w/o | w/o | 0.080 [0.013 - 0.146] | 0.142 [-0.067 - 0.350] | **0.029 [-0.027 - 0.085]** | **0.094 [-0.015 - 0.203]** | **0.078 [-0.063 - 0.219]** |
| | w/o | w/ | 0.082 [0.013 - 0.151] | 0.160 [-0.126 - 0.446] | 0.044 [-0.060 - 0.147] | 0.094 [-0.024 - 0.212] | 0.112 [-0.152 - 0.377] |
| | w/ | w/o | 0.077 [-0.023 - 0.176] | 0.142 [-0.073 - 0.357] | 0.036 [-0.035 - 0.107] | 0.117 [-0.043 - 0.276] | 0.098 [-0.105 - 0.301] |
| | w/ | w/ | **0.076 [-0.017 - 0.170]** | **0.141 [-0.078 - 0.359]** | 0.045 [-0.034 - 0.124] | 0.099 [-0.052 - 0.250] | 0.101 [-0.146 - 0.348] |
| sex | w/o | w/o | **0.011 [-0.016 - 0.037]** | 0.026 [-0.003 - 0.056] | 0.011 [-0.003 - 0.024] | **0.016 [-0.012 - 0.044]** | 0.019 [-0.013 - 0.051] |
| | w/o | w/ | 0.014 [-0.020 - 0.048] | **0.023 [-0.011 - 0.056]** | **0.006 [-0.002 - 0.015]** | 0.018 [-0.006 - 0.042] | 0.022 [-0.013 - 0.056] |
| | w/ | w/o | 0.016 [-0.033 - 0.064] | 0.029 [-0.043 - 0.101] | 0.008 [-0.006 - 0.021] | 0.024 [-0.019 - 0.068] | **0.011 [-0.023 - 0.045]** |
| | w/ | w/ | 0.016 [-0.029 - 0.061] | 0.023 [-0.015 - 0.062] | 0.008 [-0.009 - 0.025] | 0.025 [-0.010 - 0.059] | 0.013 [-0.022 - 0.048] |

Table D8. Disparities of DenseNet121 using MIMIC CXR (without ImageNet pretrain weight)

| Method | **Train-time aug.** | **Test-time aug.** | **AUC** | **BCE** | **ECE** | **Error rate** | **Precision** |
|---|---|---|---|---|---|---|---|
| No pretrained weight (Race) | w/o | w/o | 0.036 [-0.003 - 0.074] | 0.052 [-0.004 - 0.107] | 0.018 [-0.010 - 0.046] | 0.047 [0.003 - 0.091] | 0.045 [-0.022 - 0.113] |
| | w/o | w/ | 0.034 [-0.006 - 0.074] | 0.050 [-0.002 - 0.103] | 0.018 [-0.007 - 0.043] | 0.037 [0.002 - 0.072] | 0.062 [-0.022 - 0.146] |
| | w/ | w/o | 0.031 [-0.021 - 0.083] | 0.071 [-0.037 - 0.179] | 0.025 [-0.012 - 0.062] | 0.053 [0.017 - 0.088] | 0.045 [-0.033 - 0.123] |

| | | | | | | | |
|---|---|---|---|---|---|---|---|
| | w/ | w/ | 0.038 [-0.018 - 0.095] | 0.076 [-0.064 - 0.217] | 0.031 [-0.027 - 0.088] | 0.048 [-0.009 - 0.106] | 0.050 [-0.020 - 0.120] |
| No pretrained weight (Age) | w/o | w/o | 0.127 [0.039 - 0.215] | 0.188 [- 0.080 - 0.456] | 0.039 [- 0.014 - 0.091] | 0.173 [- 0.039 - 0.384] | 0.092 [- 0.099 - 0.283] |
| | w/o | w/ | 0.120 [0.039 - 0.202] | 0.182 [- 0.080 - 0.443] | 0.046 [- 0.061 - 0.153] | 0.144 [- 0.077 - 0.364] | 0.131 [- 0.127 - 0.389] |
| | w/ | w/o | 0.134 [0.048 - 0.220] | 0.229 [-0.164 - 0.622] | 0.063 [-0.046 - 0.171] | 0.218 [0.003 - 0.432] | 0.104 [-0.149 - 0.356] |
| | w/ | w/ | 0.106 [-0.001 - 0.213] | 0.230 [-0.182 - 0.641] | 0.080 [-0.075 - 0.236] | 0.208 [-0.076 - 0.491] | 0.113 [-0.115 - 0.341] |
| No pretrained weight (sex) | w/o | w/o | 0.011 [- 0.008 - 0.029] | 0.017 [- 0.009 - 0.044] | 0.006 [- 0.005 - 0.016] | 0.030 [- 0.010 - 0.070] | 0.019 [- 0.010 - 0.048] |
| | w/o | w/ | 0.011 [- 0.005 - 0.026] | 0.016 [- 0.010 - 0.043] | 0.007 [- 0.006 - 0.020] | 0.024 [- 0.016 - 0.063] | 0.029 [- 0.023 - 0.081] |
| | w/ | w/o | 0.011 [-0.019 - 0.040] | 0.024 [-0.012 - 0.060] | 0.009 [-0.005 - 0.022] | 0.025 [-0.004 - 0.053] | 0.013 [-0.020 - 0.047] |
| | w/ | w/ | 0.016 [-0.018 - 0.050] | 0.028 [-0.010 - 0.065] | 0.010 [-0.007 - 0.026] | 0.024 [-0.001 - 0.049] | 0.019 [-0.019 - 0.057] |

## E. Performance and disparities in the detection of CXR labels

The subsequent figures depict the performance and fairness gap of the 2D Chest X-Ray (CXR) model proposed in this study, juxtaposed with other debiasing methods. This section presents the outcomes for nine other pulmonary conditions, with the figure for Edema detailed in the main body of the text. The configuration of these figures is consistent with Figure 2 as described in the main document.

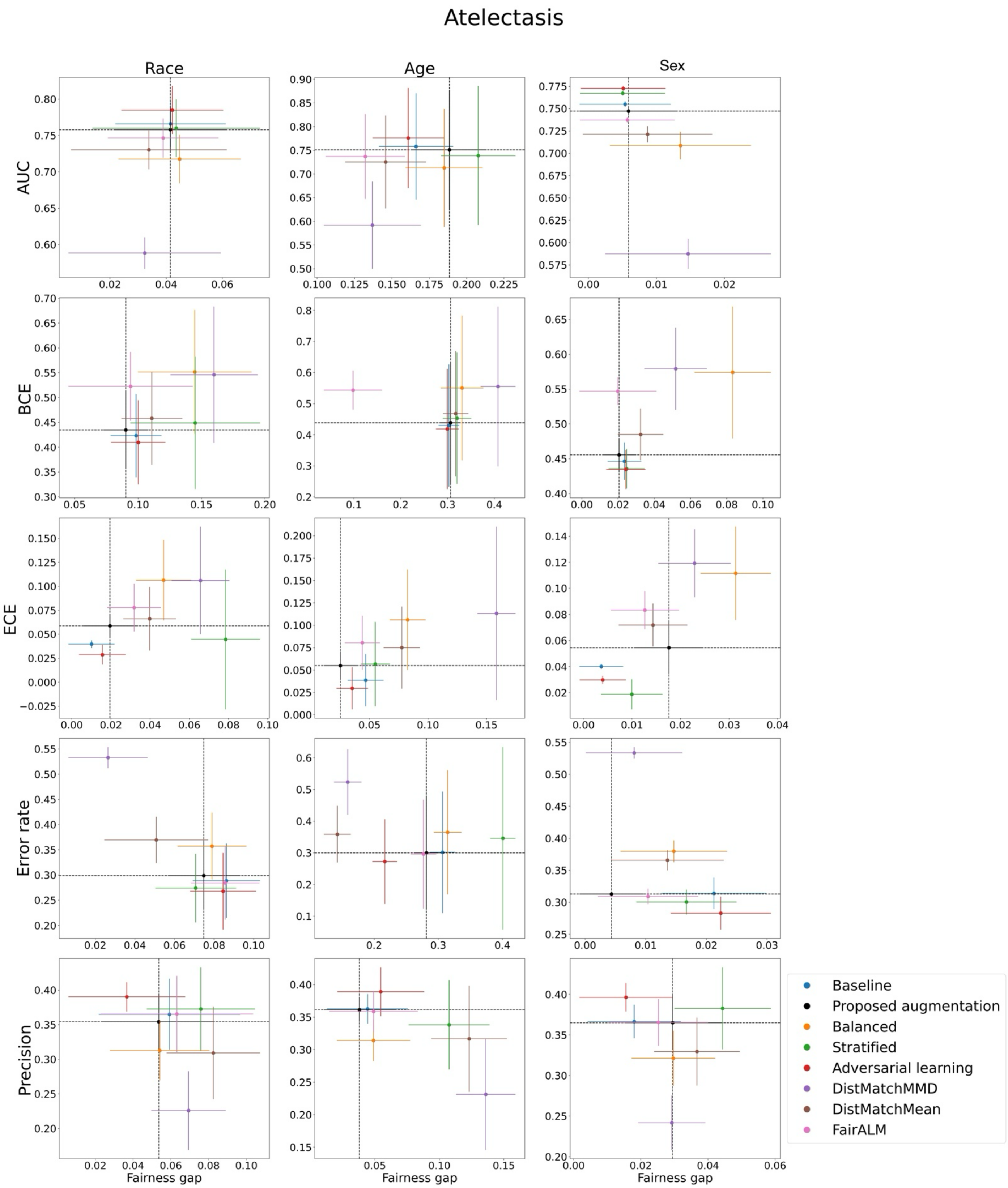


Figure E1. The model performance and fairness gap for identifying Atelectasis from CXR images in different race, age, and sex groups.

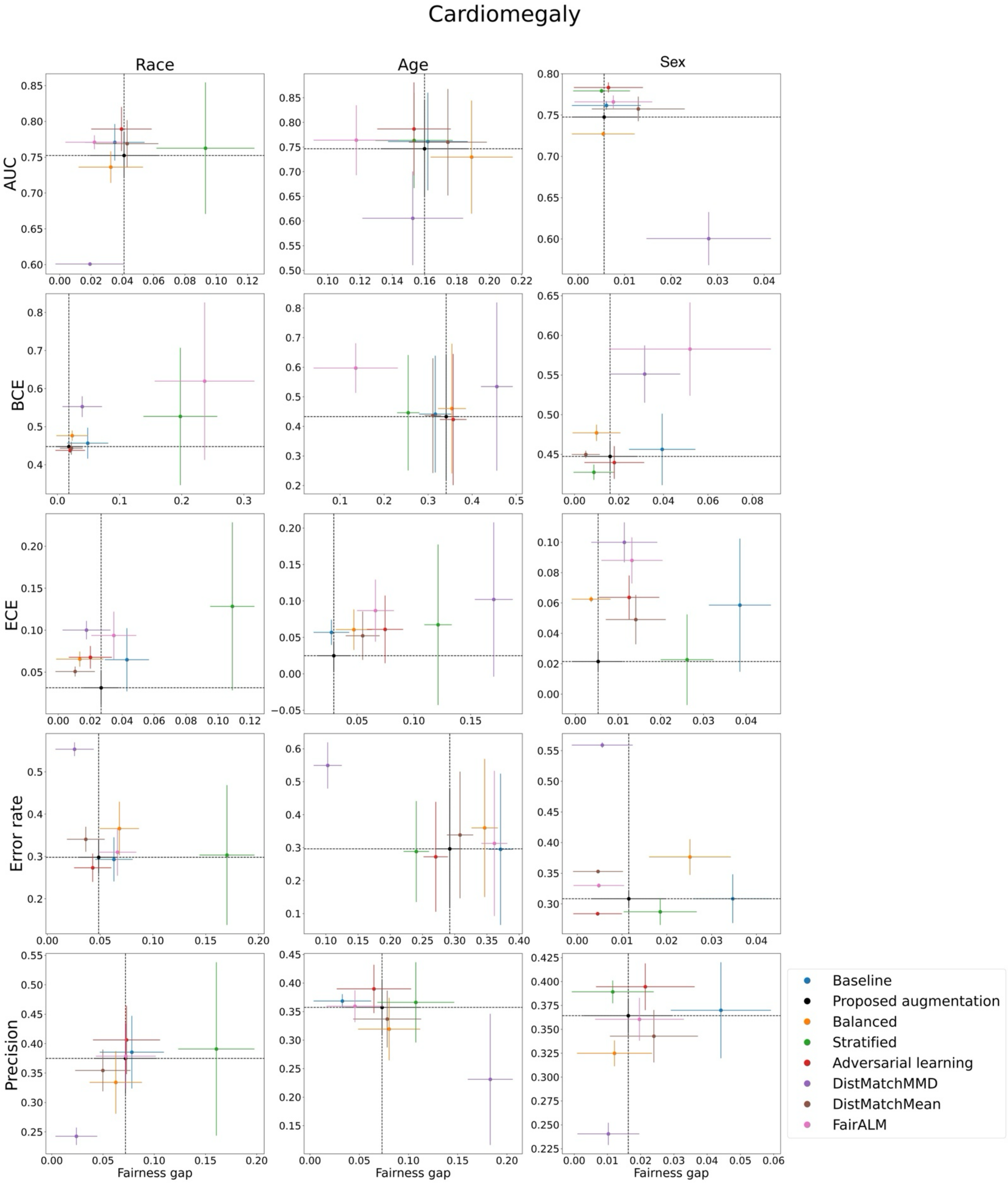


Figure E2. The model performance and fairness gap for identifying Cardiomegaly from CXR images in different race, age, and sex groups.

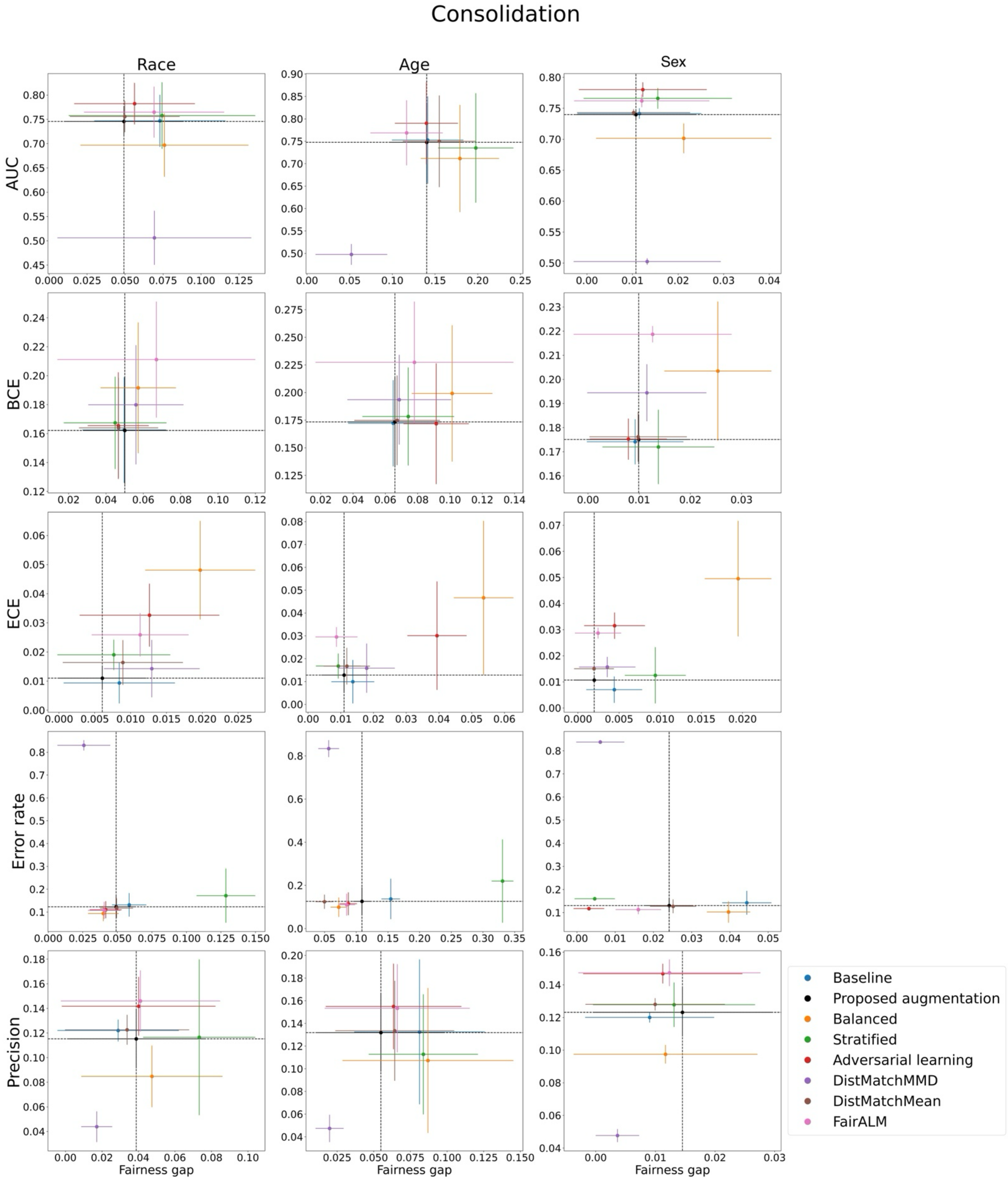


Figure E3. The model performance and fairness gap for identifying Consolidation from CXR images in different race, age, and sex groups.

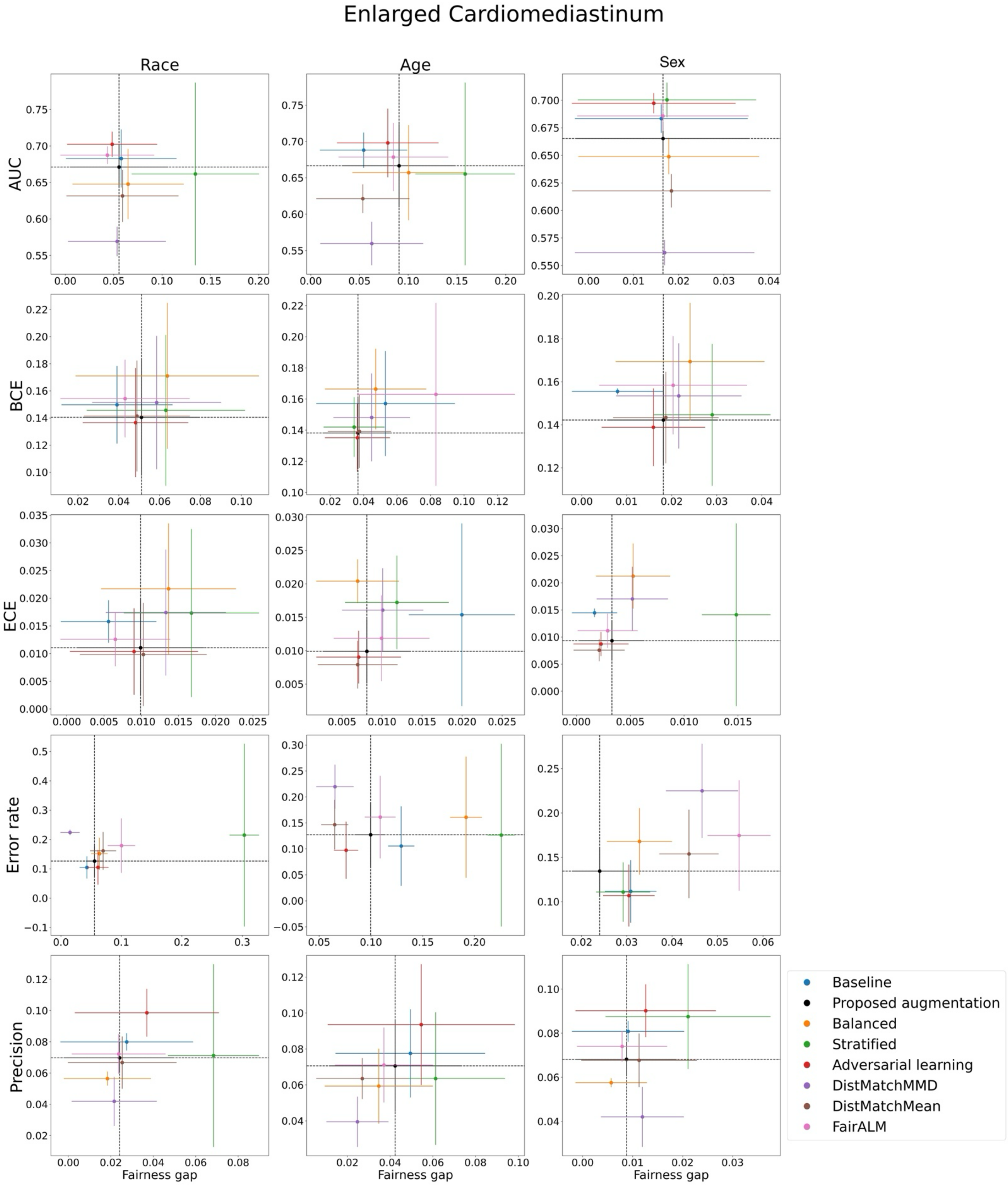


Figure E4. The model performance and fairness gap for identifying Enlarged Cardiomediastinum from CXR images in different race, age, and sex groups.

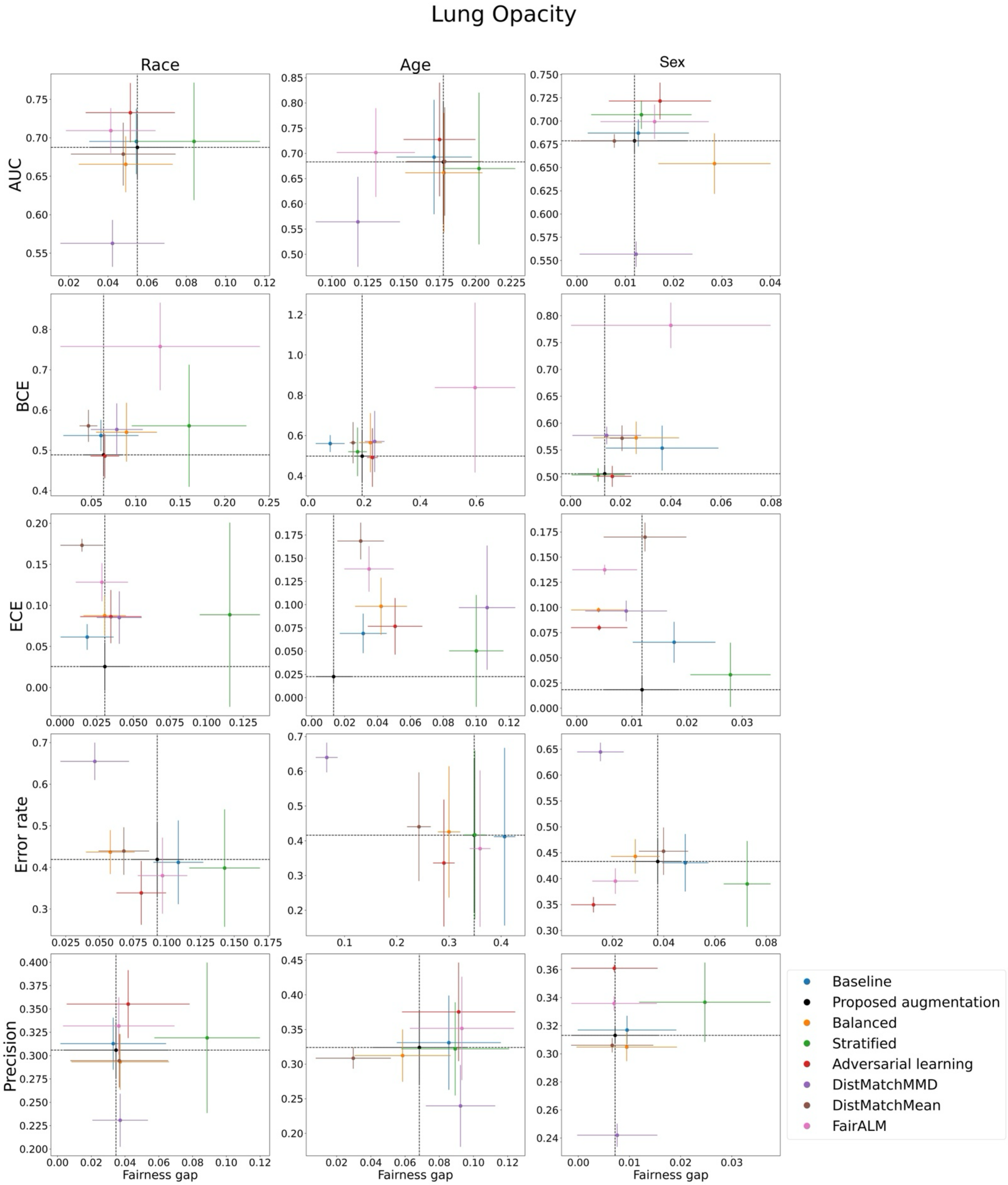


Figure E5. The model performance and fairness gap for identifying Lung Opacity from CXR images in different race, age, and sex groups.

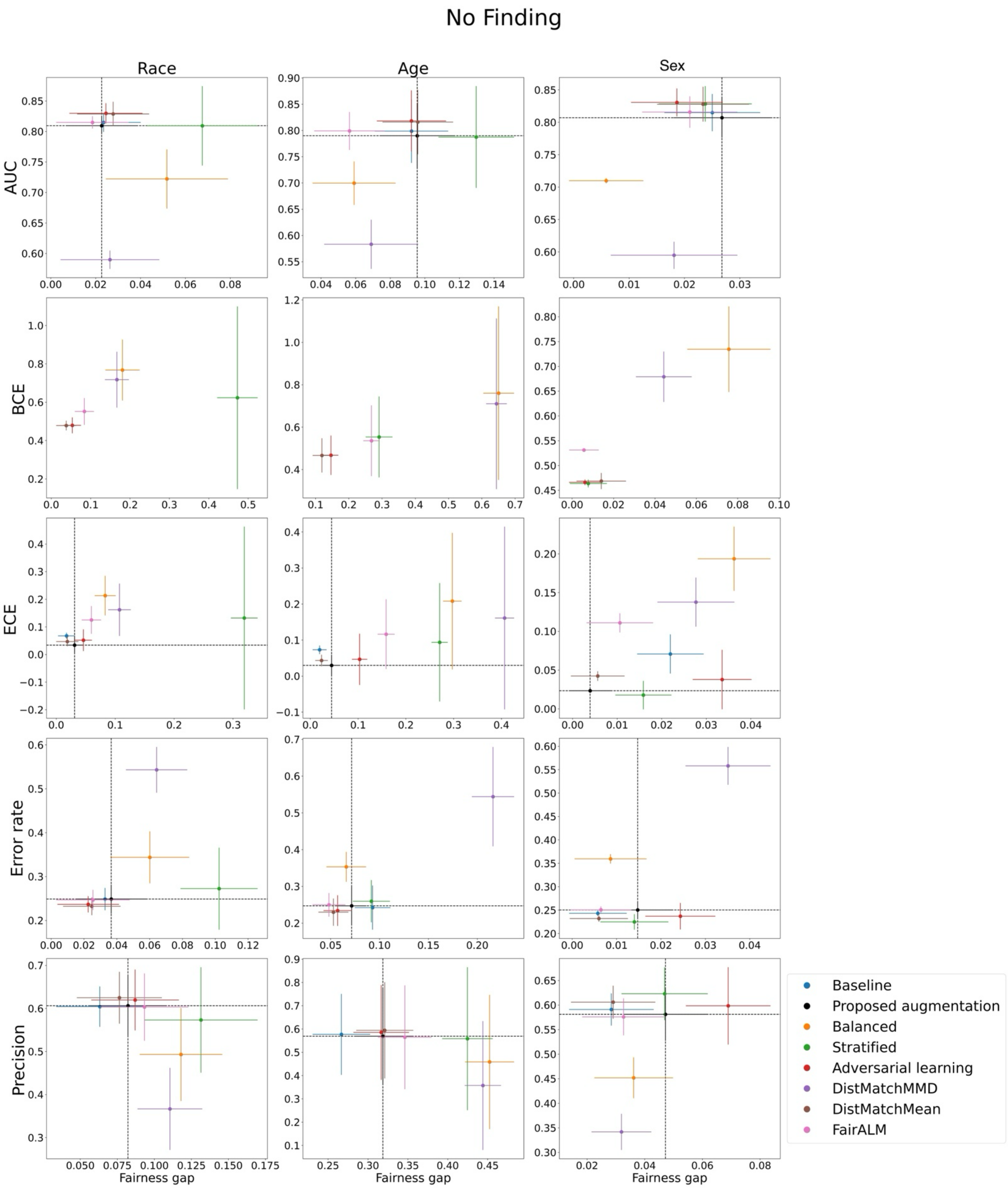


Figure E6. The model performance and fairness gap for identifying No finding from CXR images in different race, age, and sex groups.

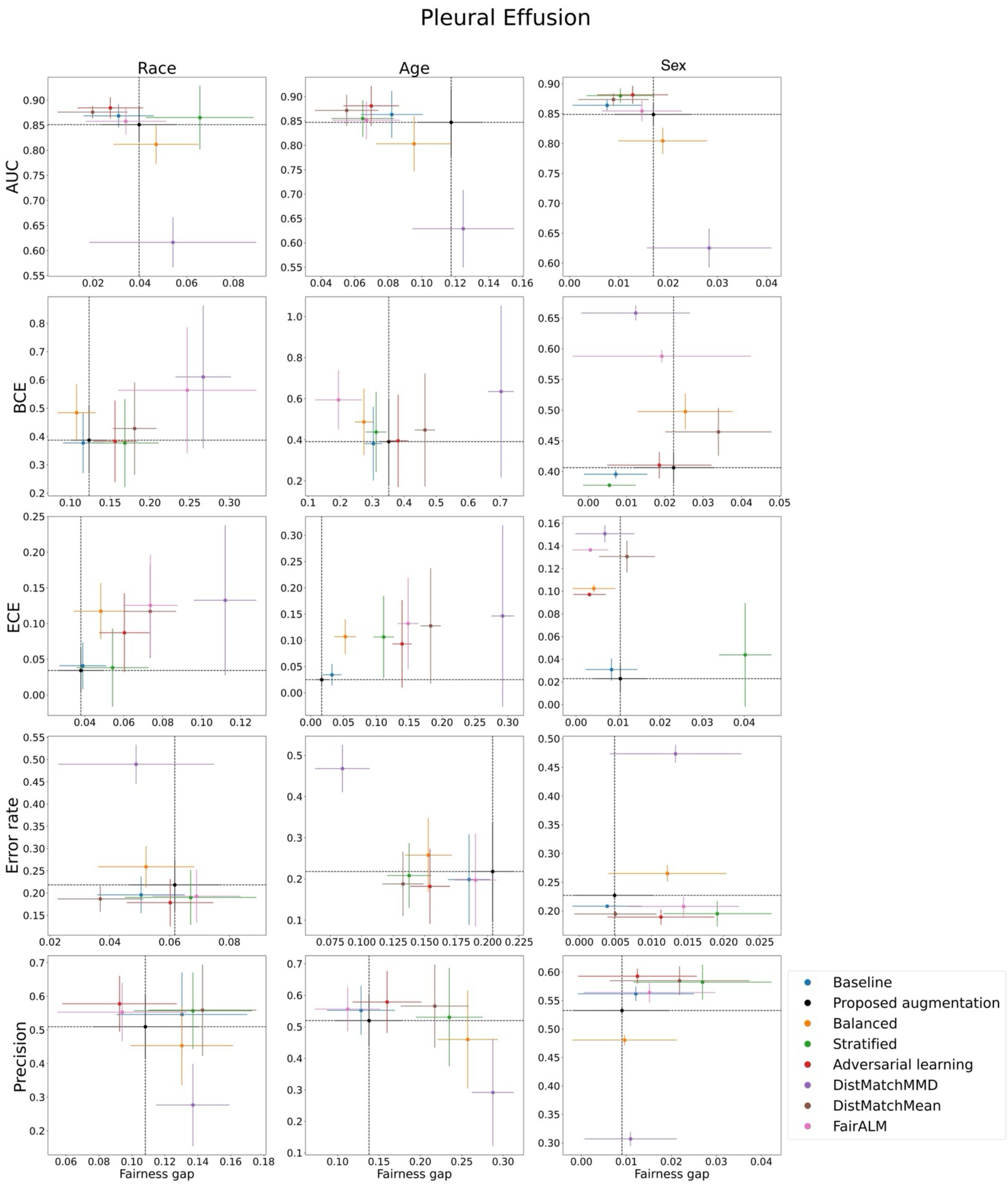


Figure E7. The model performance and fairness gap for identifying Pleural Effusion from CXR images in different race, age, and sex groups.

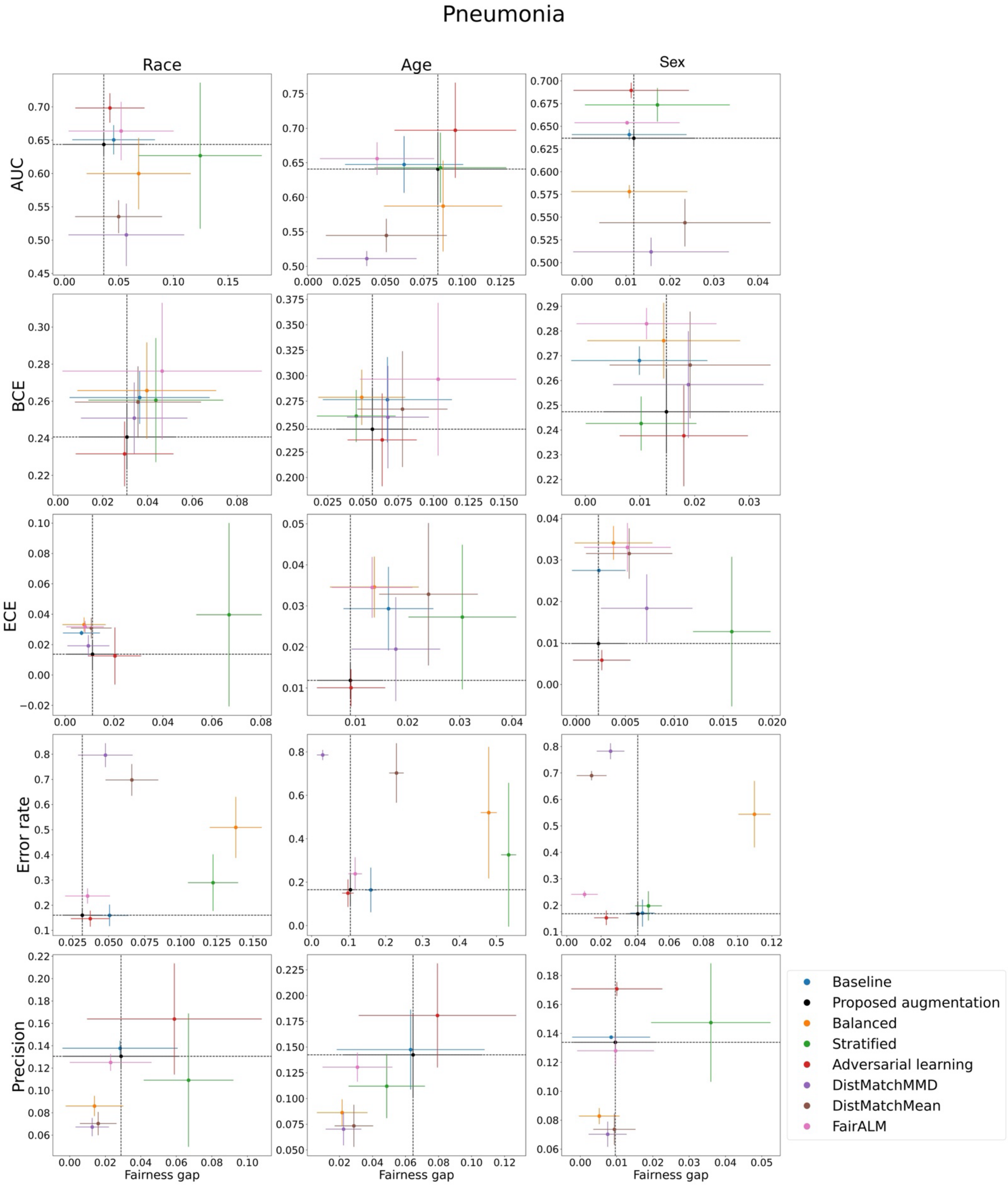


Figure E8. The model performance and fairness gap for identifying Pneumonia from CXR images in different race, age, and sex groups.

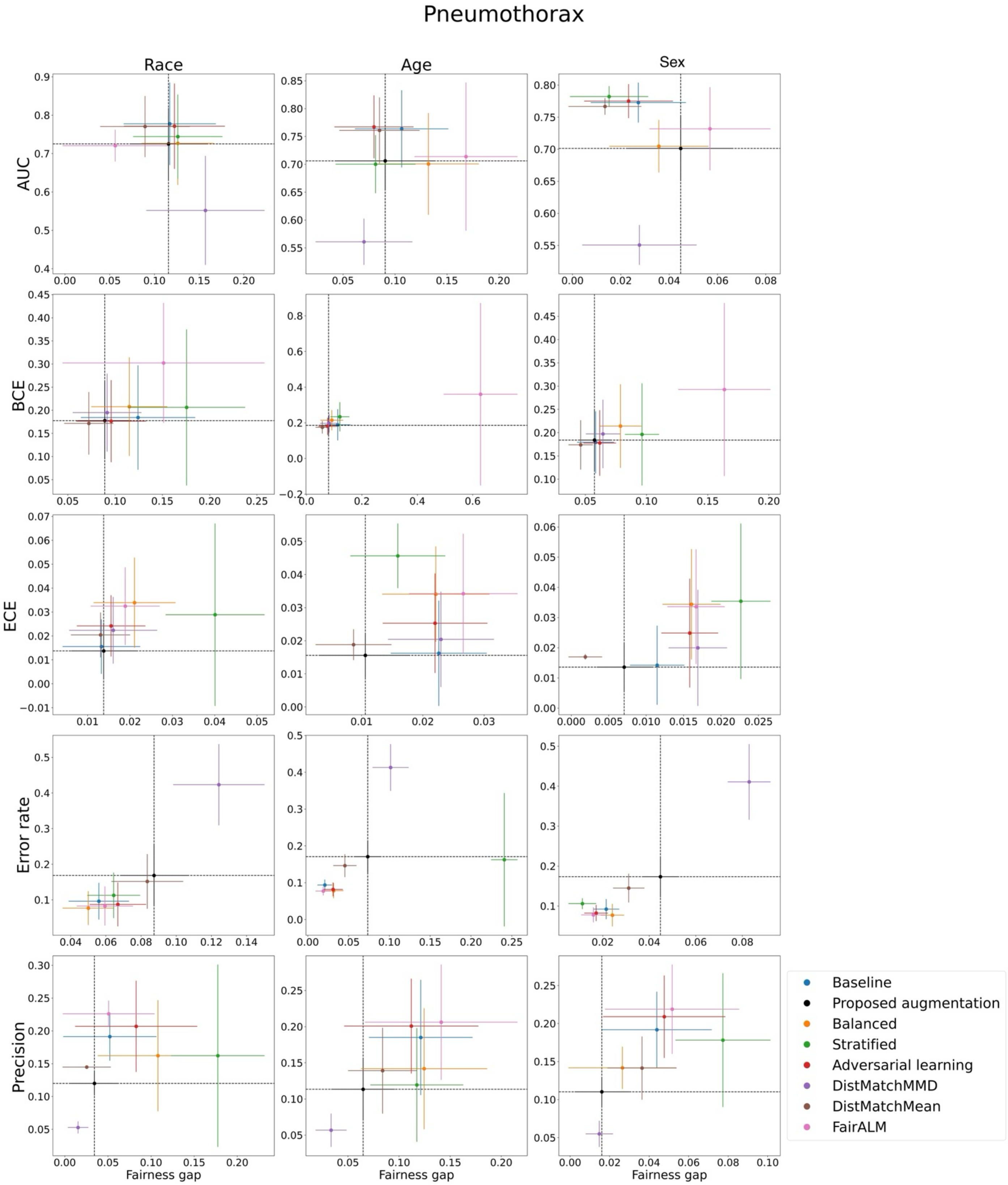


Figure E9. The model performance and fairness gap for identifying Pneumothorax from CXR images in different race, age, and sex groups.

## F. Comparison of task transfer using different model architecture and external data

The table shows the AUCs of demographic attribute prediction using the hidden features of the image label detection model. The lower values indicate that the model trained for radiological label detection used less demographic information.

Table F1. Comparison in task transfer experiment.

| **Train- and test-time augmentation** | **Race** | **Age** | **Sex** |
|---|---|---|---|
| DenseNet121 architecture and the MIMIC CXR dataset | | | |
| w/o | 0.692 [0.686-0.698] | 0.670 [0.666-0.674] | 0.880 [0.876-0.883] |
| w/ | 0.662 [0.656-0.668] | 0.665 [0.661-0.668] | 0.777 [0.772-0.781] |
| ResNet50 architecture and the MIMIC CXR dataset | | | |
| w/o | 0.697 [0.691-0.703] | 0.726 [0.723-0.730] | 0.831 [0.827-0.835] |
| w/ | 0.632 [0.626-0.639] | 0.645 [0.641-0.648] | 0.728 [0.724-0.733] |
| DenseNet121 architecture and the CheXpert CXR dataset | | | |
| w/o | 0.722 [0.915-0.729] | 0.583 [0.579-0.587] | 0.895 [0.891-0.900] |
| w/ | 0.577 [0.568-0.585] | 0.554 [0.549-0.0.559] | 0.715 [0.708-0.722] |
| ResNet18 architecture and the ADNI brain MRI dataset | | | |
| w/o | N/A | 0.566 [0.478-0.653] | 0.560 [0.475-0.644] |
| w/ | N/A | 0.480 [0.389-0.570] | 0.508 [0.420-0.596] |

## G. Model interpretation

We presented saliency maps and histograms of gradients for each radiological finding to demonstrate that the proposed augmented data does not impact the model's ability to predict radiological findings.

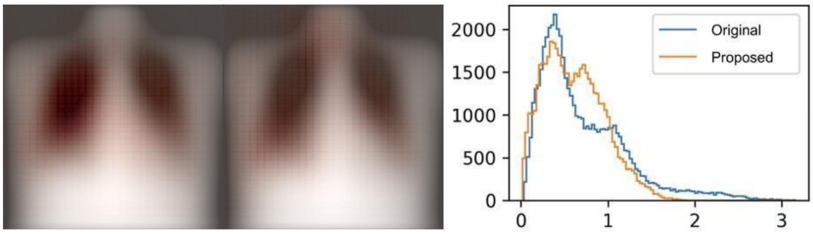


Figure G1. Atelectasis (Original model vs. Proposed model)

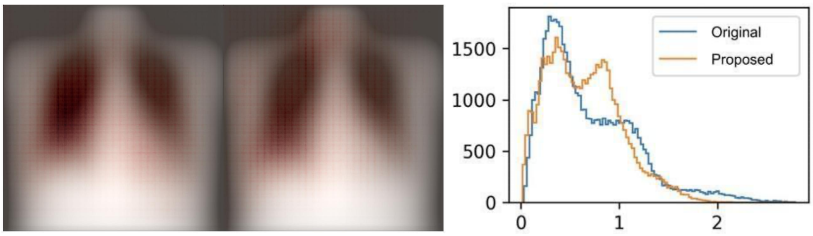

Figure G2. Cardiomegaly (Original model vs. Proposed model)

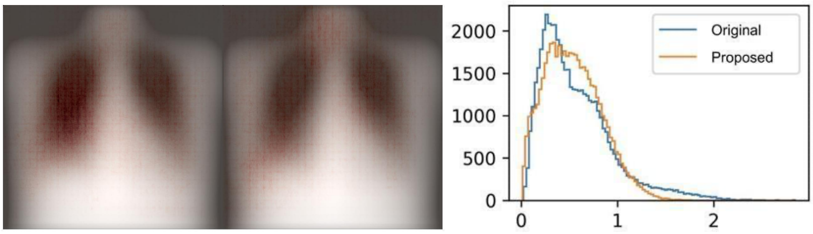

Figure G3. Consolidation (Original model vs. Proposed model)

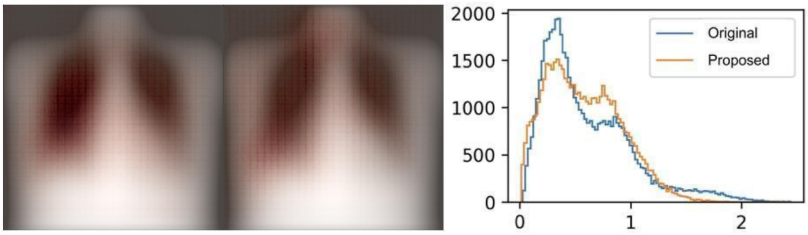

Figure G4. Edema (Original model vs. Proposed model)

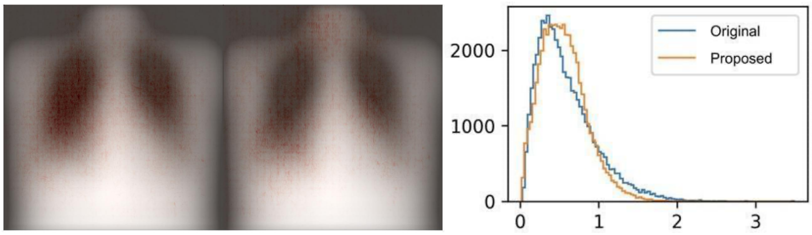

Figure G5. Enlarged Cardiomediastinum (Original model vs. Proposed model)

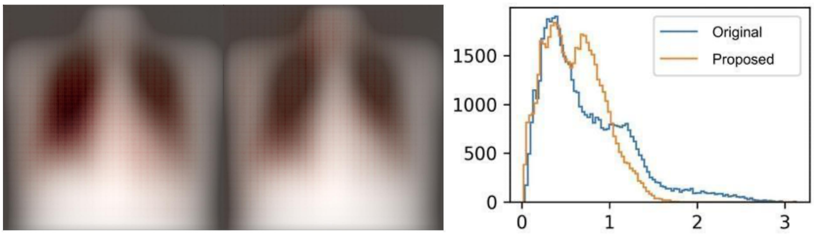

Figure G6. Lung Opacity (Original model vs. Proposed model)

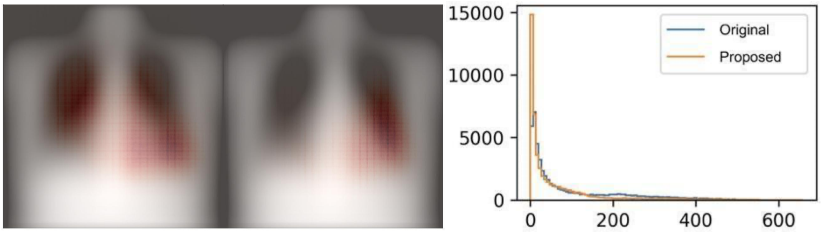

Figure G7. No Finding (Original model vs. Proposed model)

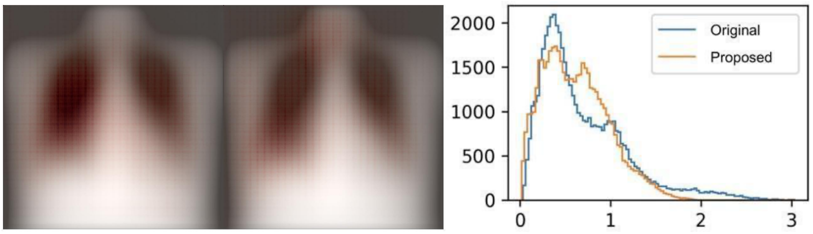

Figure G8. Pleural Effusion (Original model vs. Proposed model)

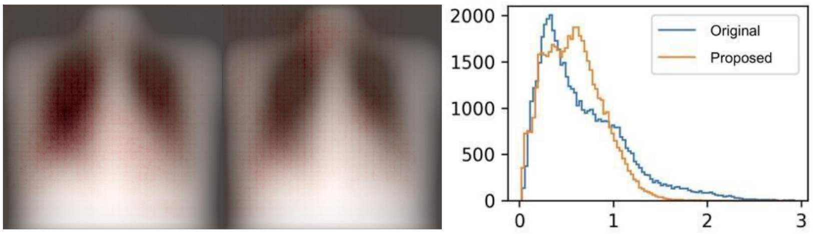

Figure G9. Pneumonia (Original model vs. Proposed model)

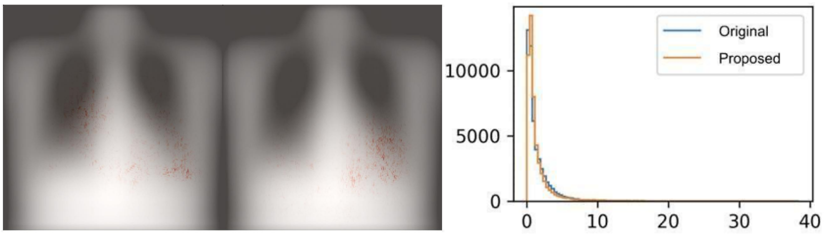

Figure G10. Pneumothorax (Original model vs. Proposed model)

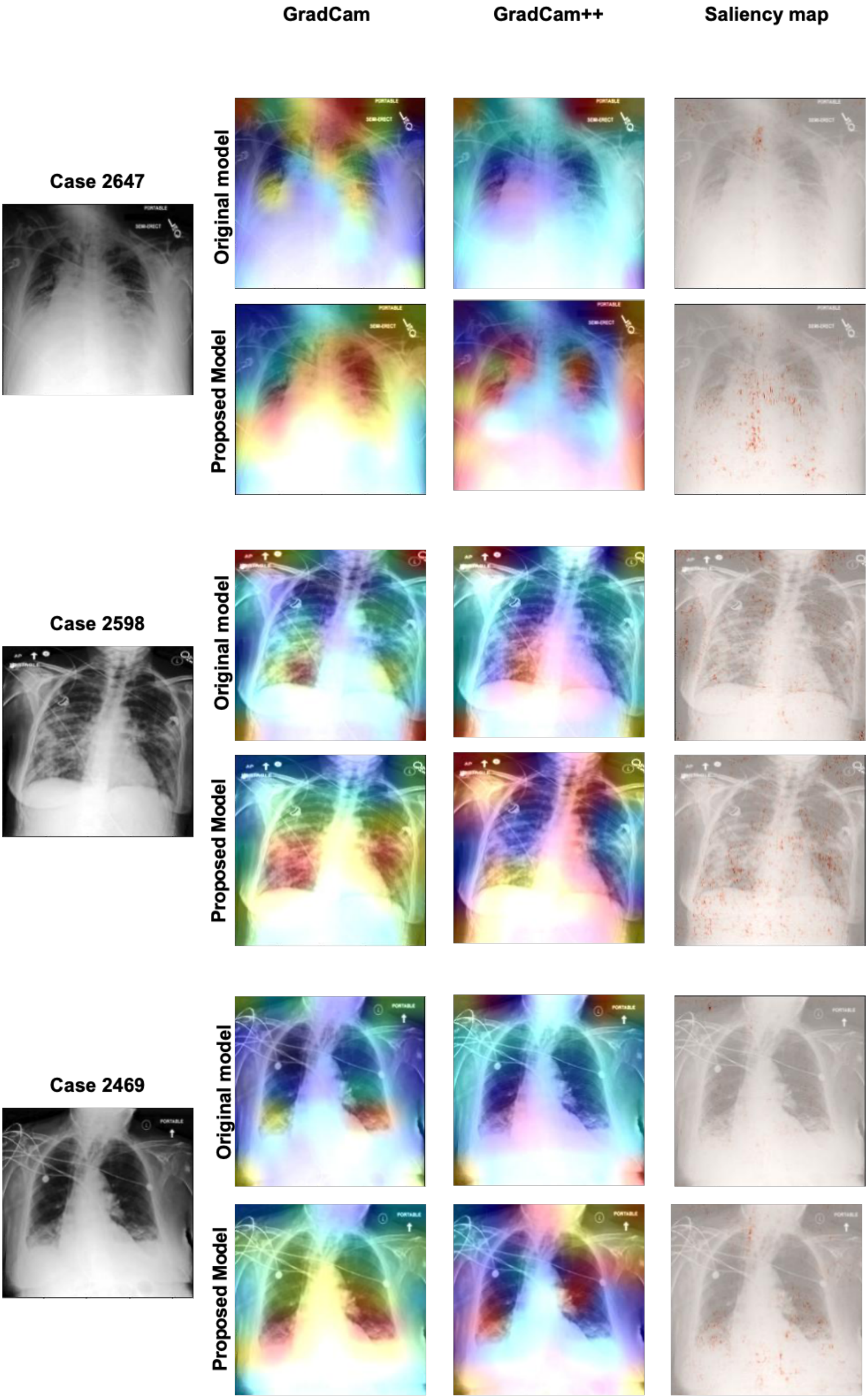

Figure G11. Comparison of different feature attribution methods.

## H. Data distribution of train/validation/test

The following two tables show the data distribution of the train/validation/test splits of the MIMIC-CXR and ADNI brain MRI data.

Table H1. Data distribution for MIMIC-CXR.

| **Attributes** | MIMIC-CXR training | MIMIC-CXR validation | MIMIC-CXR test |
|---|---|---|---|
| **# Images** | 116,405 | 19,339 | 58,615 |
| **# Patients** | 26,962 | 4,511 | 13,480 |
| **Race** | | | |
| Asian | 1127 (4.2%) | 200 (4.4%) | 614 (4.6%) |
| Black | 5374 (19.9%) | 921 (20.4%) | 2650 (19.7%) |
| White | 20461 (75.9%) | 3390 (75.1%) | 10216 (75.8%) |
| **sex** | | | |
| Female | 12897 (47.8%) | 2139 (47.4%) | 6418 (47.6%) |
| Male | 14065 (52.2%) | 2372 (52.6%) | 7062 (52.4%) |
| **Age** | | | |
| 0-40 | 3837 (14.2%) | 609 (13.5%) | 1944 (14.4%) |
| 40-60 | 8210 (30.5%) | 1339 (29.7%) | 4131 (30.6%) |
| 60-80 | 10226 (37.9%) | 1763 (39.1%) | 5106 (37.9%) |
| 80+ | 4689 (17.4%) | 800 (17.7%) | 2299 (17.1%) |

Table H2. Data distribution for ADNI MRI.

| **Attributes** | ADNI training | ADNI validation | ADNI testing |
|---|---|---|---|
| **# Images** | 765 | 187 | 243 |
| **# Patients** | 173 | 44 | 55 |
| **Race** | | | |
| Asian | 1 (0.6%) | 0 (0%) | 0 (0%) |
| Black | 14 (8.1%) | 2 (4.5%) | 5 (9.1%) |
| White | 158 (91.3%) | 41 (93.2%) | 49 (89.1%) |
| Others | 0 (0%) | 1 (2.3%) | 1 (1.8%) |
| **sex** | | | |
| Female | 84 (48.6%) | 17 (38.6%) | 26 (47.3%) |
| Male | 89 (51.4%) | 27 (61.4%) | 29 (52.7%) |

| Age | | | |
|---|---|---|---|
| 0-75 | 60 (34.7%) | 14 (31.8%) | 16 (29.1%) |
| 75+ | 113 (65.3%) | 30 (68.2%) | 39 (70.9%) |

## I. Evaluation metrics

Table I1. The evaluation metrics used in this study.

| | |
|---|---|
| AUC | Area under the curve, which is plotted with true positive rate against false positive rate. |
| BCE | The binary cross entropy loss is to calculate the entropy of the prediction and view each class separately. |
| ECE | The expected calibration error is to calculate the weighted average error of the estimated probability. |
| Error rate | Error rate is to calculate the percentage of correctly classified cases. |
| Precision | Precision is to calculate how many cases that are predicted to be positive are positive. |

## Reference


1. scikit-image: image processing in Python [PeerJ]. https://peerj.com/articles/453/?report=reader&utm_source=TrendMD&utm_campaign=PeerJ_TrendMD_1&utm_medium=TrendMD.
2. Home. *OpenCV* https://opencv.org/.
3. Seyyed-Kalantari, L., Liu, G., McDermott, M., Chen, I. Y. & Ghassemi, M. CheXclusion: Fairness gaps in deep chest X-ray classifiers. Preprint at http://arxiv.org/abs/2003.00827 (2020).
